# Deep learning to estimate gestational age from fly-to cineloop videos: a novel approach to ultrasound quality control

**DOI:** 10.1002/ijgo.15321

**Published:** 2024-01-08

**Authors:** Ambika V. Viswanathan, Teeranan Pokaprakarn, Margaret P. Kasaro, Hina R. Shah, Juan C. Prieto, Chiraz Benabdelkader, Yuri V. Sebastião, Ntazana Sindano, Elizabeth Stringer, Jeffrey S. A. Stringer

**Affiliations:** 1. Department of Obstetrics and Gynecology; University of North Carolina School of Medicine; USA; 2. Department of Biostatistics, University of North Carolina Gillings School of Global Public Health; USA; 3. UNC Global Projects – Zambia, LLC; Zambia; 4. Department of Psychiatry, University of North Carolina School of Medicine; USA

**Keywords:** Biometry, ultrasound, quality control, deep learning, gestational age, artificial intelligence

## Abstract

**Objective:**

Low-cost devices have made obstetric sonography possible in settings where it was previously unfeasible, but ensuring quality and consistency at scale remains a challenge. We sought to create a tool to reduce sub-standard fetal biometry measurement while minimizing care disruption.

**Methods:**

We developed a deep learning AI model to estimate gestational age (GA) in the second and third trimester from fly-to cineloops – brief videos acquired during routine ultrasound biometry – and evaluated its performance in comparison to expert sonographer measurement. We then introduced random error into fetal biometry measurements and analyzed the AI model’s ability to flag grossly inaccurate measurements such as those that might be obtained by a novice.

**Results:**

The mean absolute error (MAE) of our model (± standard error) was 3.87 ± 0.07 days, compared to 4.80 ±0.10 days for expert biometry (difference −0.92 days; 95% CI, −1.10 to −0.76). Based on simulated novice biometry with average absolute error of 7.5%, our model reliably detected cases where novice biometry differed from expert biometry by 10 days or more, with an area under the receiver operating characteristics curve of 0.93 (95% CI, 0.92, 0.95), sensitivity of 81.0% (95% CI, 77.9, 83.8), and specificity of 89.9% (95% CI, 88.1, 91.5). These results held across a range of sensitivity analyses, including where the model was provided sub-optimal truncated fly-to cineloops.

**Conclusions:**

Our AI model estimated GA more accurately than expert biometry. Because fly-to cineloop videos can be obtained without any change to sonographer workflow, the model represents a no-cost guardrail that could be incorporated into both low-cost and commercial ultrasound devices to prevent reporting of most gross GA estimation errors.

## Introduction

Fetal ultrasound is vital to modern obstetrical practice. In its 2016 antenatal care (ANC) guidelines, the World Health Organization (WHO) recommends a single ultrasound scan before 24 weeks of gestation to accurately estimate gestational age (GA), enhance the detection of fetal anomalies and multiple pregnancies, minimize the induction of labor for post-term pregnancy, and improve a woman’s pregnancy experience.[1] When performed correctly, fetal ultrasound biometry offers more accurate GA assessment than the last menstrual period (LMP). [2] Greater accuracy in GA estimation allows for better decision making around critical GA thresholds (e.g. viability, term) and for providers to effectively schedule the various aspects of an individual patient’s care.

Recent technological advances have brought a proliferation of low-cost, point-of-care ultrasound devices, increasing the accessibility of ultrasound imaging in low-resource settings.[3] However, implementing and expanding the WHO’s universal ultrasound recommendations in such settings faces numerous logistical, infrastructural, human capacity, and financial challenges. A primary concern is the training and credentialing of healthcare workers, such as midwives and nurses, who will operate these devices.[4,5]

Although training healthcare workers to perform basic sonography tasks like fetal biometry is achievable, ensuring the quality and consistency of ultrasound examinations at scale is a more complex task.[6,7] A system capable of automatically monitoring the quality of ultrasound examinations while producing minimal disturbance to care delivery on a busy obstetrics service would provide significant value in addressing this challenge.

## Methods

The Fetal Age Machine Learning Initiative (FAMLI) is an ongoing prospective cohort study in Chapel Hill, North Carolina, and Lusaka, Zambia. Its goal is to develop tools to further ultrasound access in low-resource settings. Details of its methodology have been published elsewhere.[8] All participants provided written informed consent, were at least 18 years of age, and had a singleton, viable pregnancy. Although the FAMLI protocol requires only one visit, participants were allowed to return for repeat scans as frequently as bi-weekly through delivery. The study protocol has ongoing approval from the University of North Carolina Chapel Hill Office of Human Research Ethics and the University of Zambia Biomedical Research Ethics Committee.

### Sonography

Sonographers for the FAMLI study were certified, obstetrics-trained, and credentialed by the relevant authorities in their countries. All studies described in the current analysis were completed on a cart-based commercial ultrasound device (Table S1). Fetal biometry was measured at each visit. Beyond 14 weeks of gestation this included biparietal diameter (BPD), head circumference (HC), trans-cerebellar diameter (TCD), abdominal circumference (AC), and femur length (FL). Each structure was measured twice.

**Figure 1:**
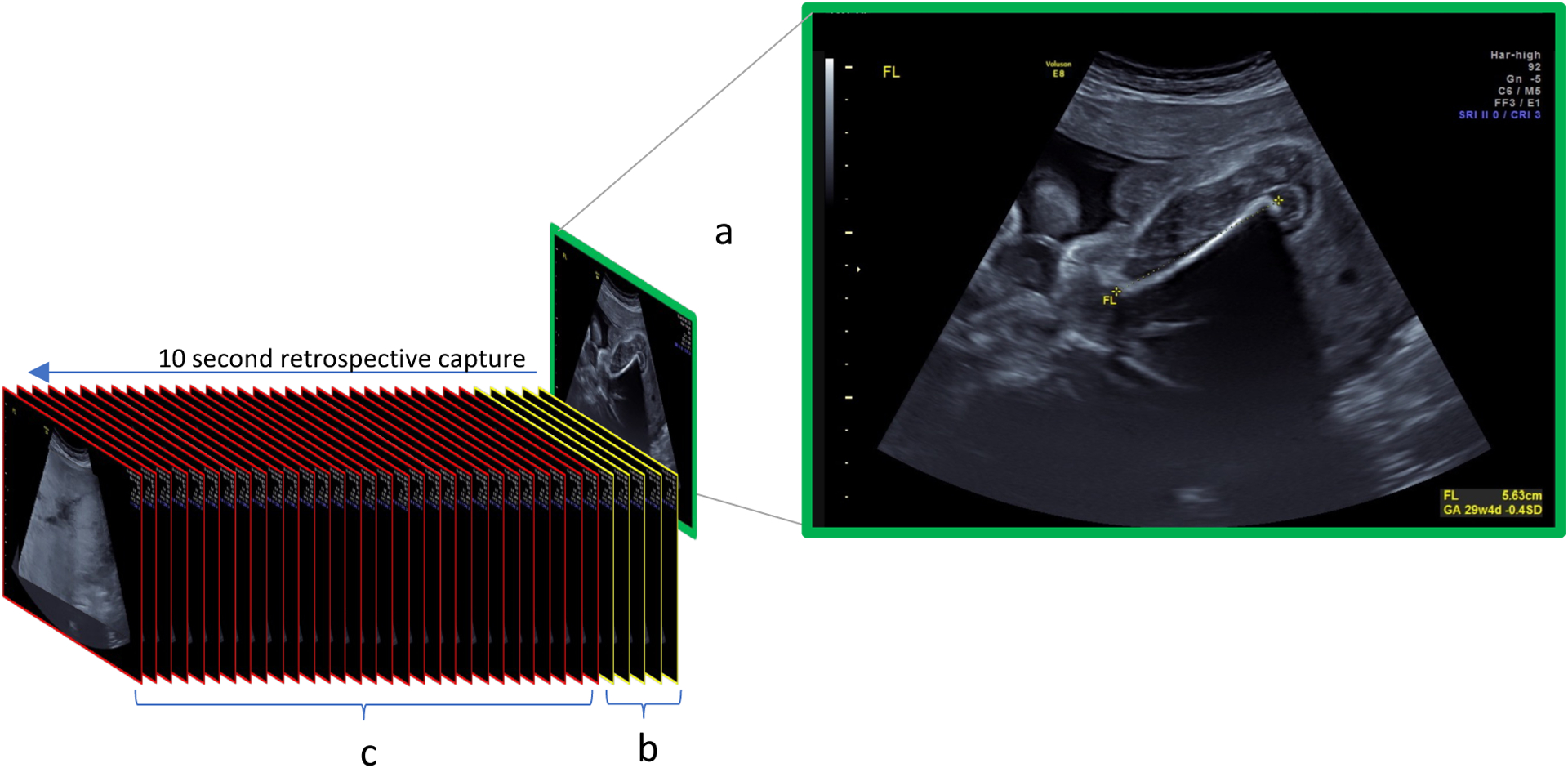
Components of a fly-to cineloop video a The last frame of a fly-to cineloop is what the sonographer freezes and measures. b The frames immediately preceding the last frame are often very similar to the last frame. c The earlier frames in a fly-to cineloop may contain other structures adjacent to the structure of interest or they may contain no usable information

Alongside each biometry measurement, the sonographer also saved a fly-to cineloop. Fly-to cineloops are retrospectively captured video sequences of the 10 seconds prior to a user navigating the ultrasound probe to obtain a head, abdomen, or femur view for measurement. As such, the last frame in a fly-to cineloop always contains a known measurable structure in standard view (Figure 1). In practice, the sonographer does not have to change the workflow to obtain a fly-to cineloop. It is captured in the background automatically through programming of the ultrasound machine.

The “ground-truth” GA was determined as outlined in a prior publication.[8] In Zambia, where participants often present later in pregnancy, self-reported LMP is not an accurate measure of GA.[9] Thus, we assigned ground truth GA based solely upon the first ultrasound scan using the Intergrowth-21^st^ GA formula.[9,10] In the North Carolina cohort, we dated by the *best obstetric estimate* as defined by the American College of Obstetricians and Gynecologists, which incorporates both LMP and early ultrasound GA using the Hadlock IV formula.[11,12] The one exception to this approach was in those who conceived by *in vitro* fertilization, in which case we assigned ground truth GA by known embryo age and transfer date.

### Training, Tuning, and Testing Data Sets

**Figure 2:**
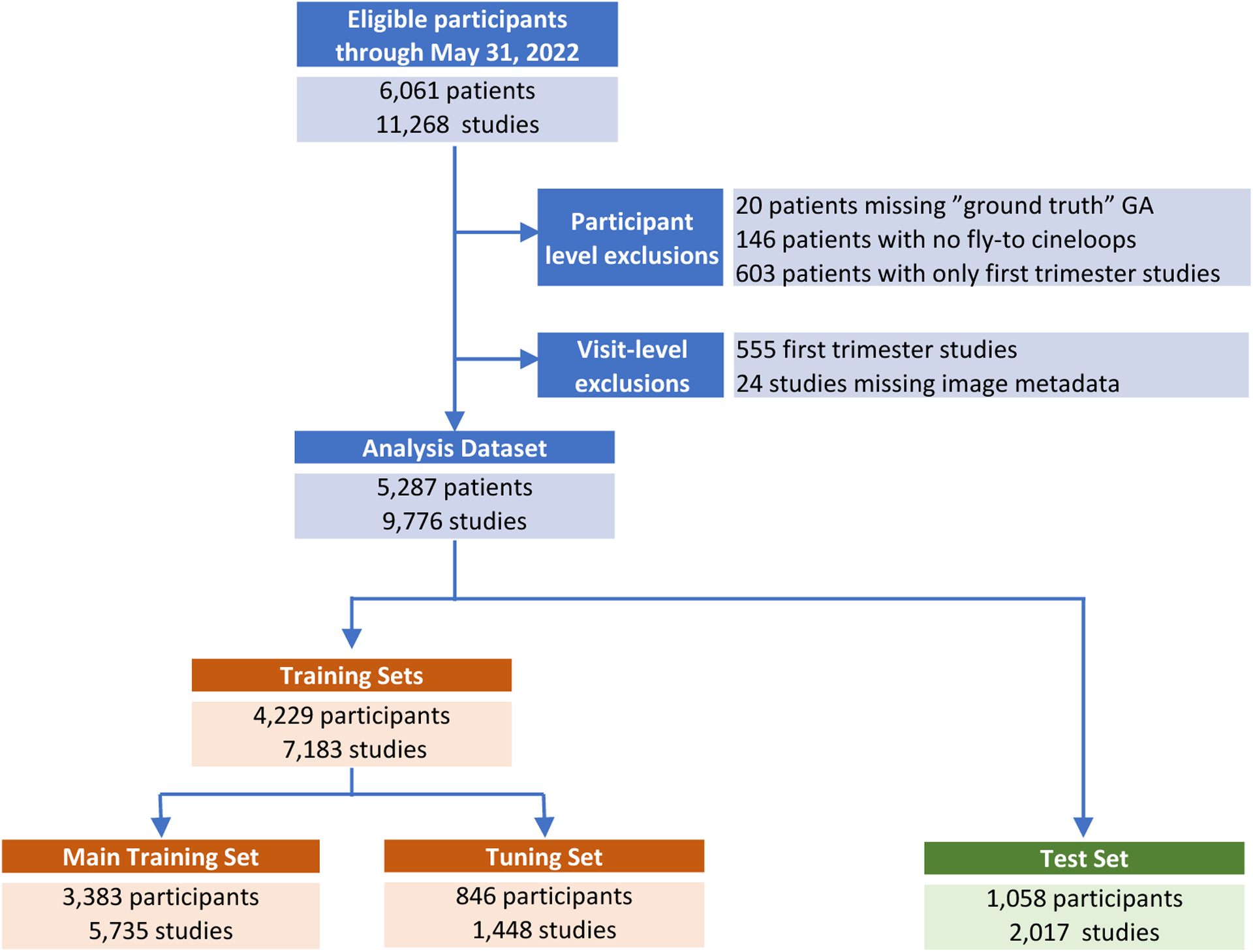
Study flow chart After applying participant and visit-level exclusions, we created a training set to develop and tune the deep learning model and a test set to assess its performance. To be eligible for inclusion in a test set a participant must have been dated by a prior scan or in vitro fertilization (IVF). Additionally, a scan must include full biometry (head circumference, biparietal diameter, abdominal circumference, and femur length) as well as a full set of fly-to scans (head defined as either head circumference or biparietal diameter fly-to, abdominal circumference fly-to, and femur fly-to). TCD fly-to scans were not included in the test set. The test set was selected at random from among all eligible participants. Train and tuning sets were created from all remaining participants.

This analysis used FAMLI study data collected between September 2018 and May 2022. At the participant level, we excluded those for whom we could not establish a reliable “ground-truth” GA and those who contributed only first trimester scans to the FAMLI study. At the study level, we excluded study visits for all first trimester scans, those where no fly-to cineloops were collected, and those with insufficient ultrasound metadata (e.g., image pixel spacing) for analysis. After applying these participant- and study-level exclusions, our analysis dataset comprised 9,776 studies from 5,287 unique participants (Figure 2).

To create our training, testing, and tuning sets, we followed the principled approach outlined previously.[8] First, we created the test set that would be used to assess the model’s performance. To be included in the test set, a participant’s pregnancy must have been already dated as described above. We also required test set members to have a full complement of biometry measurements including head circumference (HC), biparietal diameter (BPD), femur length (FL), and abdominal circumference (AC) and a full set of fly-to cineloops (head, abdomen, and femur). A head fly-to cineloop could be either a BPD fly-to or an HC fly-to (since both are axial views of the trans-thalamic plane). We additionally did not include TCD fly-to cineloops in our test set because measuring TCD is not a standard component of fetal biometry in most low-resource settings. After limiting test set eligibility to the above criteria, we used simple random sampling at the participant level to create a test set that comprised 20% of cohort participants (Figure 2).

Among those who remained, we performed a second random split in a 4:1 ratio to create our training set and tuning set, respectively. The training set was used to establish model weights and the tuning set was used to set model hyperparameters. We also used the tuning set to establish cut-points along the receiver operating characteristics curve (ROC) at which to assess deep learning model performance in the test set (see below).

### Deep Learning Model Architecture

Our deep learning model received fly-to cineloop for input and provided a GA estimate as an output. Details of the model architecture have been previously published.[8]

### Statistical analysis of model GA estimation

We evaluated the accuracy of the deep learning model and of standard biometry by comparing the GA estimated by each modality on the day of the study to the patient’s previously established ground-truth GA.[11] The difference between each estimate and the ground truth was calculated as the absolute error of the prediction and expressed for each approach as mean absolute error with standard error (MAE ± SE). We compared MAE of the two approaches with a paired t-test and constructed a 95% confidence interval (CI) for the difference between MAEs. We also compared the MAE of the deep learning model versus standard biometry in subsets by trimester (2^nd^ trimester defined as 98 to 195 days, 3^rd^ trimester defined as 196 days or more). We used the empirical cumulative distribution function (CDF) to compare the absolute error of the deep learning model versus biometry and used the McNemar test to estimate the proportion of studies where the absolute error was less than 10 days for each GA estimate approach. The Wald-type 95% CI was used to compute the difference in proportions. These statistical analyses were completed on the test set. Statistical analyses were additionally completed on the sensitivity test sets as presented in Table S3 and S4. Statistical analyses were conducted in R version 4.2.[13]

### Using the model to identify gross error

**Figure 3.**
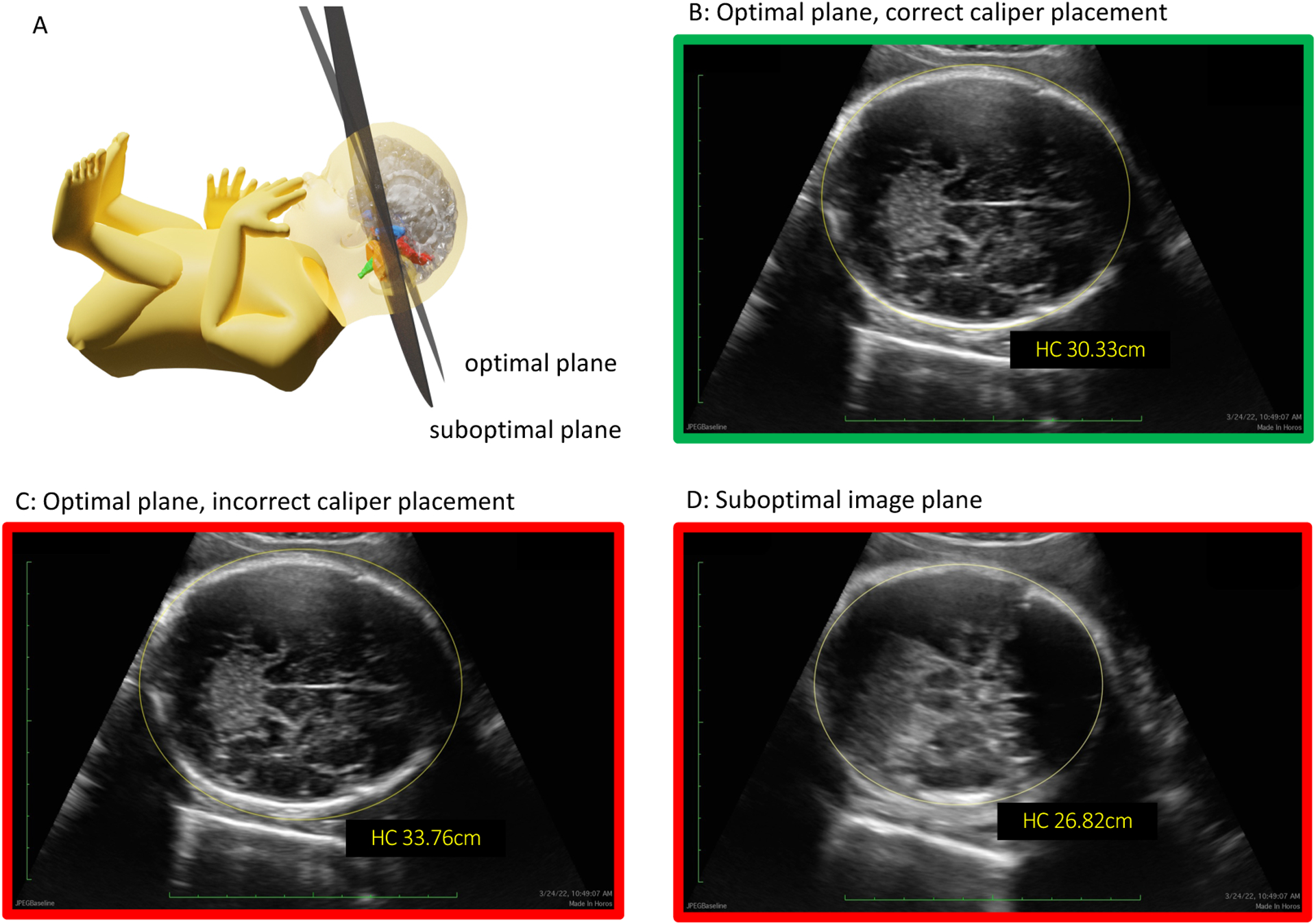
Sources of novice sonographer biometry error Panel A shows optimal and suboptimal image capture. Panel B shows optimal image for measuring head circumference with correct caliper placement. Biometry errors can result from either incorrect caliper placement (Panel C) or from capturing an imperfect plane that distorts the structure of interest (Panel D).

Evaluating the model’s ability to identify gross error is a three-step process. First, we produced a simulated novice dataset by introducing random error into our expert biometry measurements. Second, we defined “gross error” and used the receiver operating characteristics of the tuning set to determine cut-points for identifying 80%, 90%, and 95% of gross errors. Third, we applied the cut-points identified in step two to our test set and calculated test performance characteristics. Details follow here:

The International Society of Ultrasound in Obstetrics and Gynecology has published detailed criteria for obtaining standardized planes for measuring fetal biometry and these views can be challenging for inexperienced sonographers.[14] Erroneous biometry measurements can arise from either misplacement of measurement calipers on a correct image or from placing calipers “correctly” on a suboptimal view (Figure 3).

The interobserver error between an expert and novice sonographer, although not widely reported, has been shown to differ according to which measurement is being captured and the training / experience of the novice.[6,15,16] Interobserver error between expert sonographers is better studied and has been shown to range between 3–6%.[17] To simulate novice biometry, we introduced a uniform random error into our biometry measurements with an average absolute error of 7.5% and explored 5% and 10% error in sensitivity analyses.

To assess our model’s ability to identify gross errors in novice biometry, we first had to define “gross error.” We chose the threshold of ±10 days because this is the discrepancy that would, according to the American College of Obstetricians and Gynecologists,[6,11] justify GA recalculation based on sonography in the mid second trimester, which is when most women in resource-limited settings present for care.[18,19]

We used the tuning set to identify cut-points along the receiver operating characteristics (ROC) curve that would capture 80%, 90%, and 95% of gross errors in novice biometry (i.e., instances where the novice sonographer’s GA estimate differed by at least 10 days from that of the expert). Once established in the tuning set, we evaluated these cut-points in the test set and report their sensitivity, specificity, and negative and positive predictive values with 95% confidence intervals as determined by the Clopper-Pearson method. We also report overall model performance as the area under the ROC curve with 95% confidence intervals defined by the DeLong method.

### Sensitivity analyses

We performed several sensitivity analyses: (a) To ensure the model’s GA estimation results were not biased by correlated measures among test set members, we restricted the test set to a single study scan per participant (selected at random). (b) To account for uncertainty in the degree of error expected from novice biometry, we investigated introduction of 5% and 10% error in addition to the base case value of 7.5%. (c) Since a ±10-day definition of “gross error” was chosen somewhat arbitrarily, we investigated the effect of tightening (±7 days) and loosening (±14 days) this threshold. (d) Finally, since it could be argued that a fly-to cineloop captured by a novice sonographer may not contain as much useful information as one captured by an expert, we simulated this possibility by truncating the final 50 frames of each fly-to cineloop (Figure S1) and recalculated model performance with these potentially sub-optimal input videos (Table S4).

## Results

The FAMLI study enrolled 6,061 participants between September 2018 and May 2022. Of these, 5,287 were included in our analysis dataset after applying exclusions (Figure 2). These participants in total contributed 9,776 ultrasound studies and 73,865 fly-to cineloop videos. Baseline characteristics of participants can be found in Table 1.

### Deep learning model performance

In the test set, the deep learning model outperformed expert biometry in estimation of GA (MAE 3.87 ± 0.07 days vs 4.80 ± 0.10 days, respectively; difference −0.92 days, 95% CI, −1.10 to −0.76). These findings held when analyzed separately by trimester (Table 2). Additionally, when error was expressed as an empirical CDF, the proportion of study scans correctly estimated within 10 days of ground truth was higher for the deep learning model than for expert biometry (95.0% vs 88.3%; difference 6.7%, 95% CI, 5.3%, 8.2%).

### Identifying gross error

**Figure 4:**
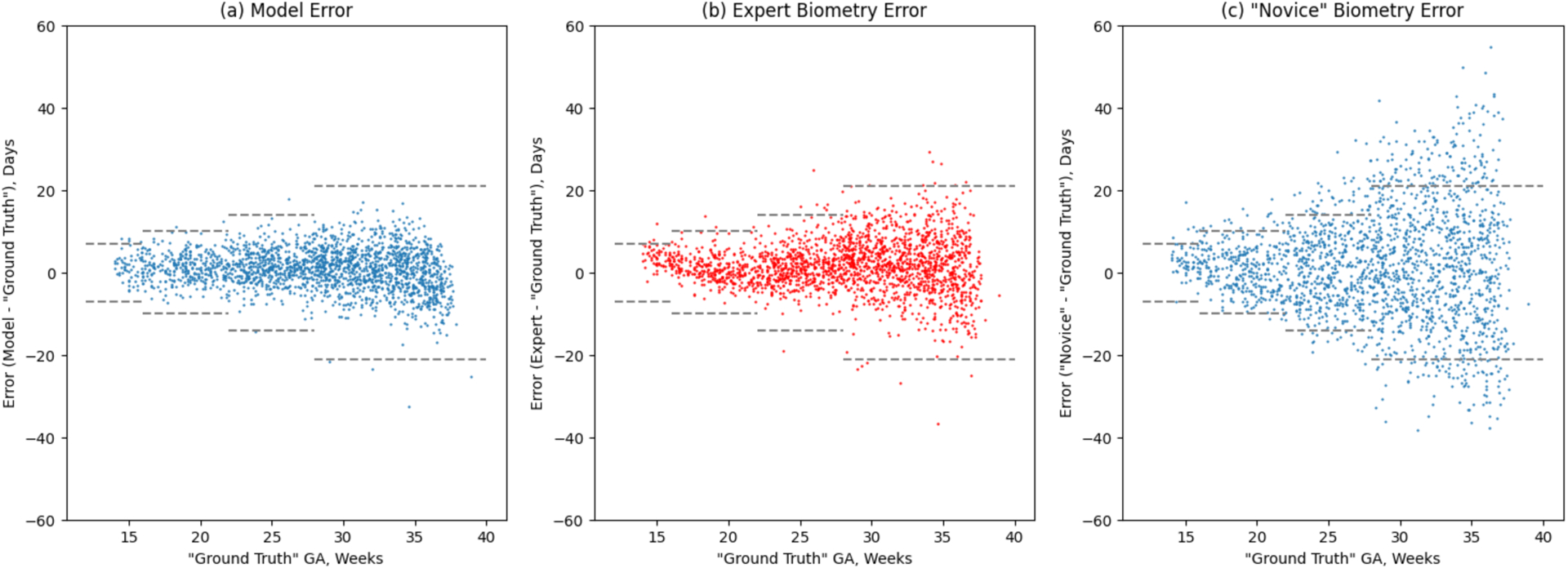
Error of deep learning model, expert biometry, and simulated novice biometry in the test set Dashed horizontal lines in each panel denote established error bounds of ultrasound biometry as defined by the American College of Obstetricians and Gynecologists.^[11]^ Novice biometry simulated by introducing 7.5% average uniform random error into expert measurements (see Methods).

**Figure 5:**
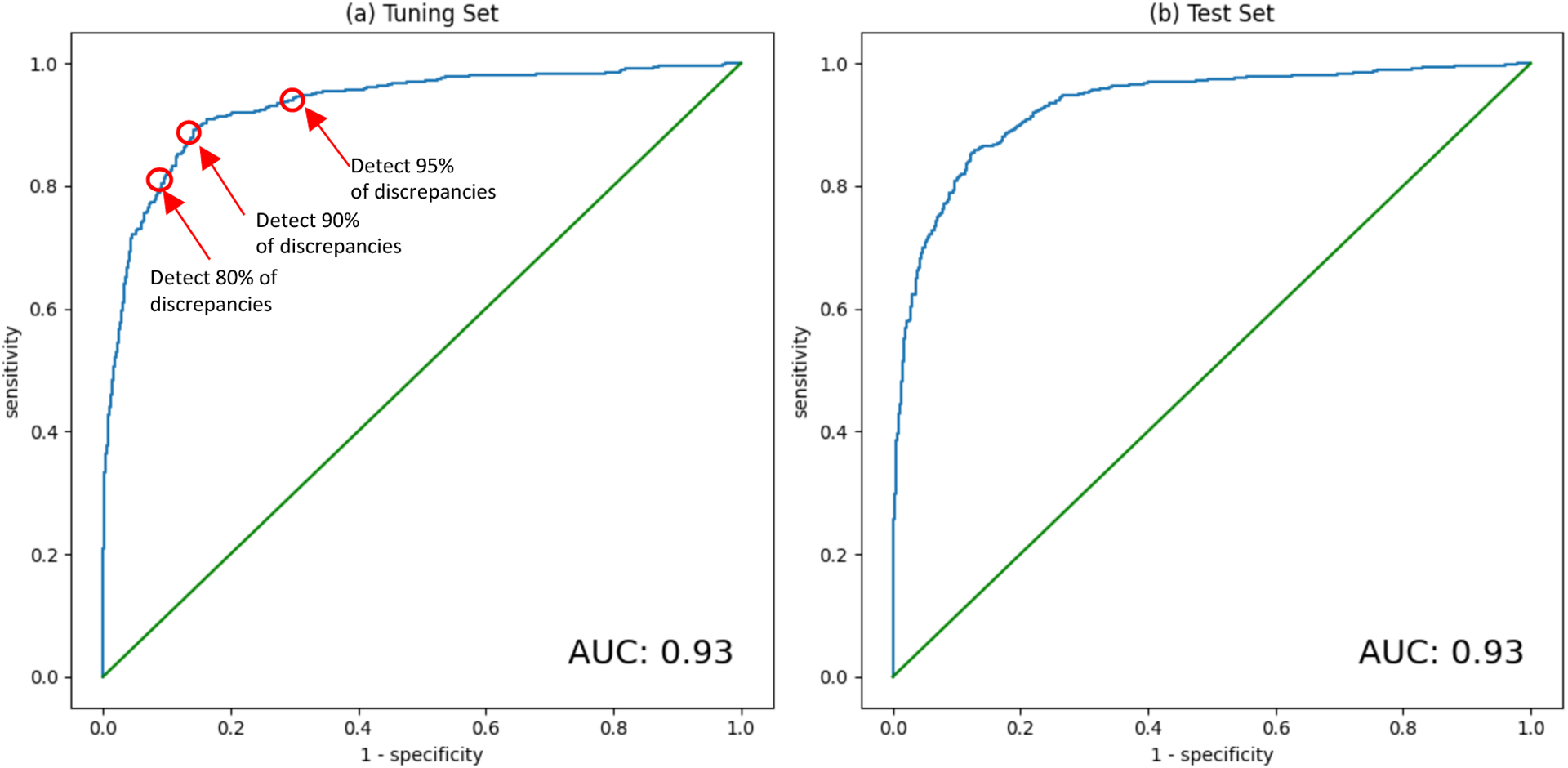
Receiver operating characteristics of deep learning model to identify 10-day discrepancy between novice and expert gestational age estimates

Identifying gross error is a three-step process as described in the methods. First, we simulated the circumstances of novice biometry by introducing random error into our expert biometry measurements. The effect of the simulated novice biometry error (7.5% under base-case assumptions) is visually apparent in Figure 4 by comparing panels b and c. Second, we used the receiver operating characteristics of the tuning set to determine cut-points for identifying 80%, 90%, and 95% of gross errors (±10 days under base-case assumptions). Through this exercise we identified 10.6 days, 8.5 days, and 6.2 days, respectively, as the relevant cut-points (Figure 5a). Third, we applied these cut-points to the test set and calculated test performance. Applying the 10.6 day cut-point to the test set resulted in sensitivity of 81.0% (95% CI, 77.9, 83.8) and specificity of 89.9% (95% CI, 88.1, 91.5). Applying the 8.5 day cut-point to the test set resulted in sensitivity of 89.4% (95% CI,87.0, 91.6) and specificity of 80.8% (95% CI, 78.6, 82.9). Applying the 6.2 day cut-point to the test set resulted in sensitivity of 96.2% (95% CI, 94.6, 97.5) and specificity of 65.0% (95% CI, 62.4, 67.6; Table 3).

### Sensitivity analyses

Because GA estimates could possibly be correlated among multiple visits from a single participant, we performed a sensitivity analysis limiting the test set to one randomly selected study per participant. Here, the deep learning model also outperformed biometry when estimating GA (MAE 3.96 ± 0.10 days vs 4.93 ± 0.14 days, respectively; difference −0.98 days, 95% CI, −1.22 to −0.73) (Table S3).

To account for uncertainty in the amount of error that might attend novice biometry, we investigated less and more error by introducing 5% and 10% uniform random error into our biometry measurements. Similarly, to understand the effect of uncertainty in the definition of gross error on our results, we varied this 7 and 14 days. This exercise demonstrated that our modeled results were not particularly sensitive to uncertainty in these two parameters (Table 4a).

Finally, we evaluated the performance of our model to flag gross errors with “novice” truncated fly-to cineloops. Here, we see that the model performance generally holds even when provided sub-optimal truncated fly-to cineloops (Table 4b, S5).

## Discussion

Fly-to cineloops are short video sequences that are easily obtained by simply recording the few seconds prior to a sonographer obtaining an ideal image plane for measurement. As such, they can be captured without altering routine ultrasound workflow. Here, we present a deep learning model that ingests a series of three fly-to cineloops (head, abdomen, femur) and accurately estimates gestational age directly from image features. Whether evaluated as overall error or as the proportion of estimates correctly classified within 10 days of a previously established GA, the deep learning model outperforms traditional biometry, a finding that is most evident in the 3^rd^ trimester (Table S2).

Whether the real, but relatively modest improvement in accuracy conferred by our approach would merit replacement of traditional polynomial formulas (e.g., Hadlock, Intergrowth-21^st^) for GA estimation is open to debate. However, in settings where there is no expert sonographer on site to provide real-time quality assurance, our AI model could additionally act as a no-cost guardrail to prevent reporting of particularly inaccurate results while not requiring perfection. This could be especially helpful as new obstetric ultrasound capacity is rapidly rolled out in low- and middle-income settings.[20] We show that with a reasonable specificity (89.4%) our model can identify 4 of 5 scans (81.0%) scans where a novice’s measurements differ by more than 10 days from that expected from an expert. The findings were robust across a range of assumptions, including the use of sub-optimal, truncated fly-to cineloops. (Table 4b, S5).

Continuous quality assessment of fetal biometry is essential to ensuring good clinical care. Most ultrasound quality control programs require expert sonographers to review a proportion of ultrasounds manually.[14,17,21] More recently, deep learning models have been developed to automate ultrasound quality control, however most of these tools are classifiers that detect optimal planes for fetal biometry. [22–24][25] Although tools that determine if biometry was captured in a standard plane is helpful and can help better a sonographer’s skills, they work under the assumption that a perfect ultrasound plane is necessary to make an appropriate GA estimate. Here, we provide an alternative quality control approach that checks the GA estimate directly instead of the biometry plane and only attempts to flag clinically meaningful errors. Our model allows for quality control of GA to be both accurate and flexible. As handheld ultrasound probes are increasingly used by novice users to compute biometry in low resource settings, this tool can help ensure increased accuracy in GA estimation.

Strengths of this study include prospective data collection and large, multi-country sample. Although our tool is intended for low-resource settings, we include data from both UNC Chapel Hill and Zambia to increase heterogeneity and generalizability. Additionally, each frame in our fly-to model is used during training of the neural network, including many 2D stills that do not capture fetal biometry in the perfect frame. This gives our model the flexibility to make an accurate estimate even in the face of substandard image acquisition (i.e., truncated fly-to cineloop). One limitation of our study includes the use of simulated novice biometry. We recognize that adding uniform absolute error of on average 5%, 7.5%, or 10% is simply a literature-based[6,15–17] estimation of what might be found among novice sonographers in real practice. We also acknowledge that the choice of sensitivity-specificity trade off may be context dependent. For example, in a very rural setting where there is no opportunity for follow-up, one might select a cut-point along the ROC curve with higher sensitivity, even though this would mean more scans were rejected. Additionally, future investigation of our tool with both novice and expert sonographers would be required to field test the tool’s utility in real practice. Real-world data would allow us to better evaluate the tool and understand how to fine-tune it depending on setting of use.

### Conclusion

In conclusion, this study introduces a deep learning model that leverages easily obtained fly-to cineloops for improved gestational age estimation. The model demonstrates superior performance to traditional biometry methods, particularly in the third trimester. As such, it could potentially be integrated into low-cost ultrasound devices to prevent the reporting of gestational age estimates that diverge significantly from acceptable quality thresholds. This tool represents an innovative approach to quality control in ultrasound, which is especially vital in low-resource settings where obstetric sonography is being introduced to novice users. The context-specific balance between sensitivity and specificity will be a crucial factor in practical application. Further field testing is necessary to assess the real-world effectiveness and applicability of this approach, paving the way for future improvements in maternal and fetal health in these critical environments.

## Supplementary Material

Supplementary Appendix

## Figures and Tables

**Table 1:** Characteristics of participants

	Training and Tuning Sets		Test Set	
	North Carolina (n = 797)	Zambia (n = 3432)	North Carolina (n = 414)	Zambia (n = 644)
Age	30.6 (26.3,33.8)	27.6 (23.6,32.2)	30.6 (27.1,33.9)	27.9 (23.8,32.6)
Body mass index (kg/m^2^)^a^	25.1 (22.1, 29.4)	24.4 (21.8, 28.2)	25.2 (22.4, 29.1)	24.6 (22.0, 27.9)
Parity	2 (1, 3)	3 (2, 4)	2 (1, 3)	3 (2, 4)
GA at dating ultrasound^b^	9.7 (8.0, 11.6)	25.1 (19.8, 30.7)	9.7 (8.0, 12.1)	22.8 (17.9, 26.9)
Number of studies^c^	2 (1, 11)	1 (1, 6)	2 (1, 10)	1 (1, 5)
Chronic hypertension^d^	25 (3.1)	209 (6.1)	13 (3.1)	39 (6.1)
Diabetes^e^	45 (5.6)	18 (0.5)	22 (5.3)	3 (0.5)
HIV infection	3 (0.4)	842 (24.5)	0 (0.0)	218 (33.9)
Syphilis in pregnancy	4 (0.5)	100 (2.9)	1 (0.2)	22 (3.4)
Alcohol during pregnancy	156 (19.6)	309 (9.0)	88 (21.3)	74 (11.5)
IVF Conception	20 (2.5)	0 (0.0)	18 (4.3)	0 (0.0)

Results presented as median [IQR] or n (%) unless otherwise stated

a The body-mass index (BMI) is the weight in kilograms divided by the square of the height in meters. Missing height prevents BMI calculation of 404 calculations overall.

b GA at dating ultrasound in weeks for IVF patients not applicable as GA assigned by embryo transfer

c Number of studies presented as median [range]

d Chronic hypertension was defined as diagnosis of hypertension before pregnancy or new diagnosis in first 20 weeks of pregnancy.

e Diabetes was defined as both pregestational and gestational diabetes

**Table 2:** Gestational age estimation of deep learning model compared to trained sonographer in test set

	Test Set (n = 1,058)^a^		
	Model	Biometry	Difference (95% CI)
Mean Absolute Error (SE), days	3.87 (0.07)	4.80 (0.10)	−0.92 (−1.10, −0.76)
Root Mean Square Error (SE), days	5.01 (0.11)	6.47 (0.14)	−1.46 (−1.70, −1.21)
2^nd^ trimester^b^ Mean Absolute Error (SE), days	3.14 (0.09)	3.28 (0.10)	−0.14 (−0.31, 0.02)
3^rd^ trimester^c^ Mean Absolute Error (SE), days	4.44 (0.10)	5.98 (0.14)	−1.53 (−1.80, −1.26)
Absolute Error < 10 days (SE), %	95.0 (0.5)	88.3 (0.7)	6.7 (5.3, 8.2)
UNC MAE, days	3.81 (0.10)	4.05 (0.12)	−0.24 (−0.46, 0.03)
Zambia MAE, days	3.93 (0.10)	5.11 (0.15)	−1.58 (−1.84, −1.32)

a Test set as described in Figure 2

b 2^nd^ trimester is defined as 98 to 185 days

c 3^rd^ trimester is defined as 186 days to 280 days

**Table 3:** Deep learning model’s performance to detect 10-day discrepancy between novice^a^ and expert sonographer

Tuning Set (n = 846)^b^		Test Set (n = 1,058)^b^			
Sensitivity in tuning set	Threshold based on tuning set (days)*	Sensitivity	Specificity	Positive predictive value	Negative predictive value
80%	10.6	81.0 (77.9, 83.8)	89.9 (88.1, 91.5)	81.6 (78.6, 84.4)	89.5 (87.7, 91.1)
90%	8.5	89.4 (87.0, 91.6)	80.8 (78.6, 82.9)	72.1 (69.0, 75.0)	93.2 (91.6, 94.6)
95%	6.2	96.2 (94.6, 97.5)	65.0 (62.4, 67.6)	60.4 (57.5, 63.2)	96.9 (95.5, 98.0)

a with 7.5% novice biometry absolute error

b Tuning set and test set as described in Figure 2

**Table 4:** Two-way sensitivity analyses investigating performance of deep learning model to detect gross error over a variety of scenarios, expressed as area under the receiver operating characteristics (ROC) curve

(a) AUC when model is provided full-length fly-to cineloops				
		Novice Biometry Absolute Error		
**Definition of Gross Error**		5%	7.5%	10%
	±7 days	0.87	0.9	0.92
	±10 days	0.89	0.93^a^	0.94
	±14 days	0.89	0.95	0.96
(b) AUC when model is provided truncated fly-to cineloops				
		Novice Biometry Absolute Error		
**Definition of Gross Error**		5%	7.5%	10%
	±7 days	0.86	0.89	0.91
	±10 days	0.87	0.92	0.93
	±14 days	0.88	0.93	0.95

Columns depict variation in the amount of random error introduced to simulate novice biometry (7.5% is the base-case assumption). Rows depict variation in our definition of gross error, i.e., the magnitude of discrepancy between novice and expert sonography that would be flagged by the model (±10 days is the base-case assumption). The two-way sensitivity analysis is conducted with the model being provided full length fly-to cineloops (section a) and truncated fly-to cineloops (section b). AUC = area under the ROC Curve

a Base case

## Synopsis:

Our model predicts GA more accurately from routinely available fly-to cineloops than expert sonographer acquired biometry and can act as a guardrail to prevent gross GA estimation errors.

## References

[1] WHO recommendations on antenatal care for a positive pregnancy experience. https://www.who.int/publications/i/item/9789241549912. Accessed 2023 Mar 30. 28079998

[2] Whitworth M, Bricker L, Mullan C. Ultrasound for fetal assessment in early pregnancy. Cochrane Database Syst Rev. 2015. 10.1002/14651858.CD007058.pub3 PMC6464767 26171896

[3] Becker DM, Tafoya CA, Becker SL, Kruger GH, Tafoya MJ, Becker TK. The use of portable ultrasound devices in low- and middle-income countries: a systematic review of the literature. Trop Med Int Health. 2016;21(3):294–311. 26683523 10.1111/tmi.12657

[4] Lamprecht H, Lemke G, van Hoving D, Kruger T, Wallis L. Poor return on investment: investigating the barriers that cause low credentialing yields in a resource-limited clinical ultrasound training programme. Int J Emerg Med. 2018;11(1). 10.1186/s12245-018-0168-9 PMC5821624 29468453

[5] Ginsburg AS, Liddy Z, Khazaneh PT, May S, Pervaiz F. A survey of barriers and facilitators to ultrasound use in low- and middle-income countries. Scientific Reports. 2023;13(1):1–11. 36849625 10.1038/s41598-023-30454-w PMC9969046

[6] Rijken MJ, Lee SJ, Boel ME, Papageorghiou AT, Visser GHA, Dwell SLM, Obstetric ultrasound scanning by local health workers in a refugee camp on the Thai–Burmese border. Ultrasound Obstet Gynecol. 2009;34(4):395–403. 19790099 10.1002/uog.7350 PMC3438883

[7] Nathan RO, Swanson JO, Swanson DL, McClure EM, Bolamba VL, Lokangaka A, Evaluation of Focused Obstetric Ultrasound Examinations by Health Care Personnel in the Democratic Republic of Congo, Guatemala, Kenya, Pakistan, and Zambia. Curr Probl Diagn Radiol. 2017;46(3):210–5. 28057388 10.1067/j.cpradiol.2016.11.001 PMC5413583

[8] Pokaprakarn T, Prieto JC, Price JT, Kasaro MP, Sindano N, Shah HR, AI Estimation of Gestational Age from Blind Ultrasound Sweeps in Low-Resource Settings. NEJM Evidence. 2022;1(5). 10.1056/evidoa2100058 PMC9980216 36875289

[9] Price JT, Winston J, Vwalika B, Cole SR, Stoner MCD, Lubeya MK, Quantifying bias between reported last menstrual period and ultrasonography estimates of gestational age in Lusaka, Zambia. Int J Gynecol Obstet. 2019;144(1):9–15. 10.1002/ijgo.12686 PMC6283668 30267538

[10] Papageorghiou AT, Kemp B, Stones W, Ohuma EO, Kennedy SH, Purwar M, Ultrasound-based gestational-age estimation in late pregnancy. Ultrasound Obstet Gynecol. 2016;48(6):719–26. 26924421 10.1002/uog.15894 PMC6680349

[11] Pettker CM, Goldberg JD, El-Sayed YY, Copel JA. Committee Opinion No 700: Methods for Estimating the Due Date. Obstetrics and gynecology. 2017;129(5):E150–4. 28426621 10.1097/AOG.0000000000002046

[12] Hadlock FP, Deter RL, Harrist RB, Park SK. Estimating fetal age: computer-assisted analysis of multiple fetal growth parameters. Radiology. 1984;152(2):497–501. 6739822 10.1148/radiology.152.2.6739822

[13] R Core Team. R: A language and environment for statistical computing. Vienna, Austria: R Foundation for Statistical Computing; 2022.

[14] Salomon LJ, Alfirevic Z, Da Silva Costa F, Deter RL, Figueras F, Ghi T, ISUOG Practice Guidelines: ultrasound assessment of fetal biometry and growth. Ultrasound Obstet Gynecol. 2019;53(6):715–23. 31169958 10.1002/uog.20272

[15] Yang F, Leung KY, Lee YP, Chan HY, Tang MHY. Fetal biometry by an inexperienced operator using two- and three-dimensional ultrasound. Ultrasound Obstet Gynecol. 2010;35(5):566–71. 20183864 10.1002/uog.7600

[16] Neufeld LM, Wagatsuma Y, Hussain R, Begum M, Frongillo EA. Measurement error for ultrasound fetal biometry performed by paramedics in rural Bangladesh. Ultrasound Obstet Gynecol. 2009;34(4):387–94. 19504627 10.1002/uog.6385

[17] Cavallaro A, Ash ST, Napolitano R, Wanyonyi S, Ohuma EO, Molloholli M, Quality control of ultrasound for fetal biometry: results from the INTERGROWTH-21st Project. Ultrasound Obstet Gynecol. 2018;52(3):332–9. 28718938 10.1002/uog.18811

[18] Chama-Chiliba CM, Koch SF. Utilization of focused antenatal care in Zambia: examining individual- and community-level factors using a multilevel analysis. Health Policy Plan. 2015;30(1):78–87. 24357197 10.1093/heapol/czt099

[19] Chi BH, Vwalika B, Killam WP, Wamalume C, Giganti MJ, Mbewe R, Implementation of the Zambia Electronic Perinatal Record System for comprehensive prenatal and delivery care. Int J Gynecol Obstet. 2011;113(2):131. 10.1016/j.ijgo.2010.11.013 PMC3071887 21315347

[20] Butterfly Network Completes Successful Deployment of 500 Butterfly iQ+ Devices and Training of 500+ Healthcare Workers Across 224 Facilities in Kenya [Business Wire]. 2022 https://www.businesswire.com/news/home/20221219005090/en/Butterfly-Network-Completes-Successful-Deployment-of-500-Butterfly-iQ-Devices-and-Training-of-500-Healthcare-Workers-Across-224-Facilities-in-Kenya. Accesed 2023 May 29.

[21] Viner AC, Membe-Gadama G, Whyte S, Kayambo D, Masamba M, Makwakwa E, Training in Ultrasound to Determine Gestational Age (TUDA): Evaluation of a Novel Education Package to Teach Ultrasound-Naive Midwives Basic Obstetric Ultrasound in Malawi. Front Glob Womens Health. 2022;3:39. 10.3389/fgwh.2022.880615 PMC9017789 35449708

[22] Baumgartner CF, Kamnitsas K, Matthew J, Fletcher TP, Smith S, Koch LM, SonoNet: Real-Time Detection and Localisation of Fetal Standard Scan Planes in Freehand Ultrasound. IEEE Trans Med Imaging. 2017;36(11):2204–15. 28708546 10.1109/TMI.2017.2712367 PMC6051487

[23] Maraci MA, Bridge CP, Napolitano R, Papageorghiou A, Noble JA. A framework for analysis of linear ultrasound videos to detect fetal presentation and heartbeat. Med Image Anal. 2017;37:22–36. 28104551 10.1016/j.media.2017.01.003

[24] Ryou H, Yaqub M, Cavallaro A, Papageorghiou AT, Alison Noble J. Automated 3D ultrasound image analysis for first trimester assessment of fetal health. Phys Med Biol. 2019;64(18). 10.1088/1361-6560/ab3ad1 31408850

[25] Cheng PM, Malhi HS. Transfer Learning with Convolutional Neural Networks for Classification of Abdominal Ultrasound Images. J Digit Imaging. 2017;30(2):234–43. 27896451 10.1007/s10278-016-9929-2 PMC5359213

