# Supplementary material for: Deep learning to estimate gestational age from fly-to cineloop videos: a novel approach to ultrasound quality control
Source: Int J Gynaecol Obstet. Author manuscript; Available in PMC 2024 Jun 29. (PMC11214162; doi:10.1002/ijgo.15321)
Supplement: Supplementary Appendix [file NIHMS1991395-supplement-Supplementary_Appendix.docx]

# **Supplementary Figures**

**Figure S1: Normal vs truncated fly-to cineloops**


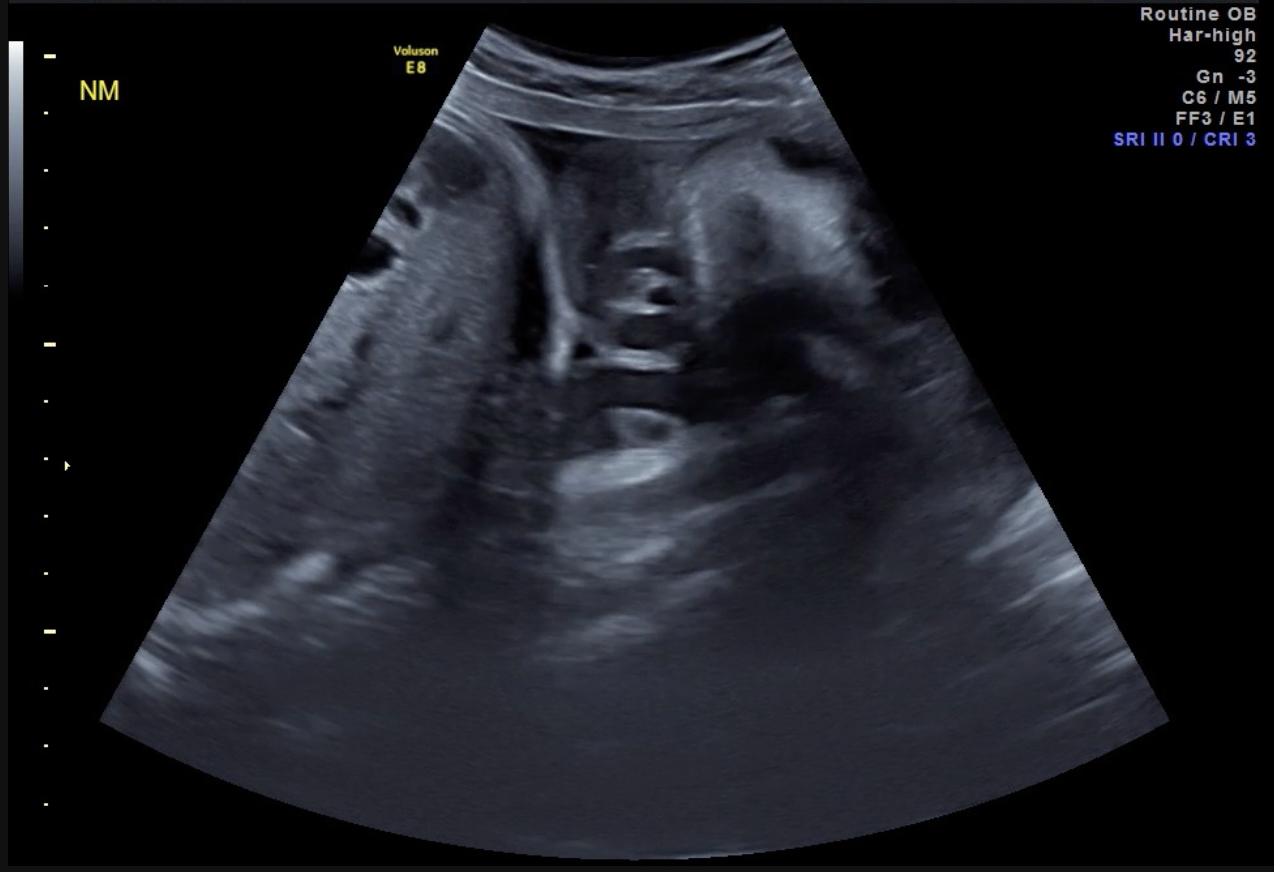

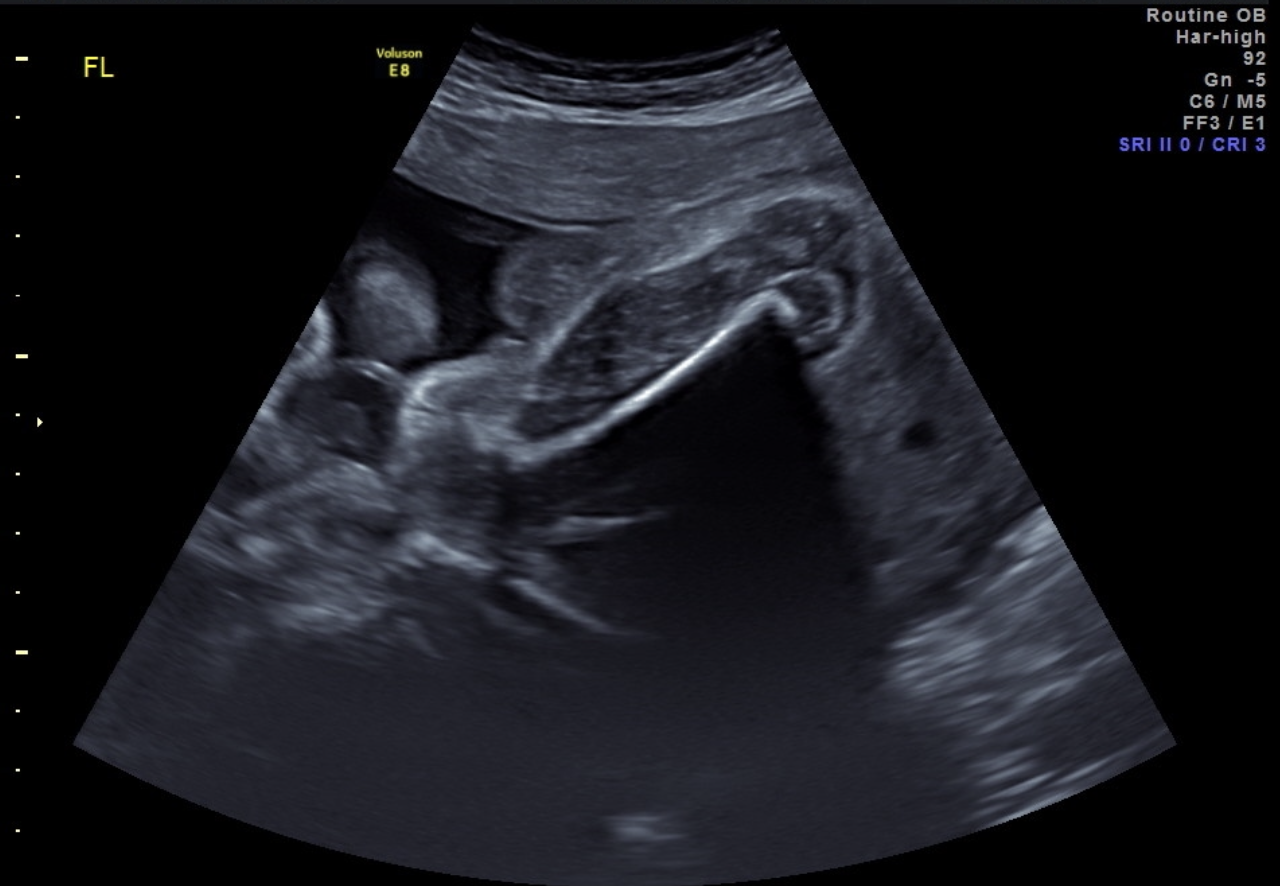

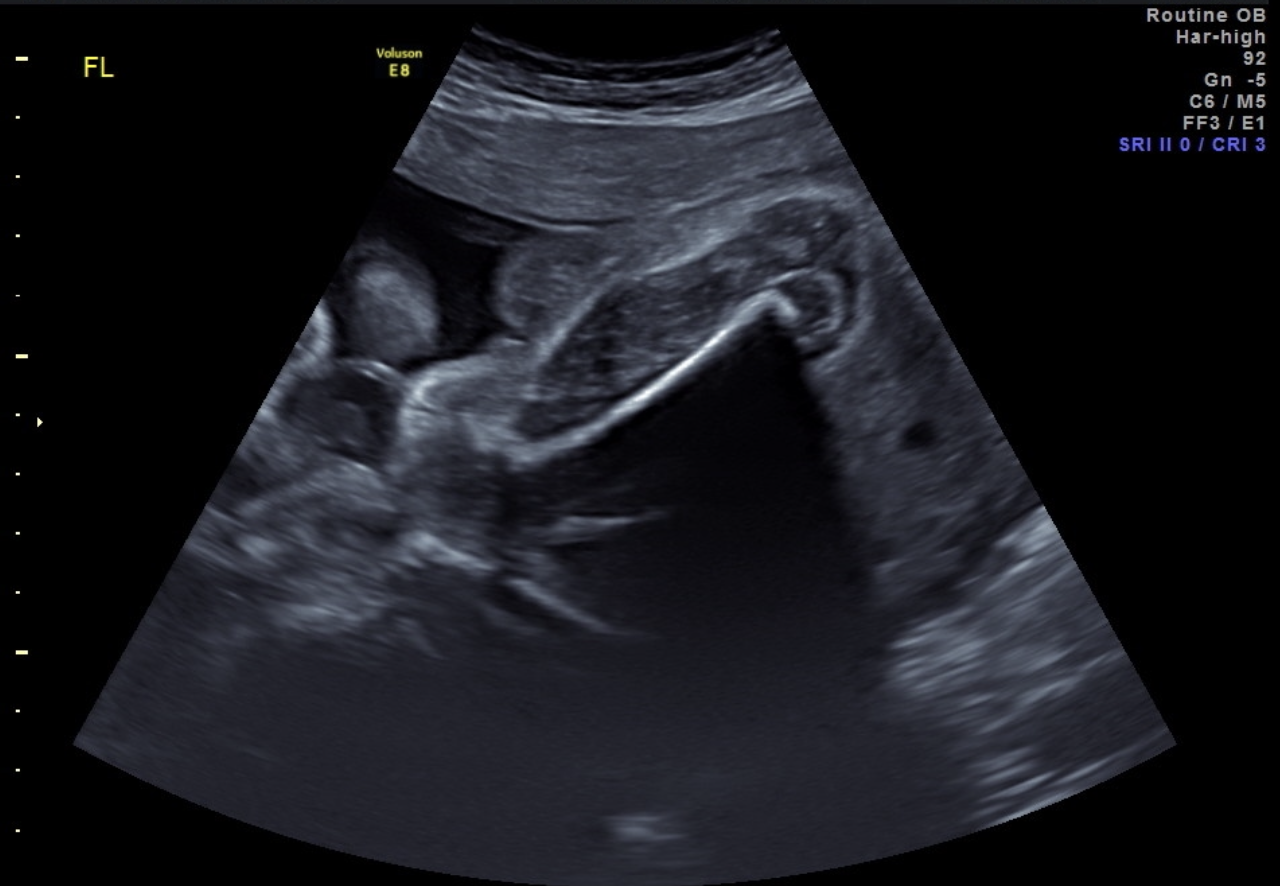

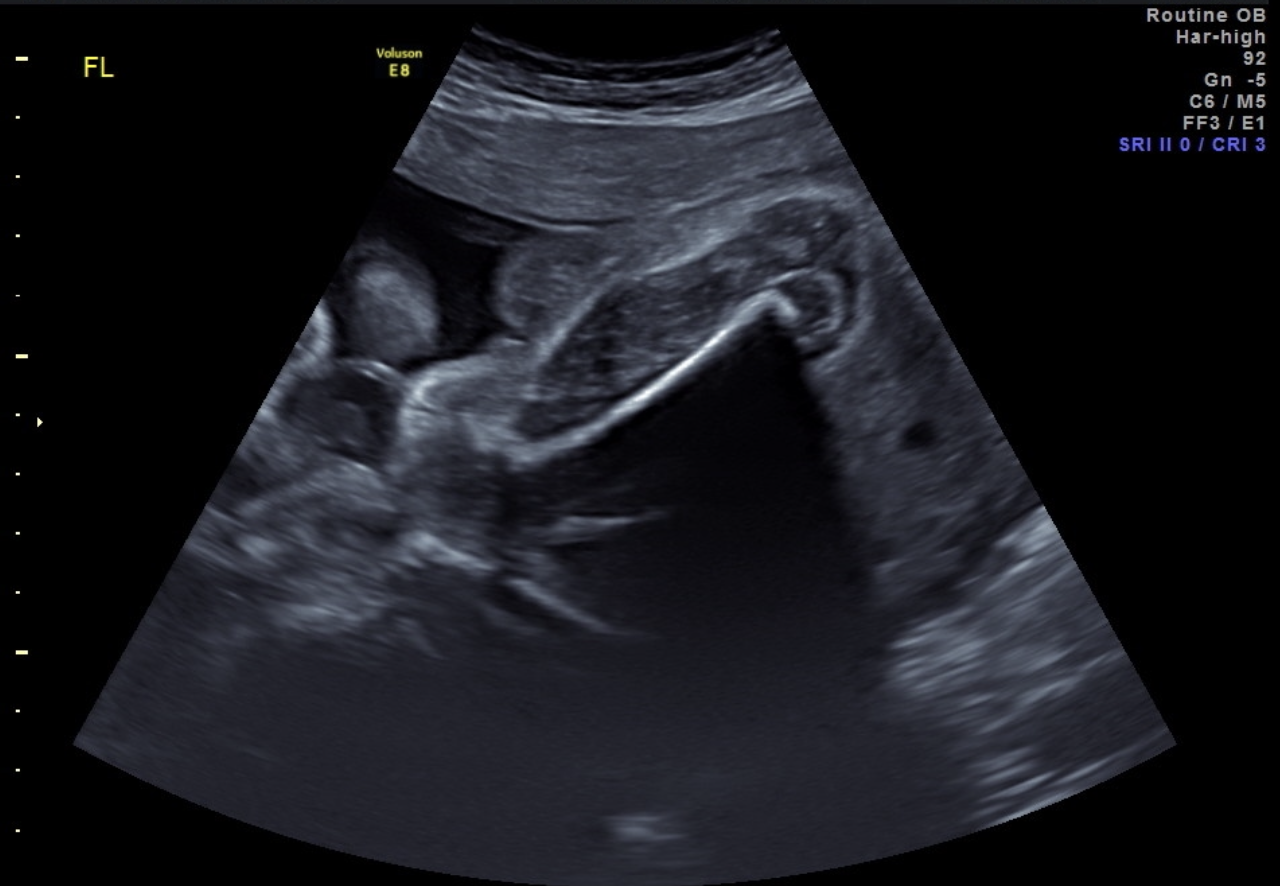

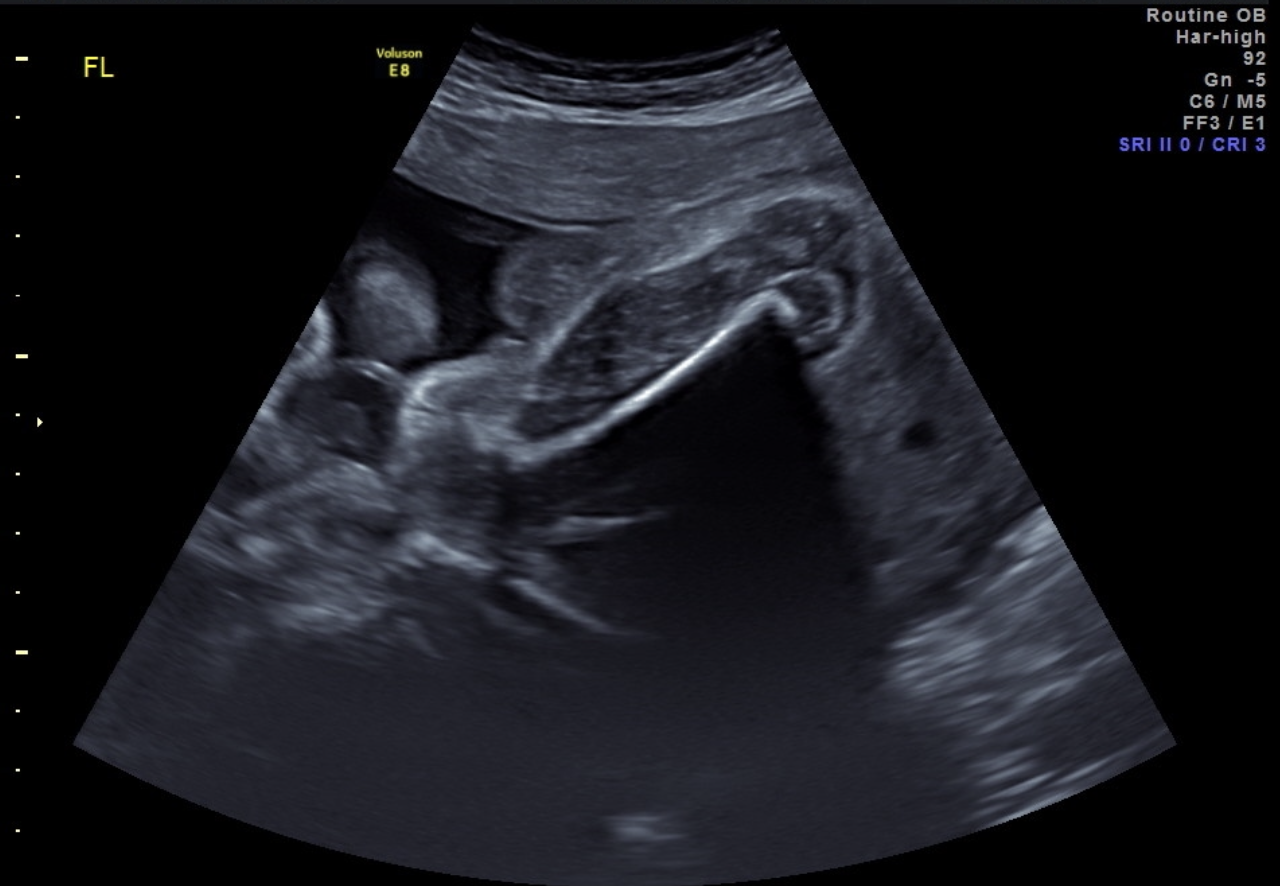

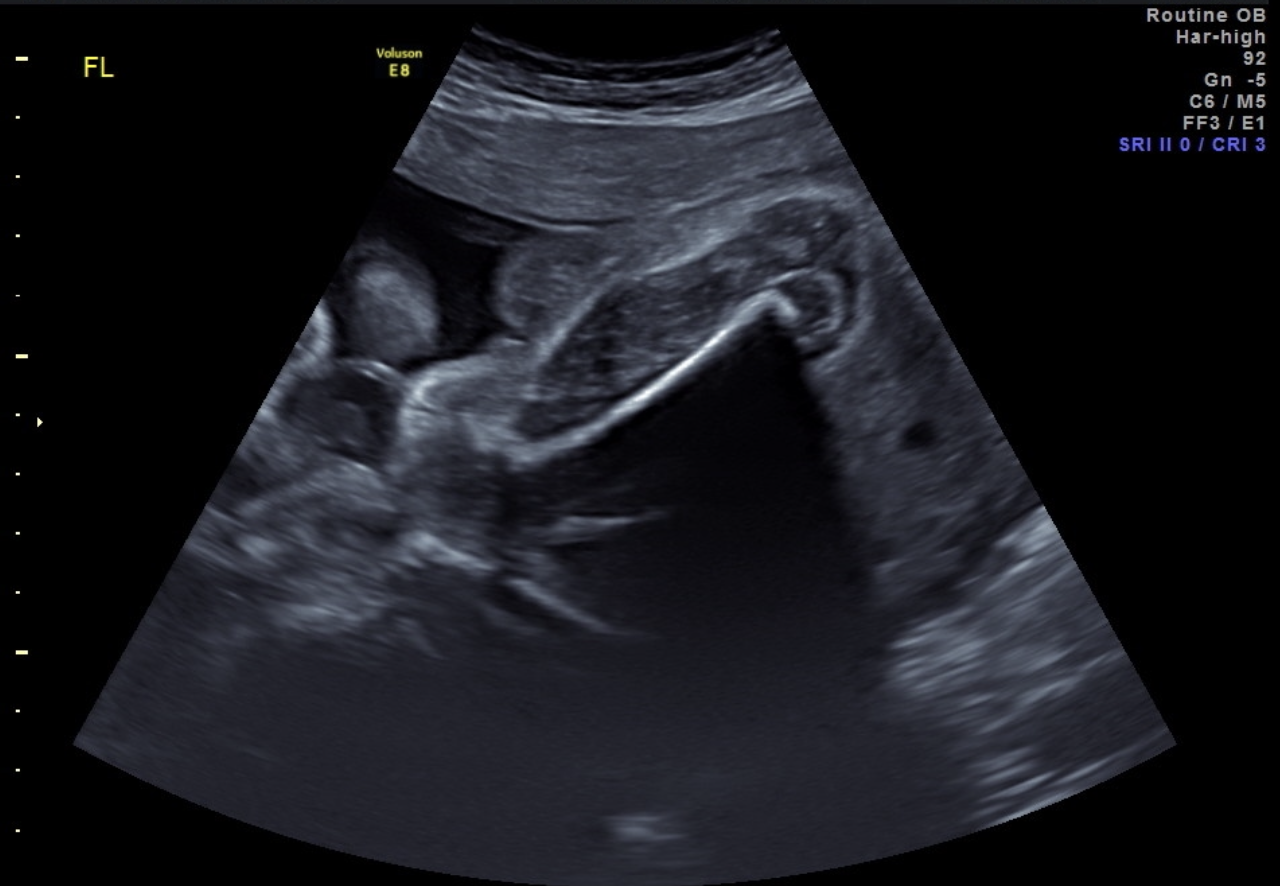

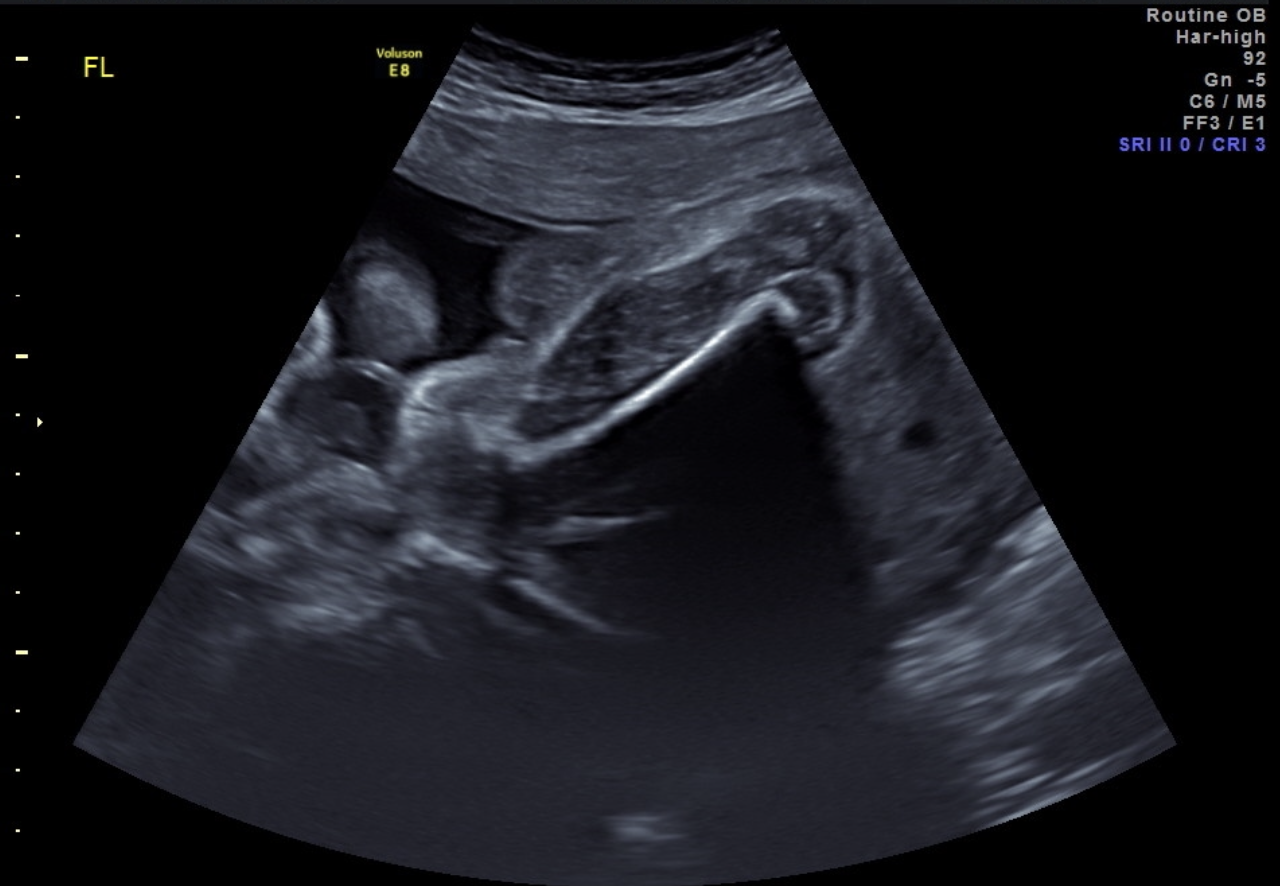

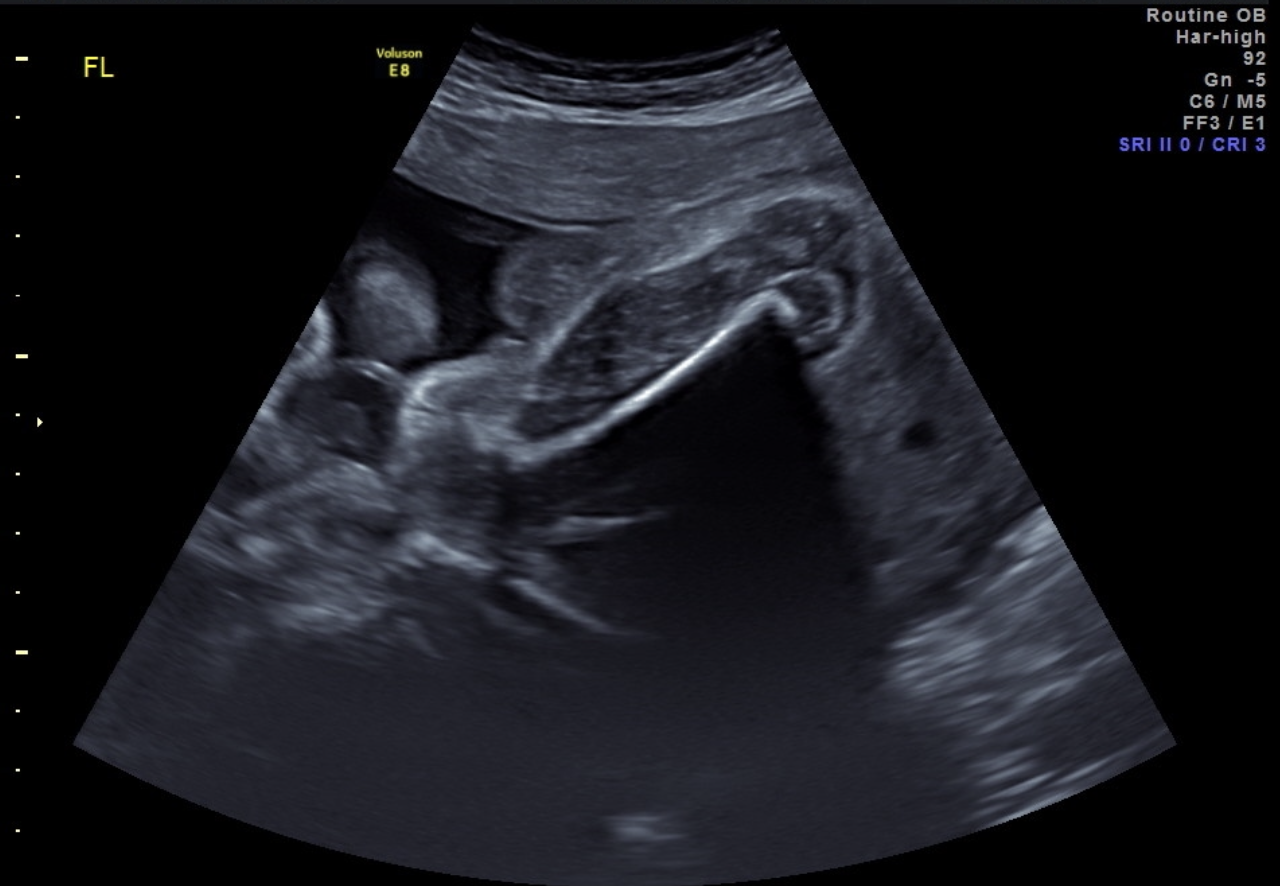

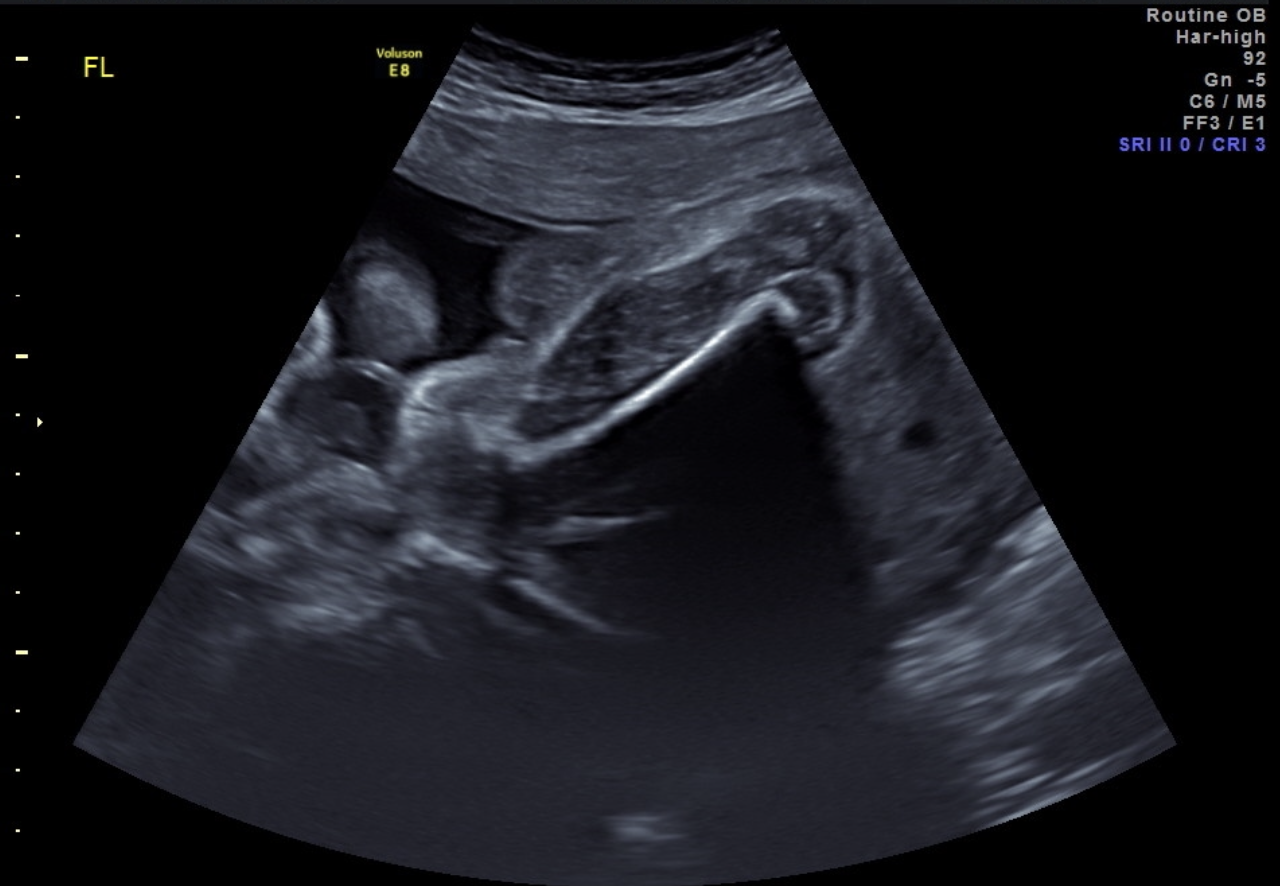

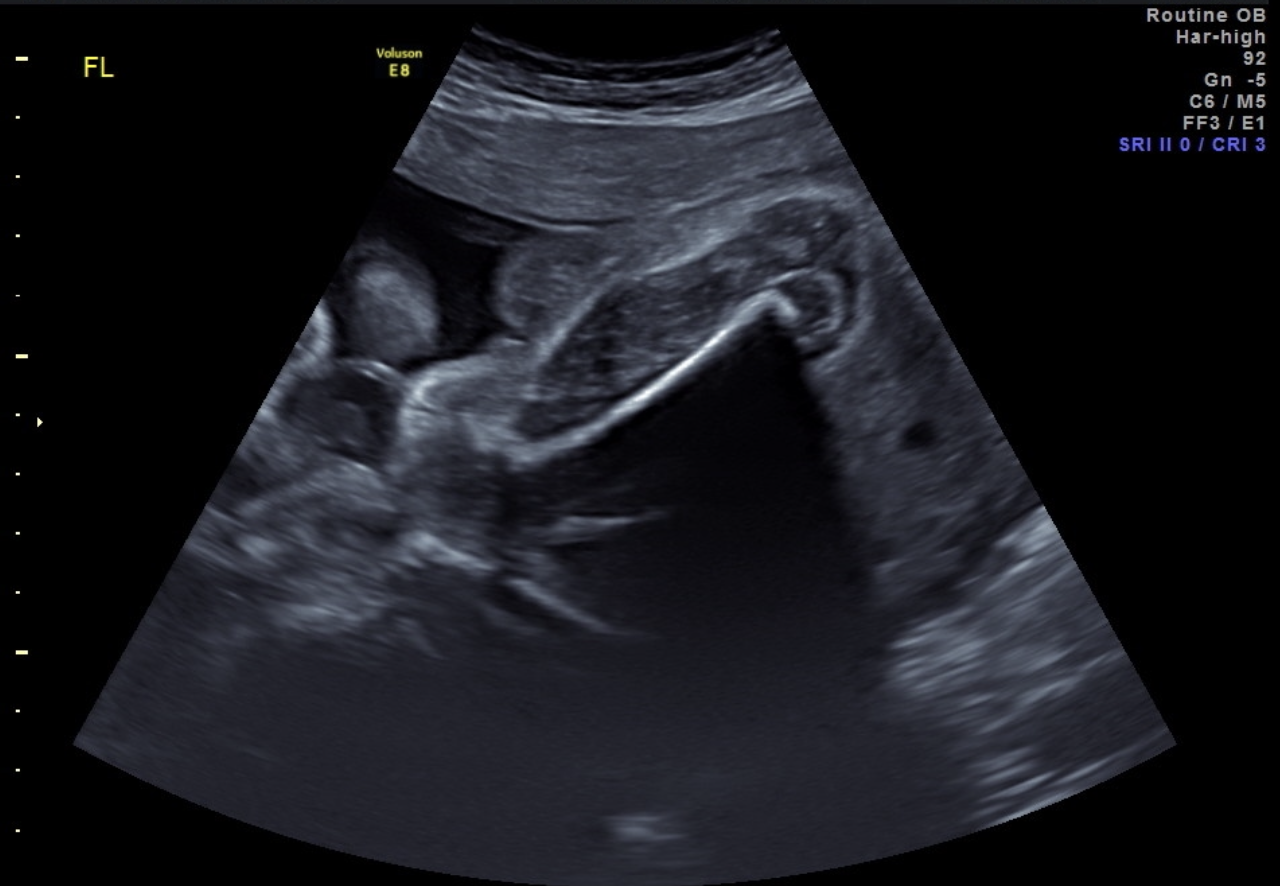

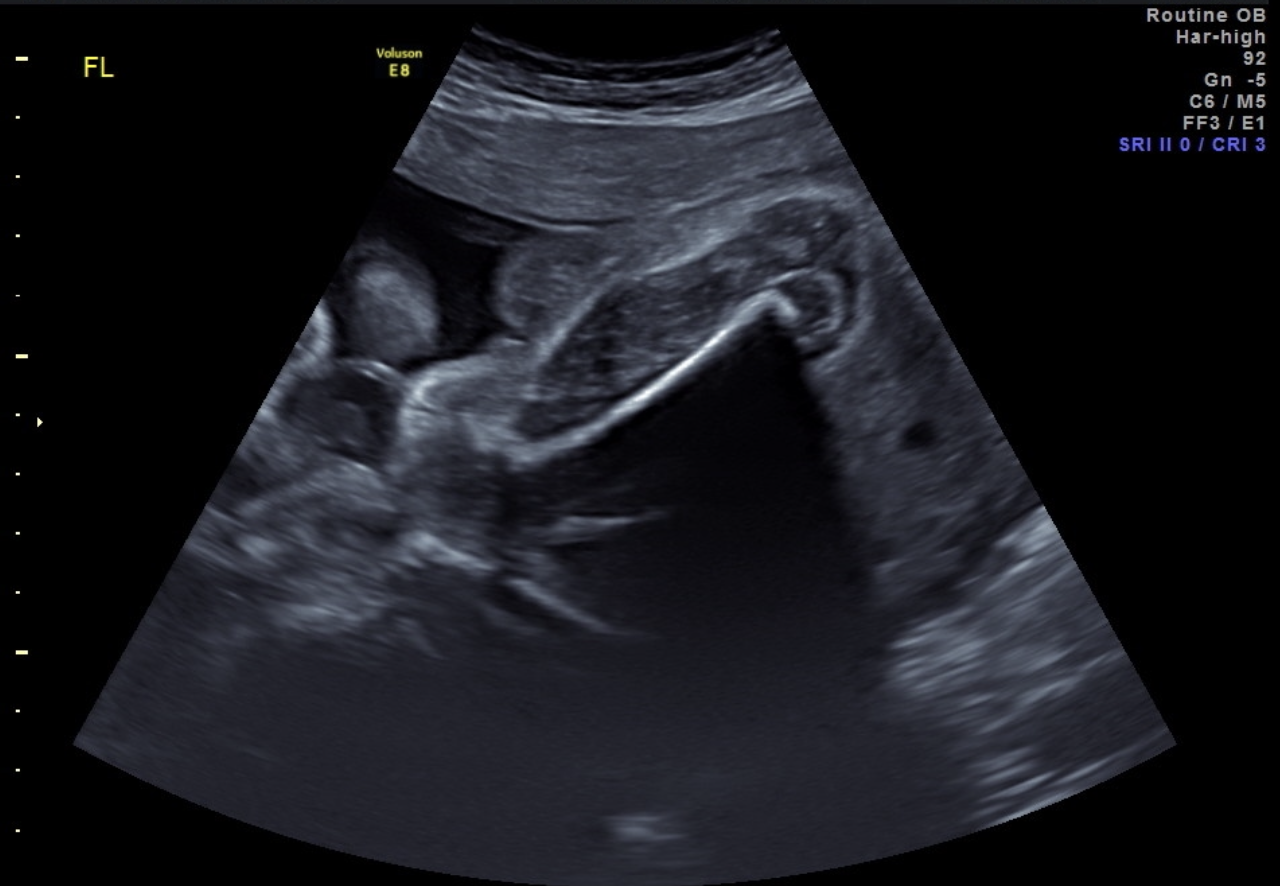

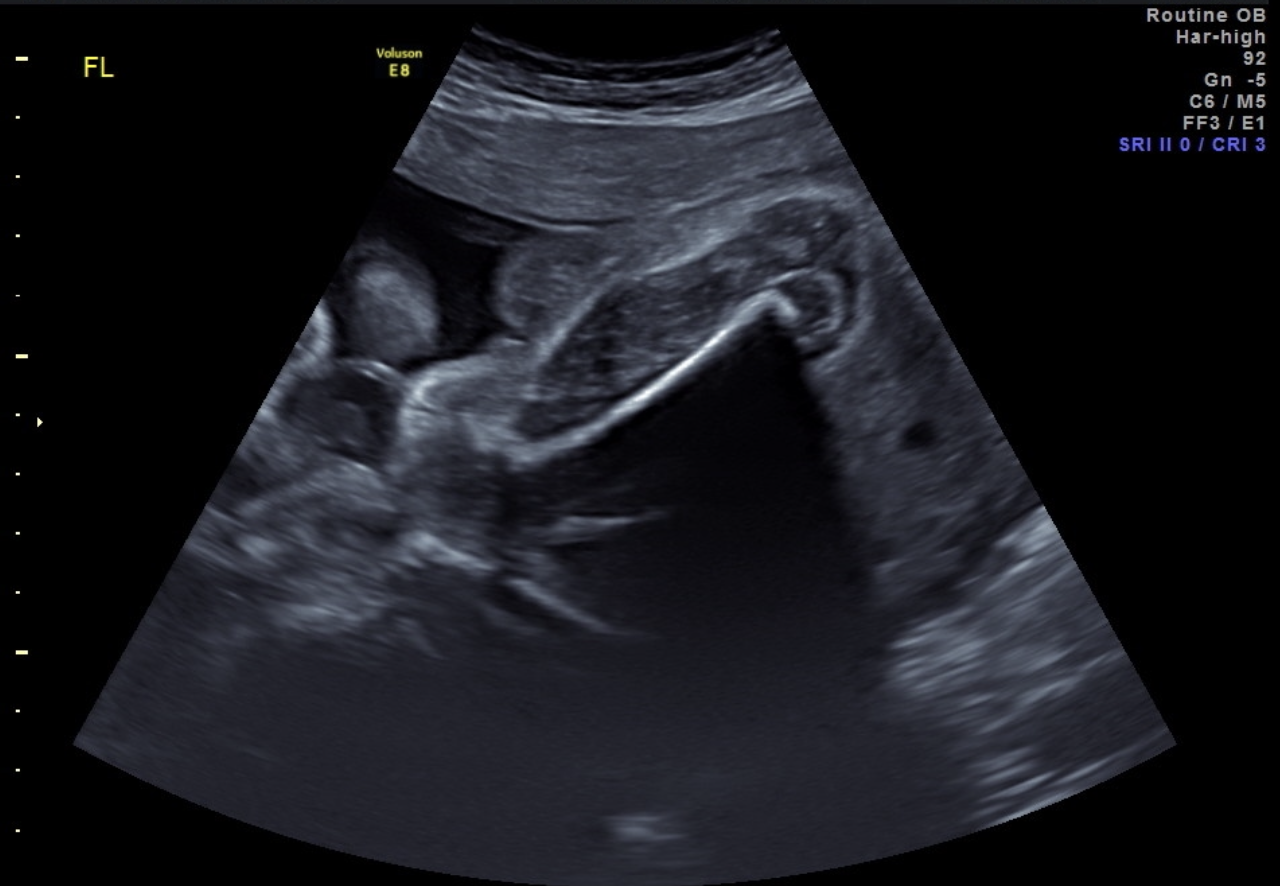

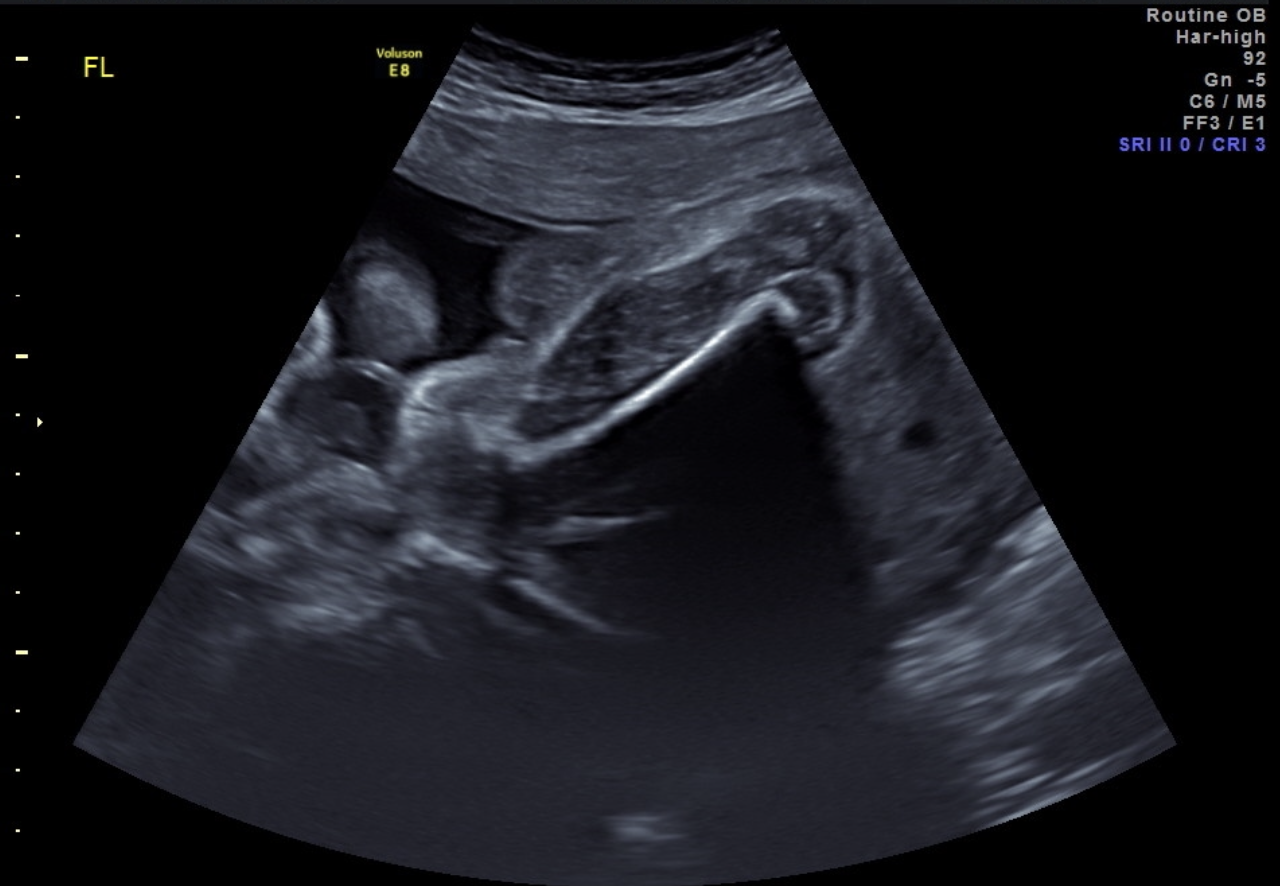

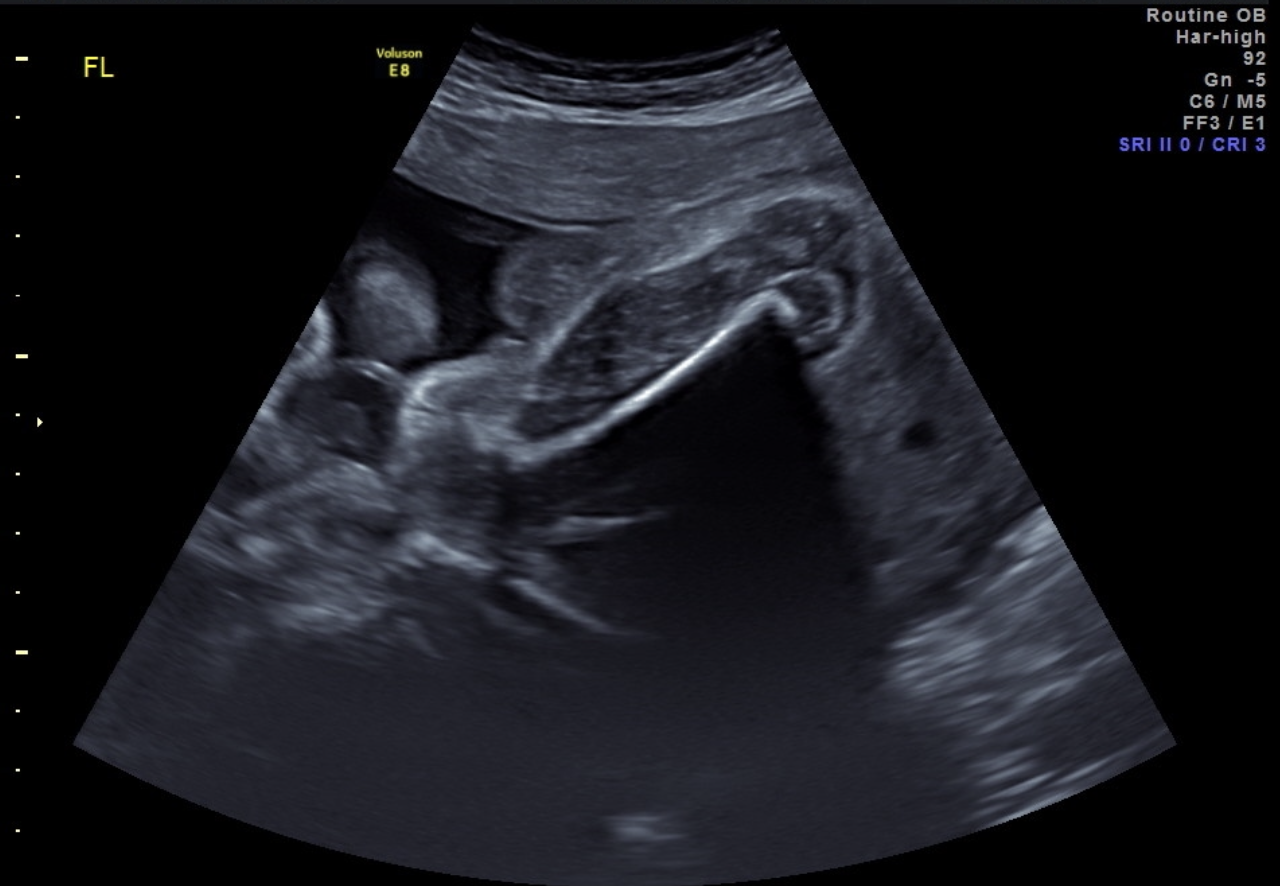

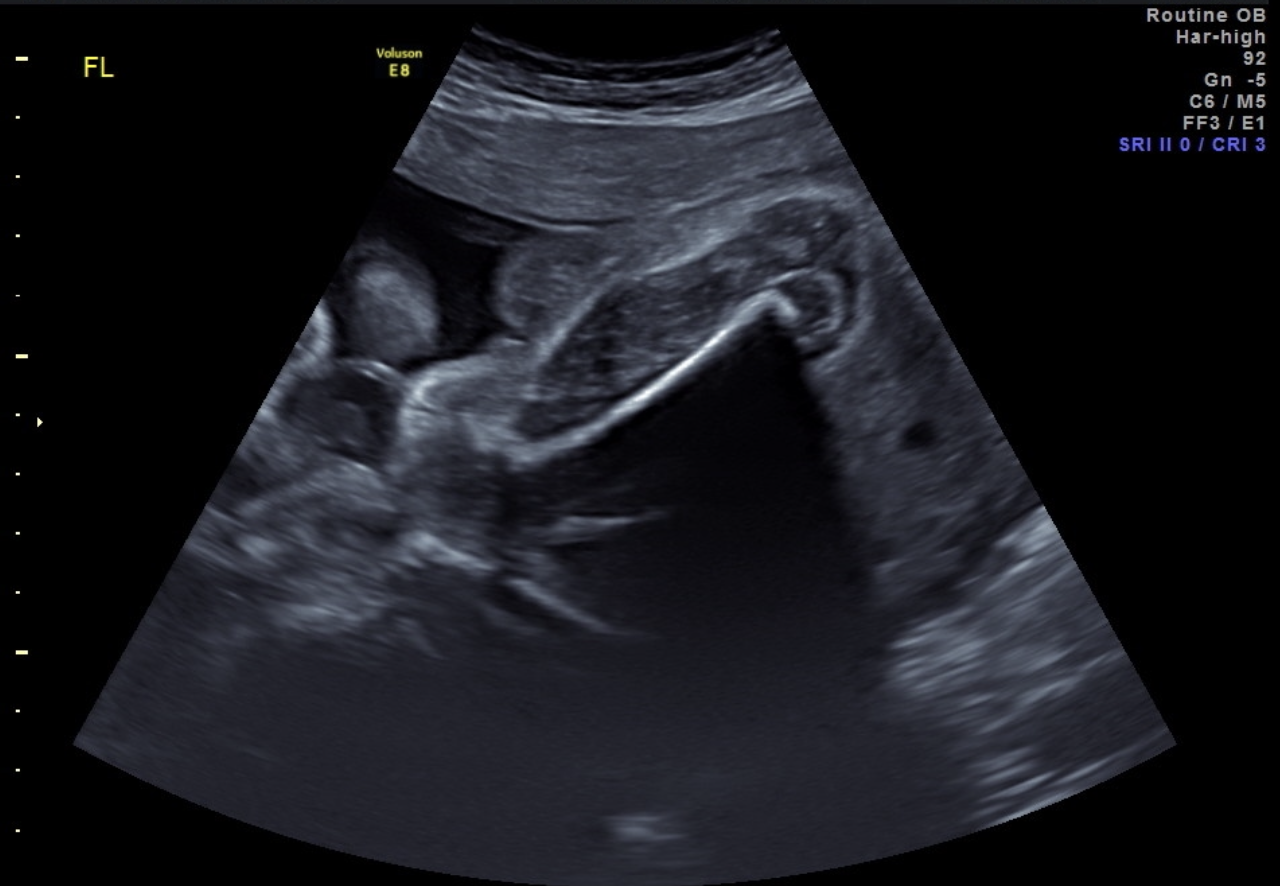

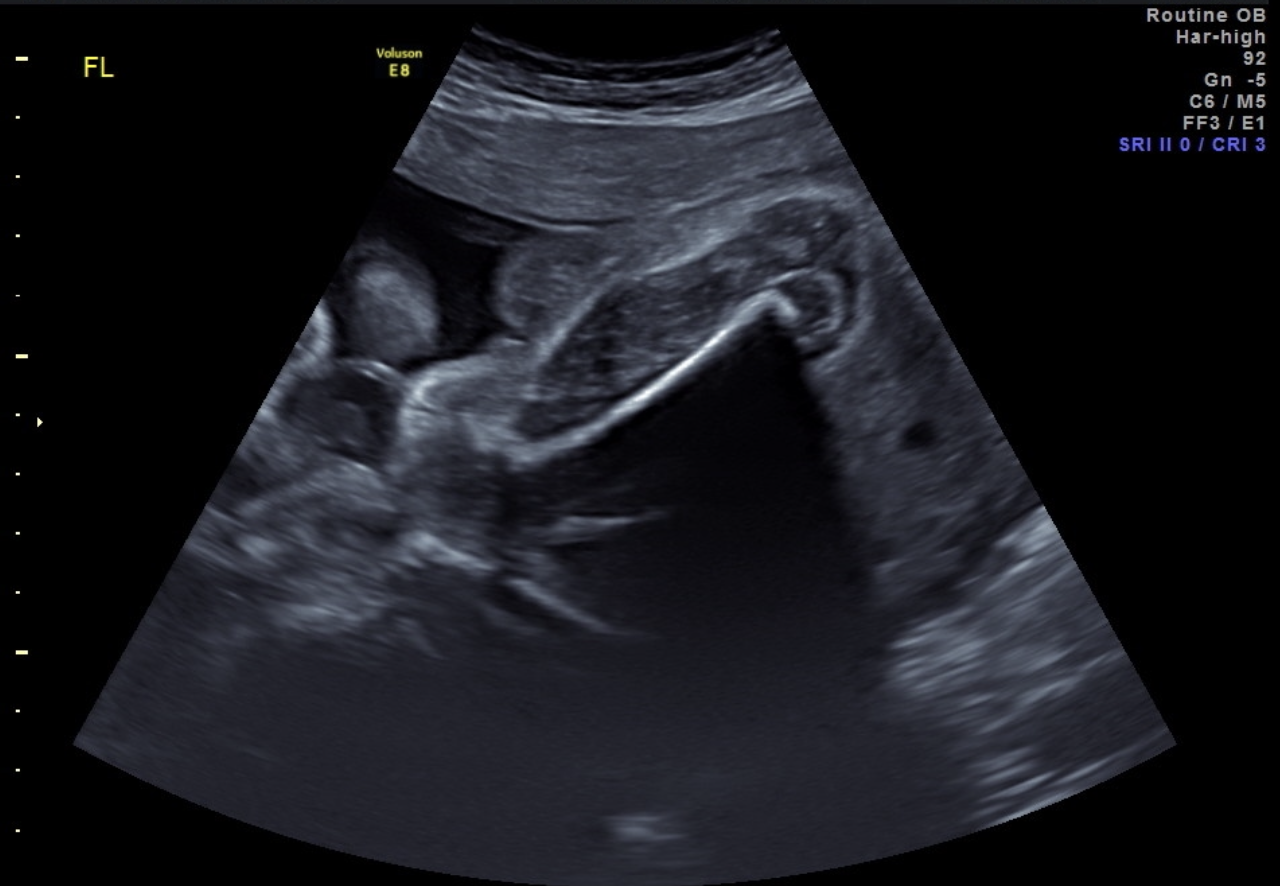

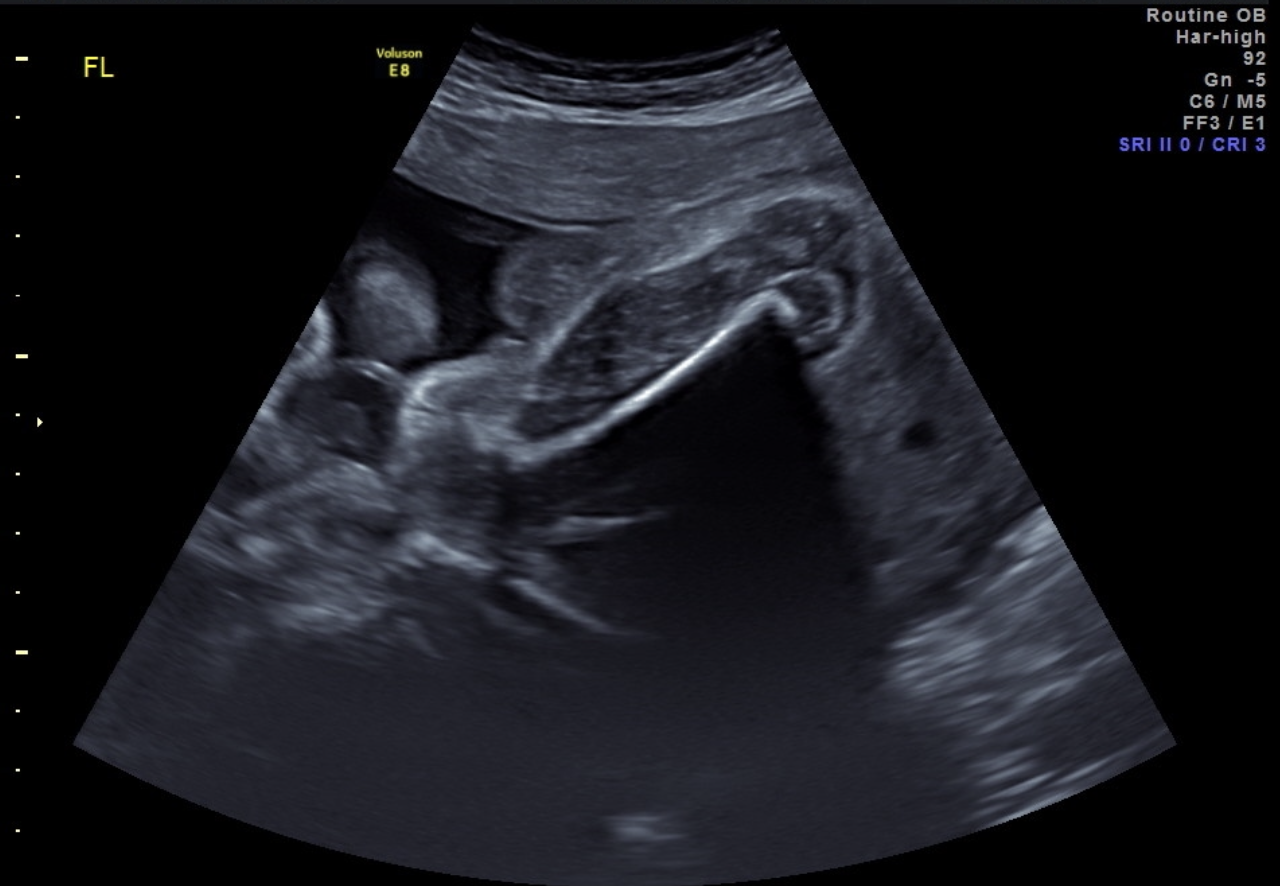

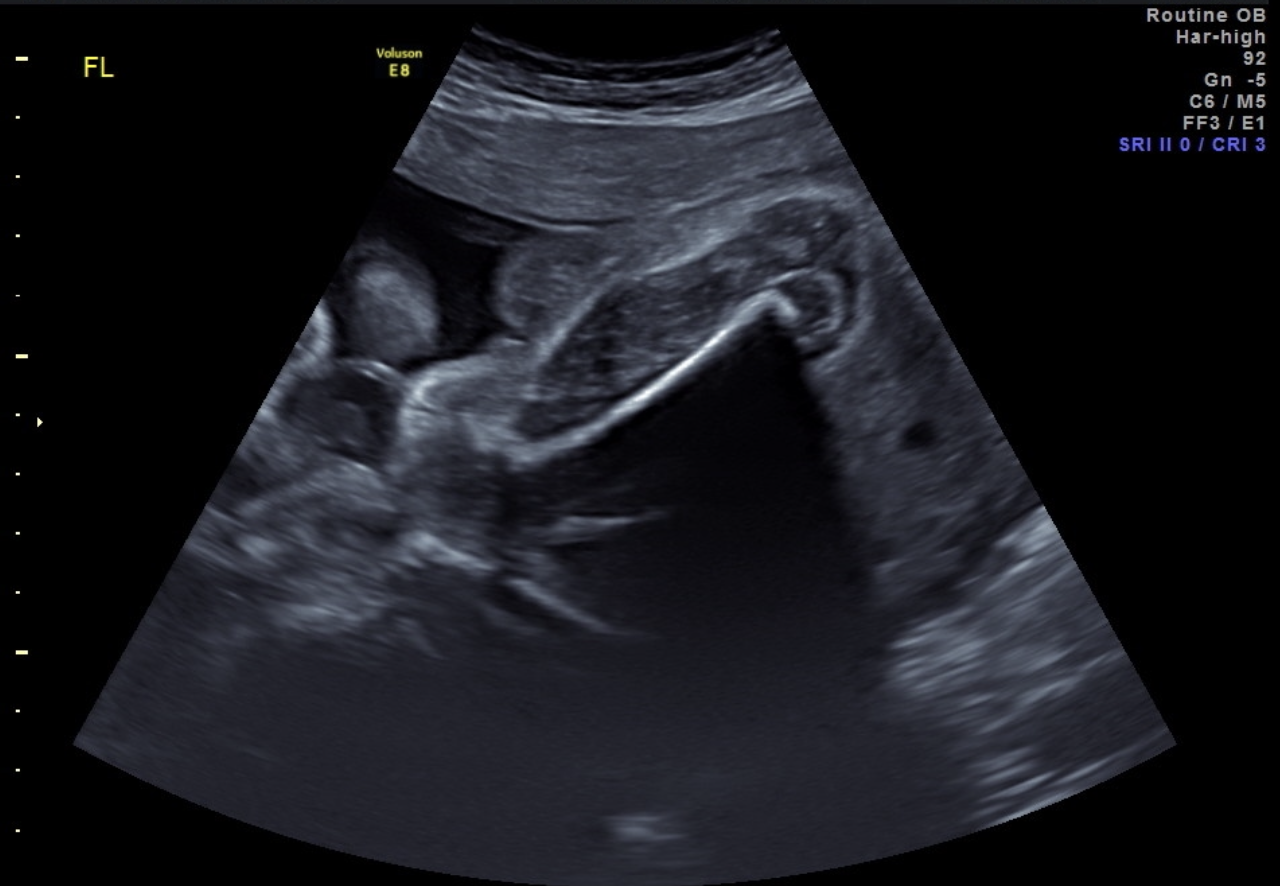

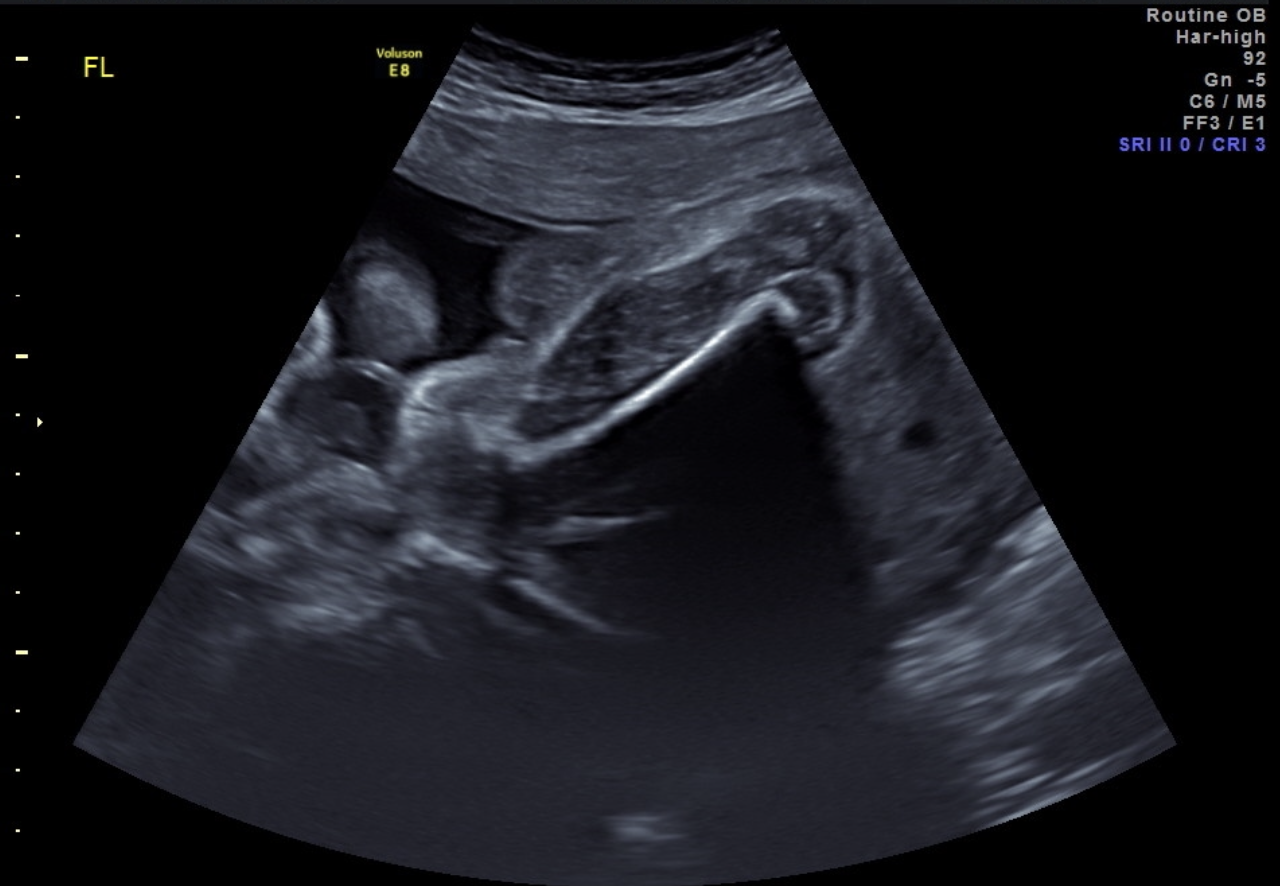

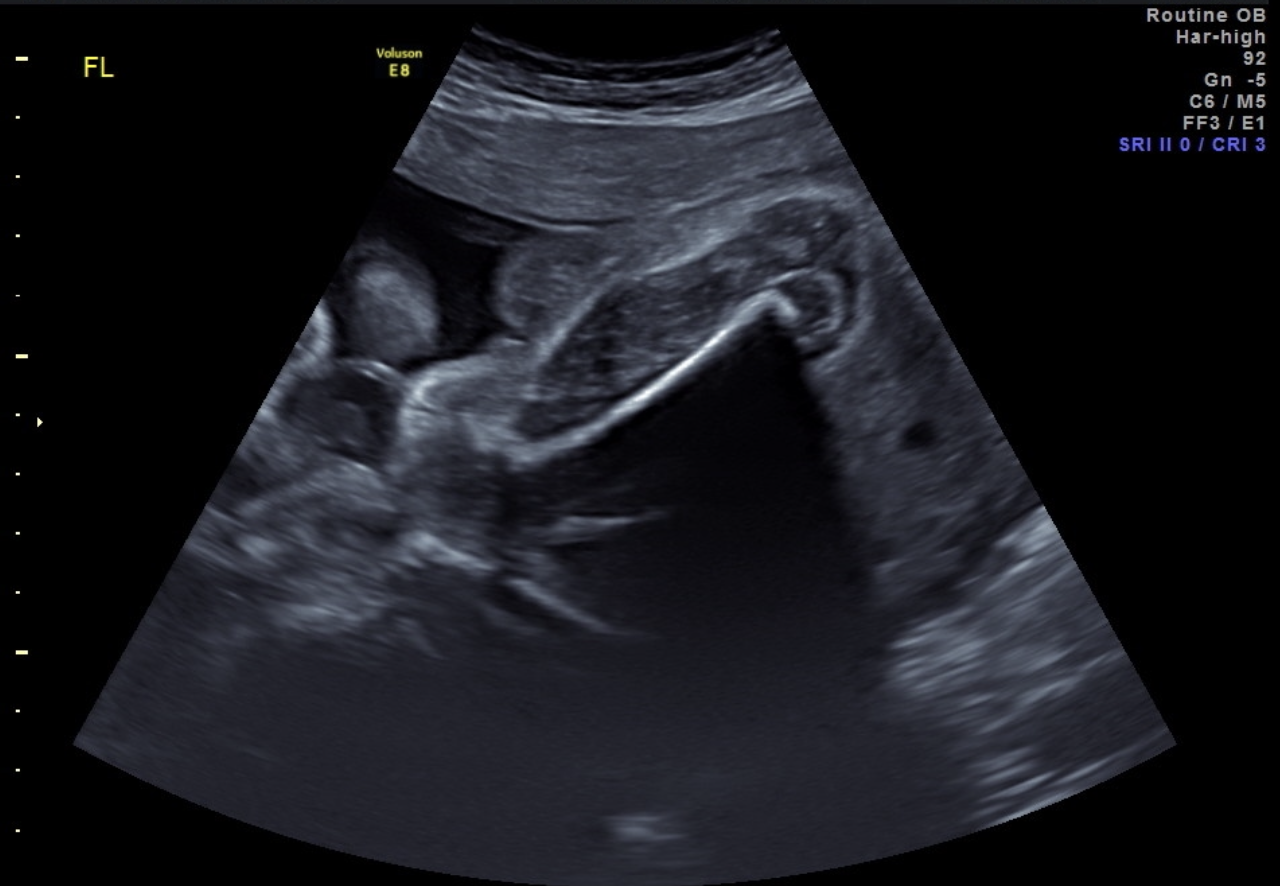

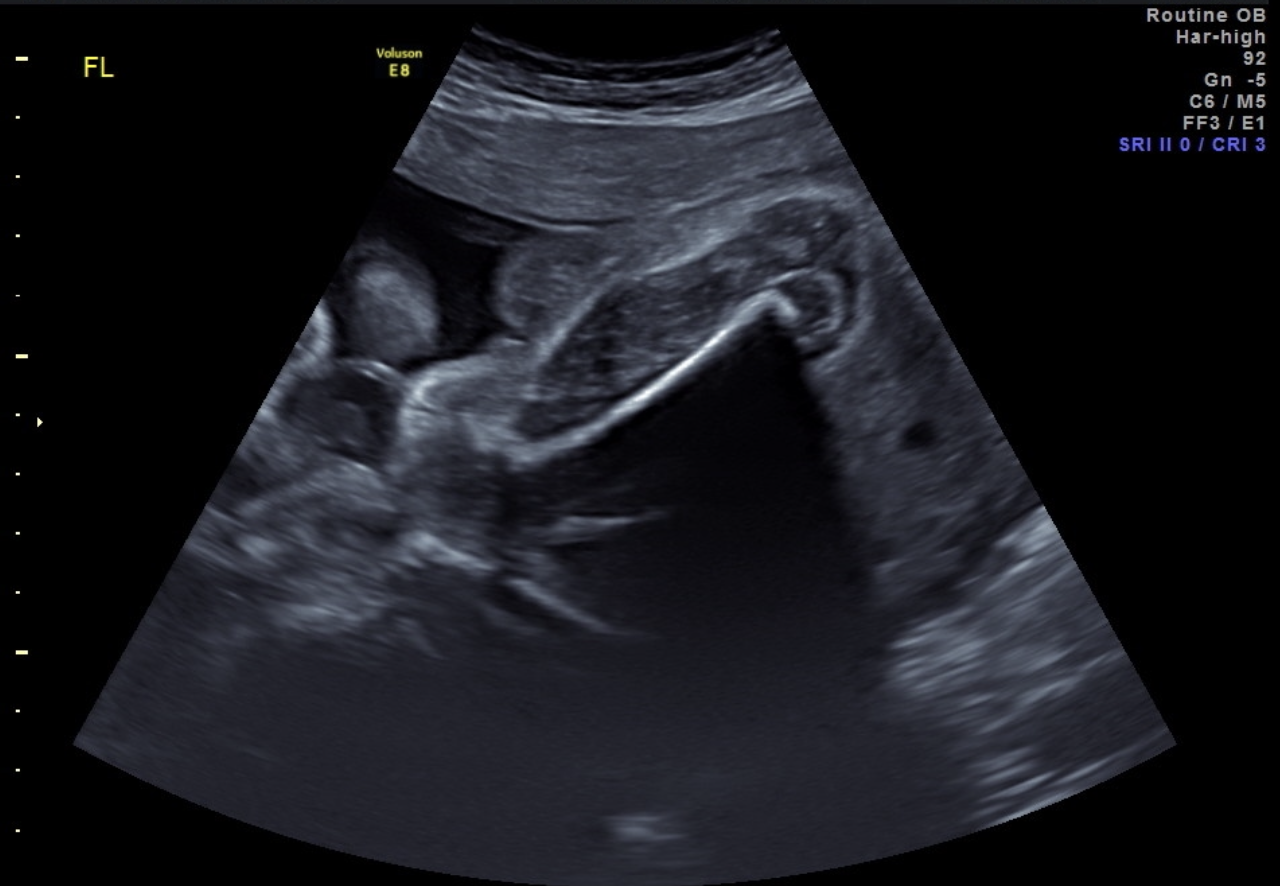

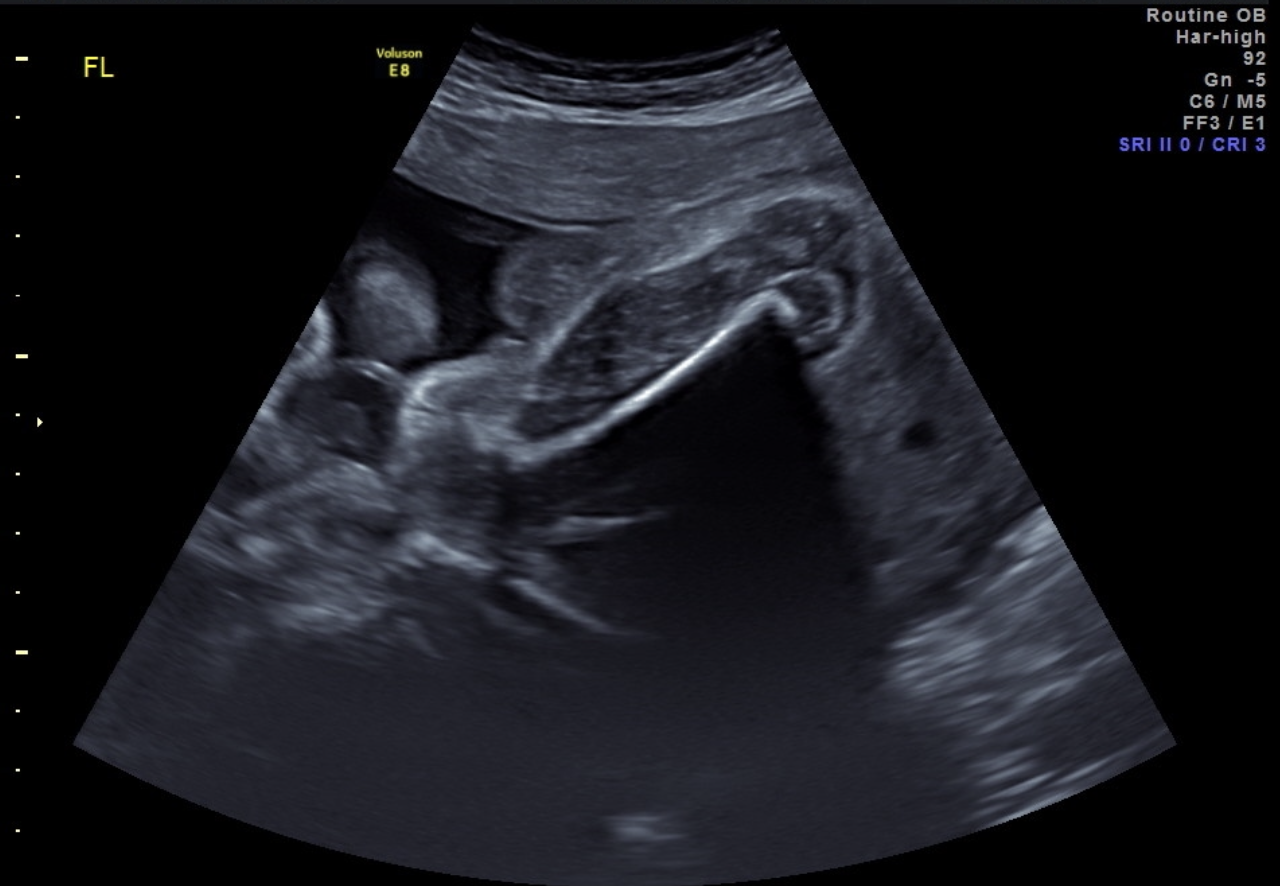

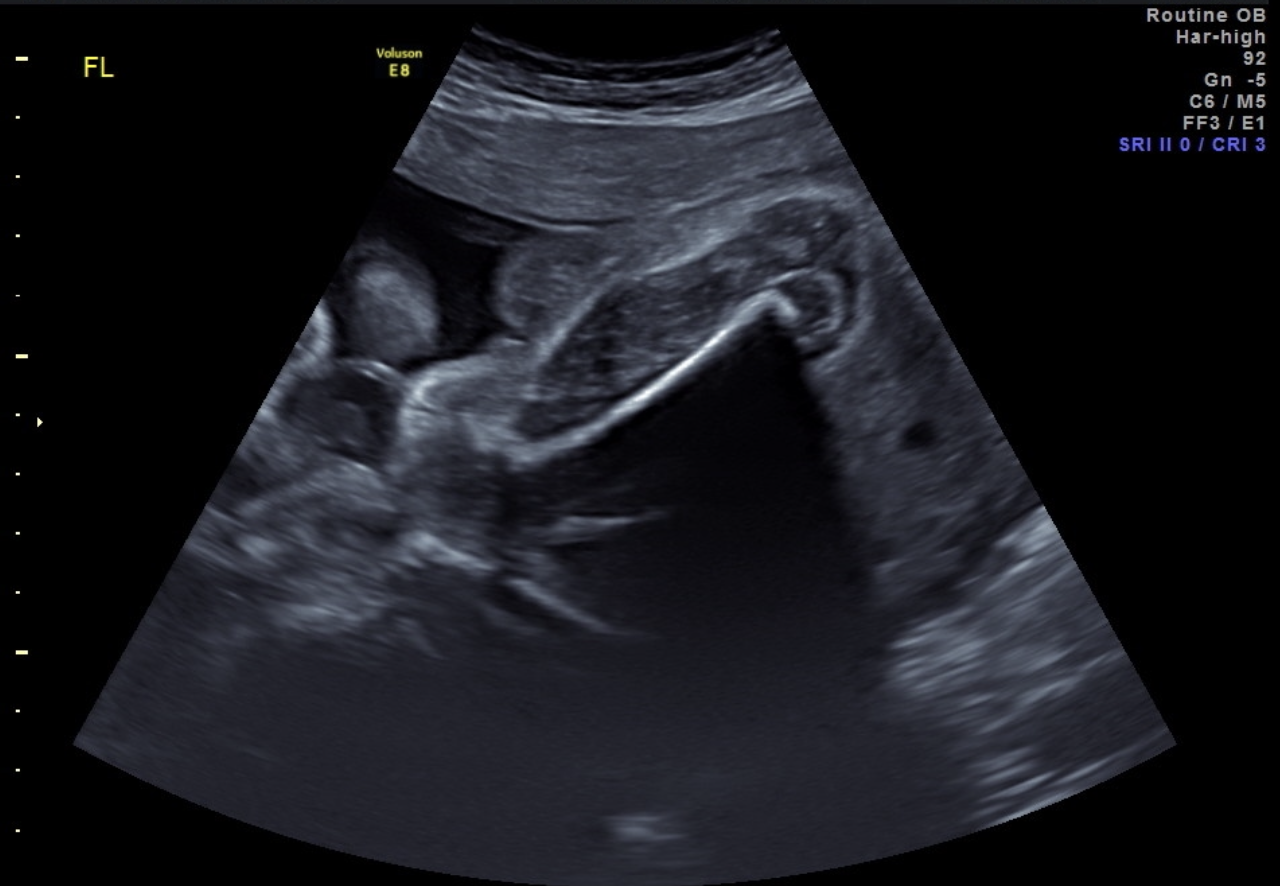

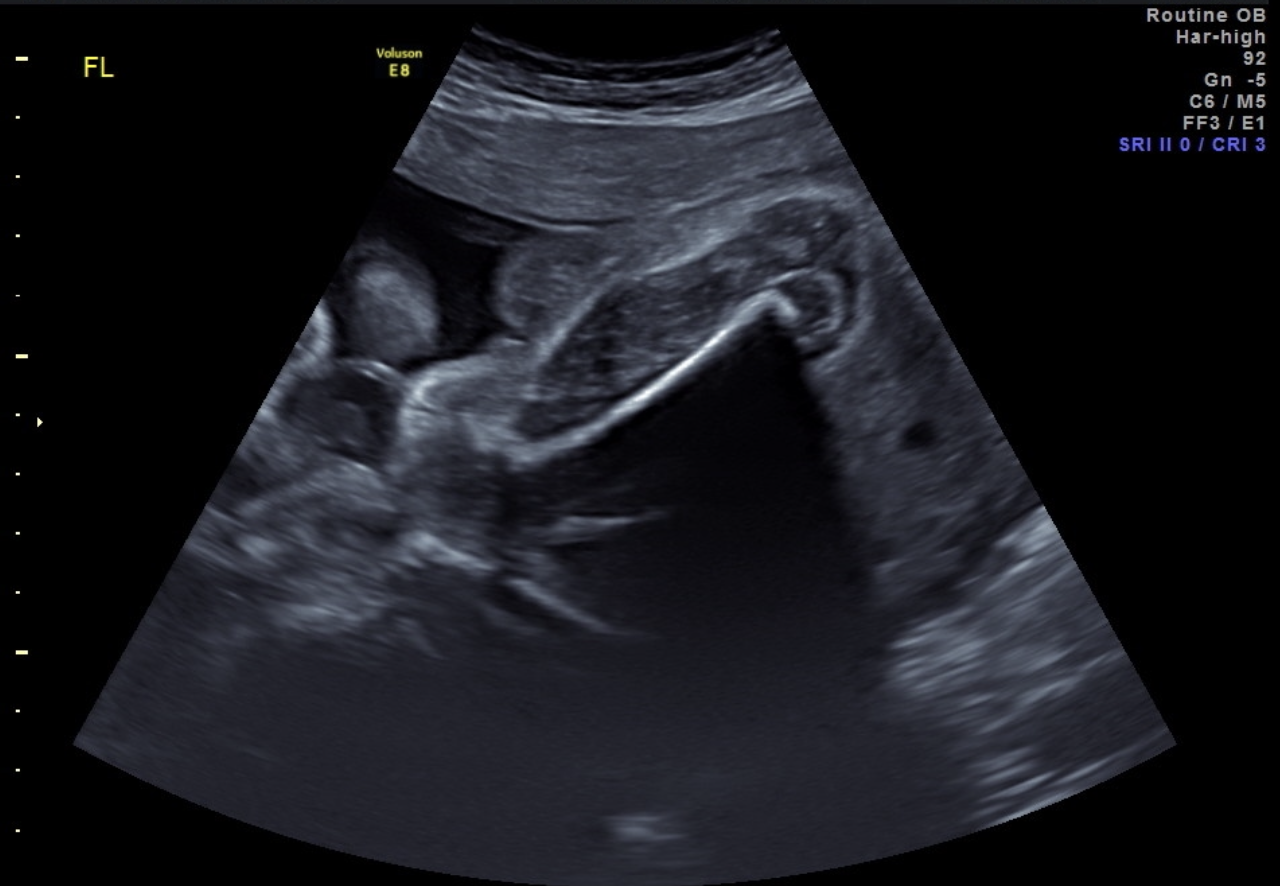

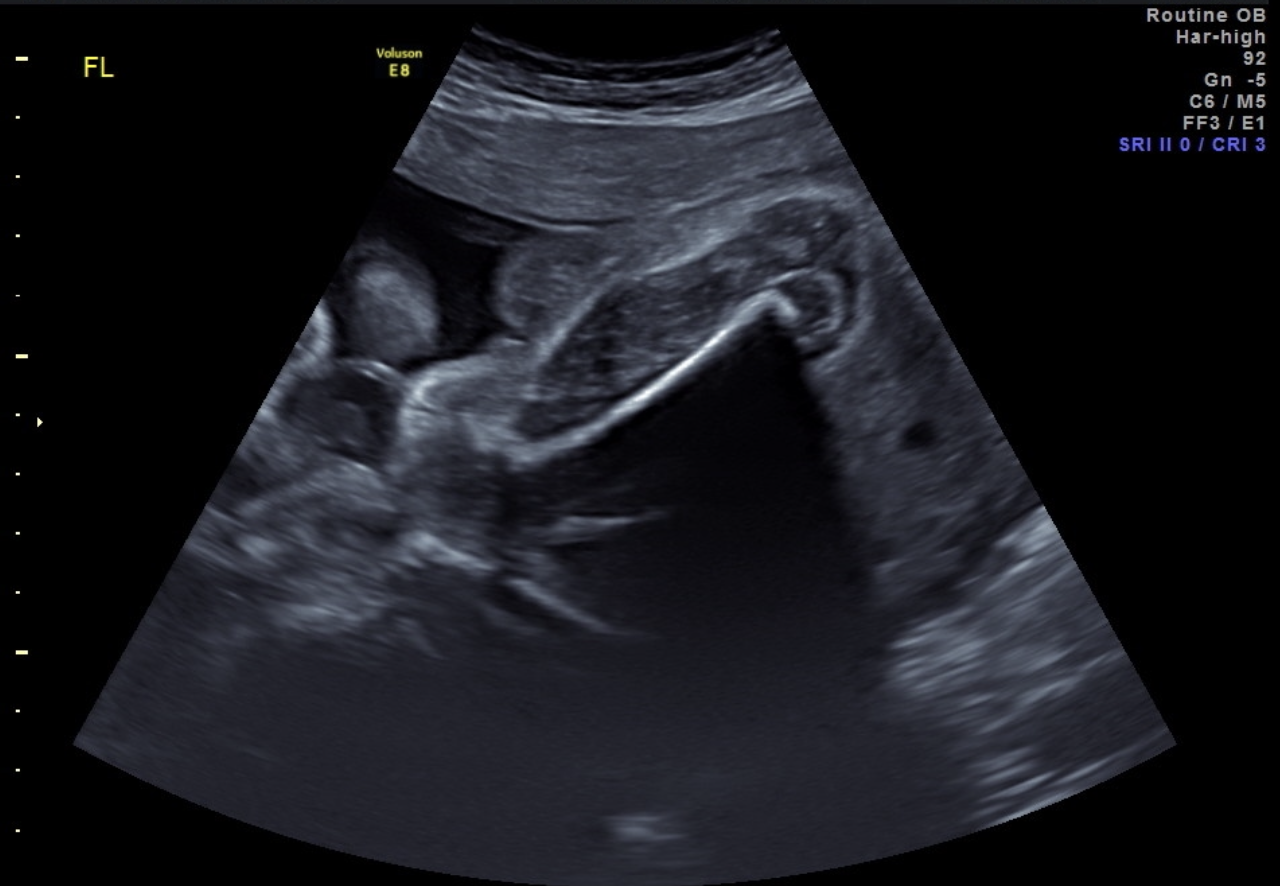

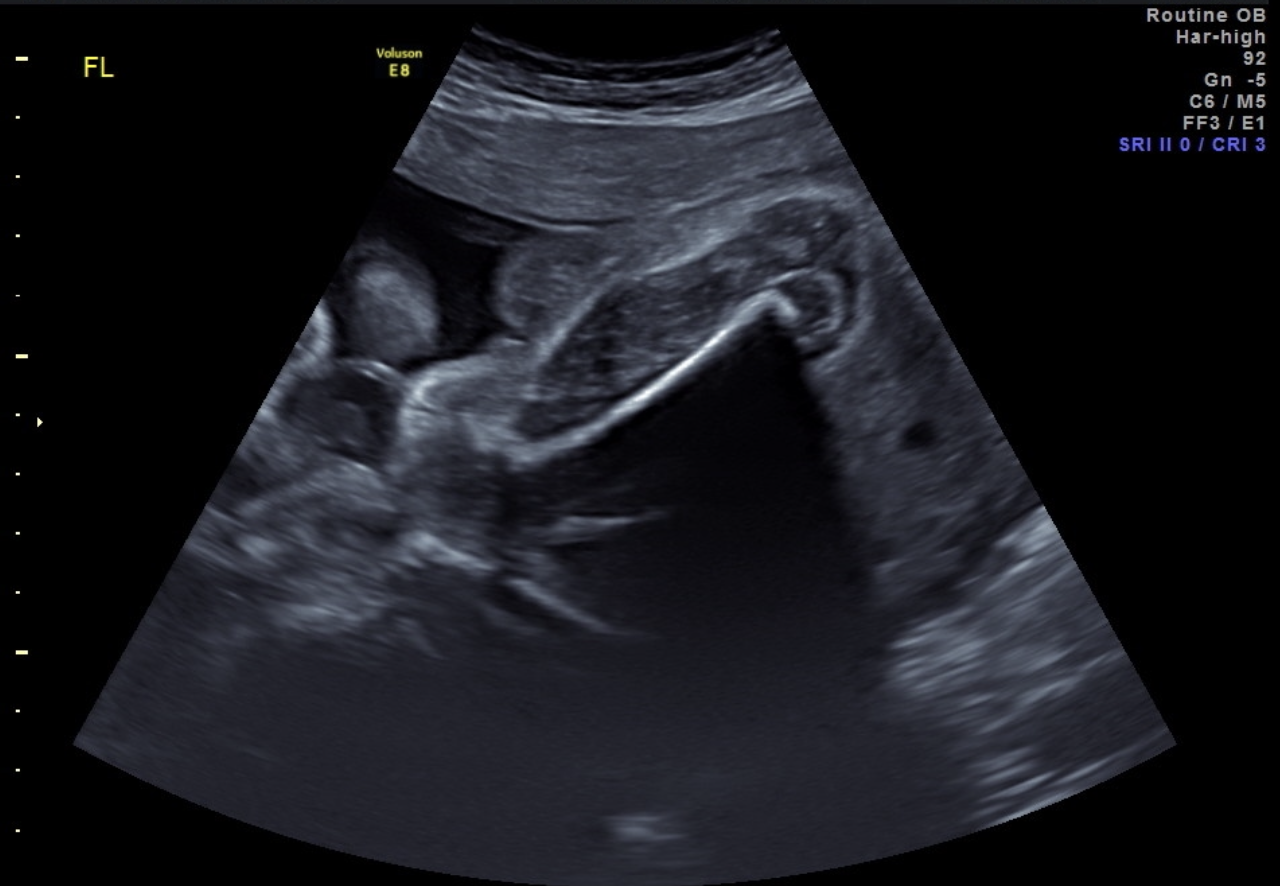

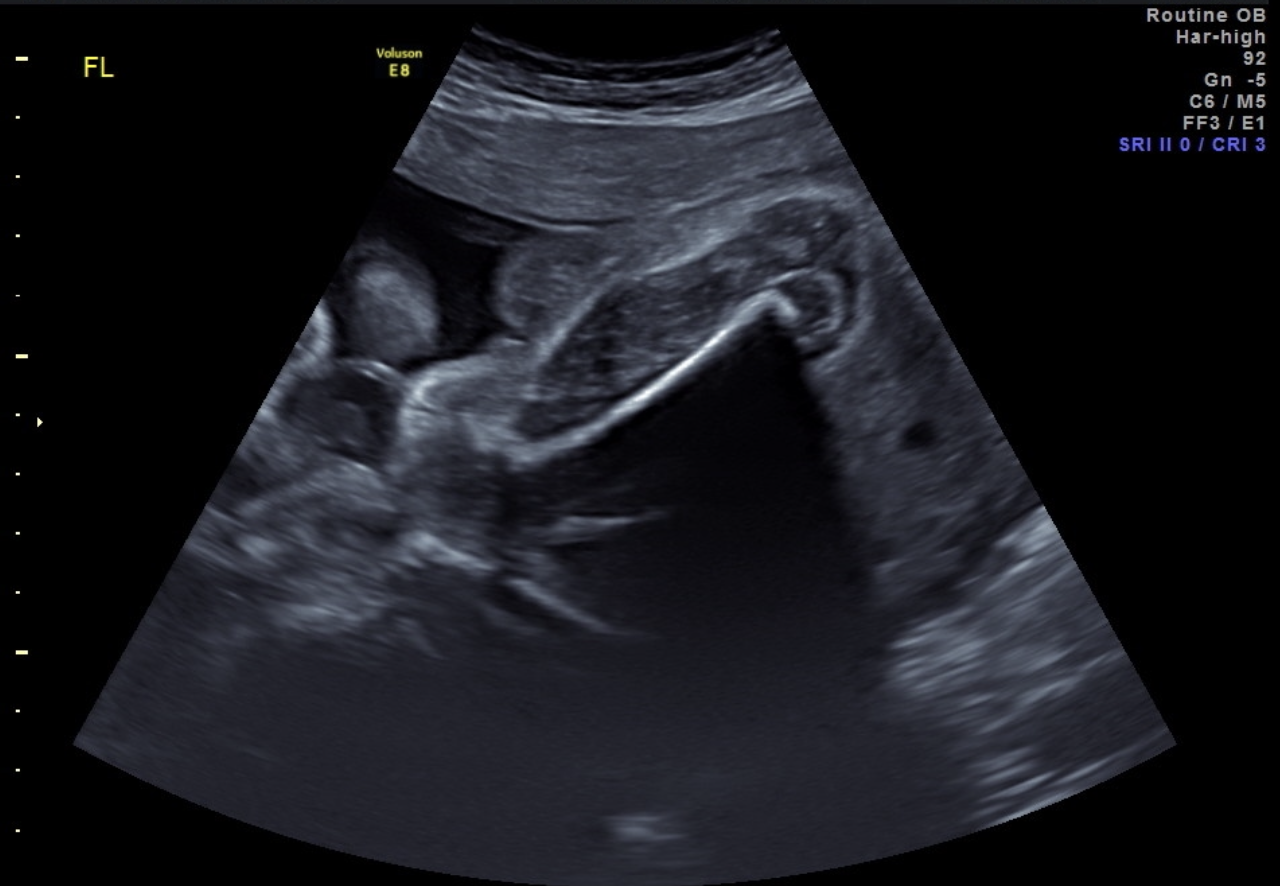

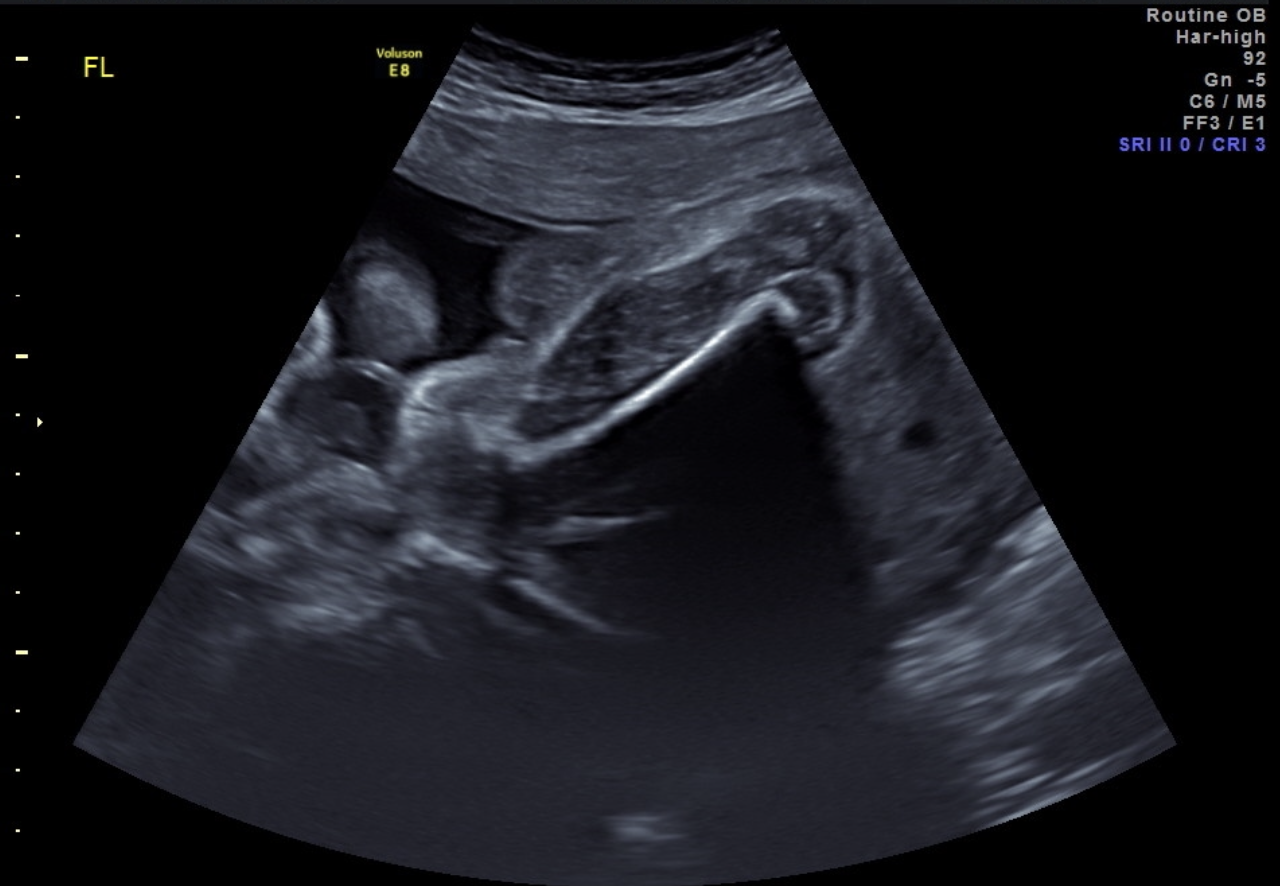

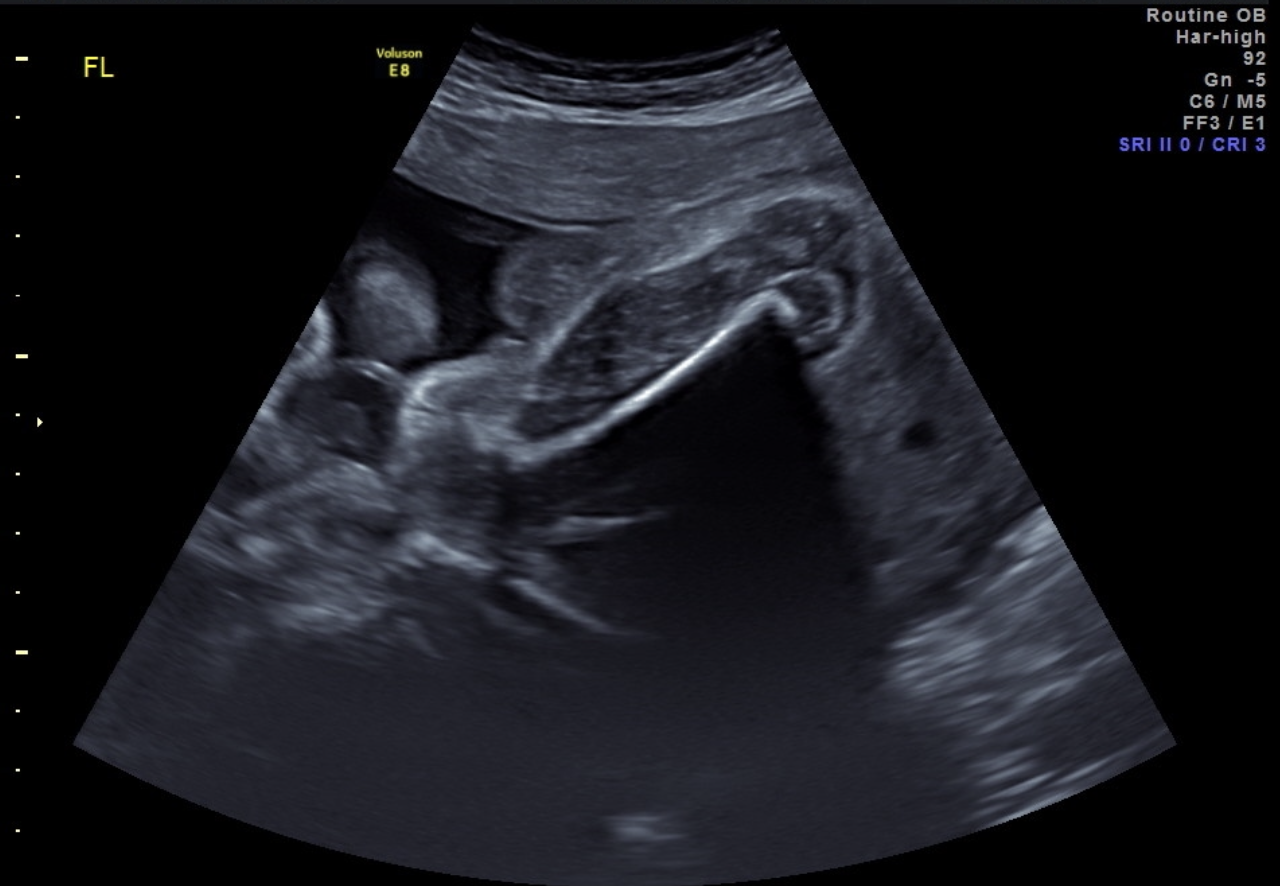

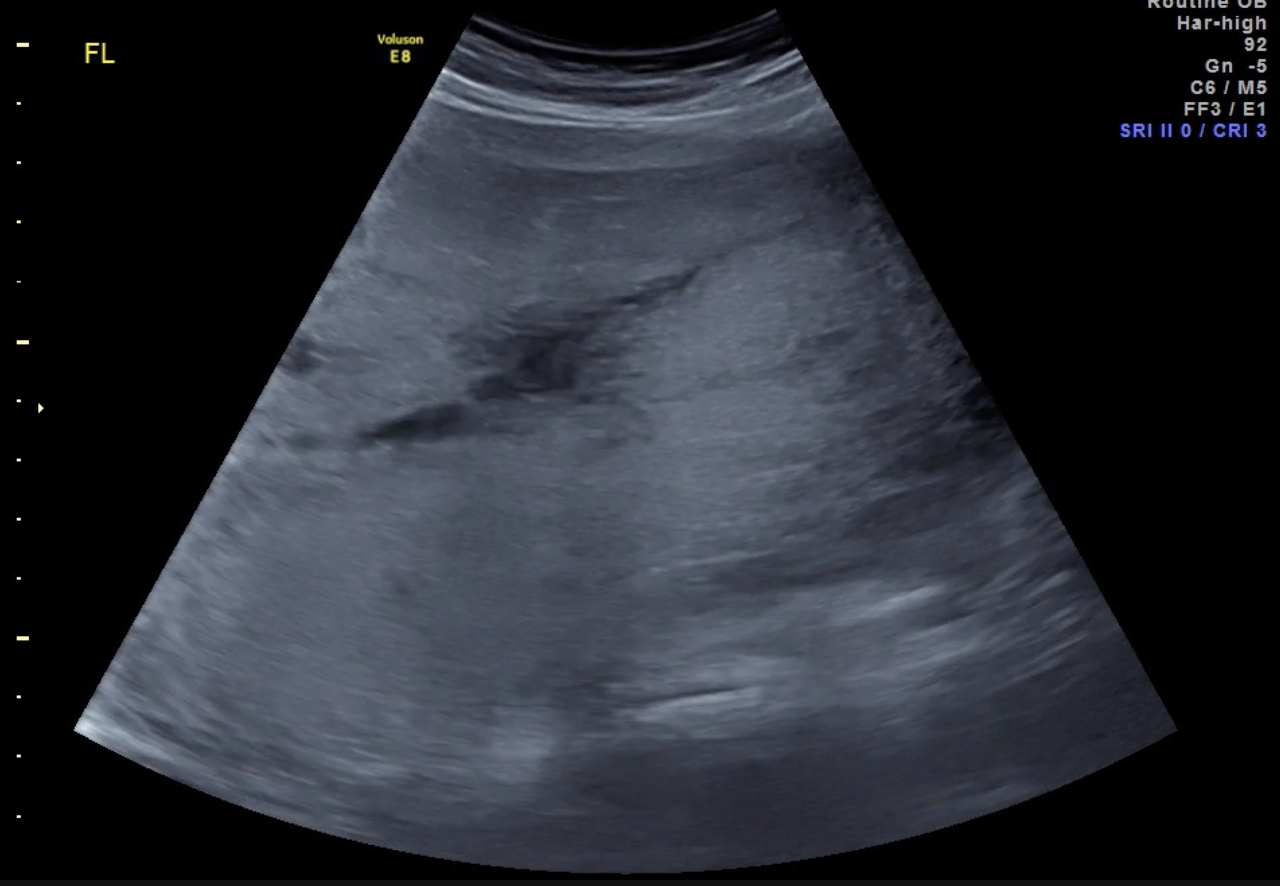

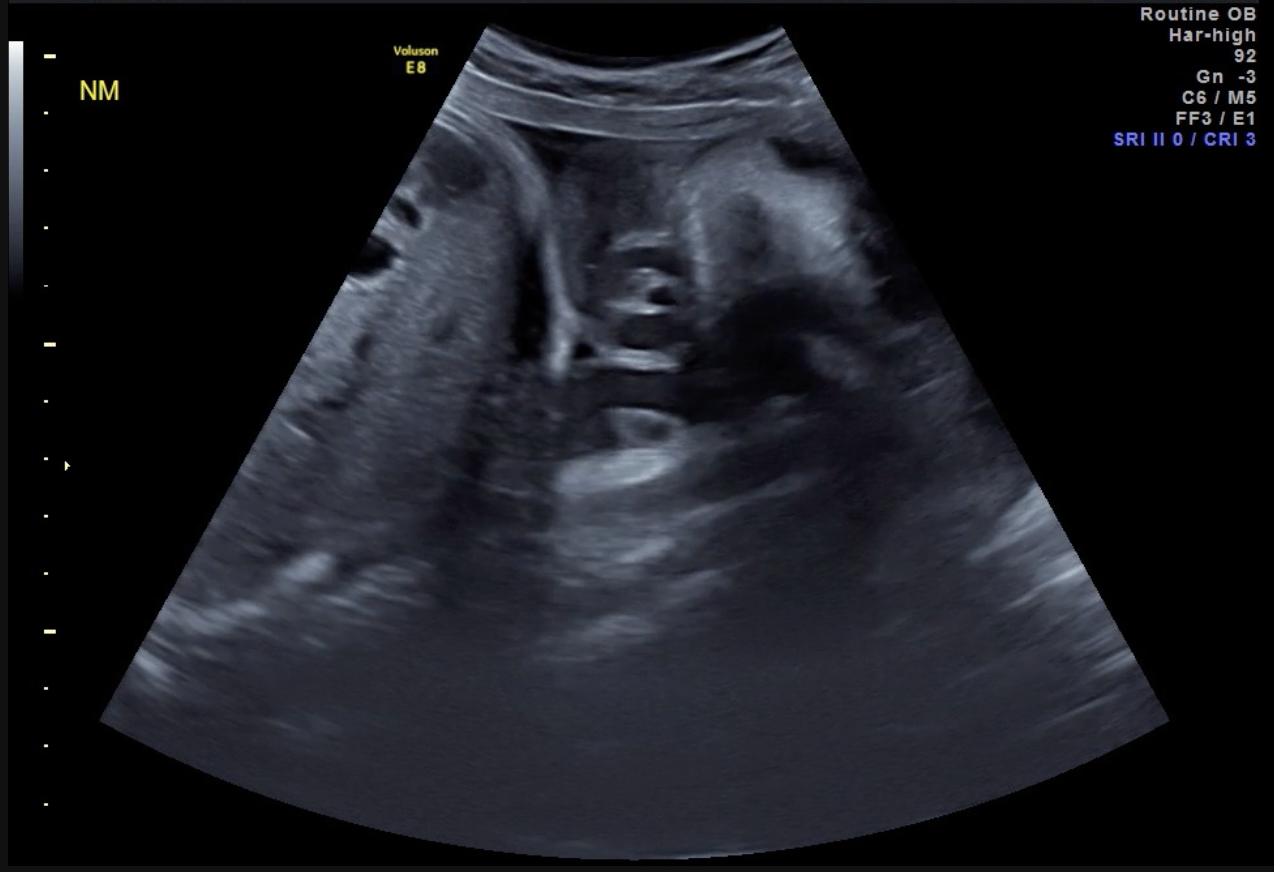


Truncated fly-to

c

Fly-to cineloops are recorded retrospectively, capturing the moments just before the sonographer identifies the ideal image plane. As a result, earlier frames may contain incomplete views of structures or planes unrelated to the target anatomy. It is possible that a novice sonographer may never obtain an ideal image plane and thus the the corresponding fly-to cineloop may contain less usable information that a fly-to cineloop that ends with a standard view. To simulate what might be available from a novice sonographer, we truncated fly-to cineloops obtained by experts by 50 frames and used these suboptimal sequences to to test the model's performance when forced to rely upon inputs similar to those a novice might capture.


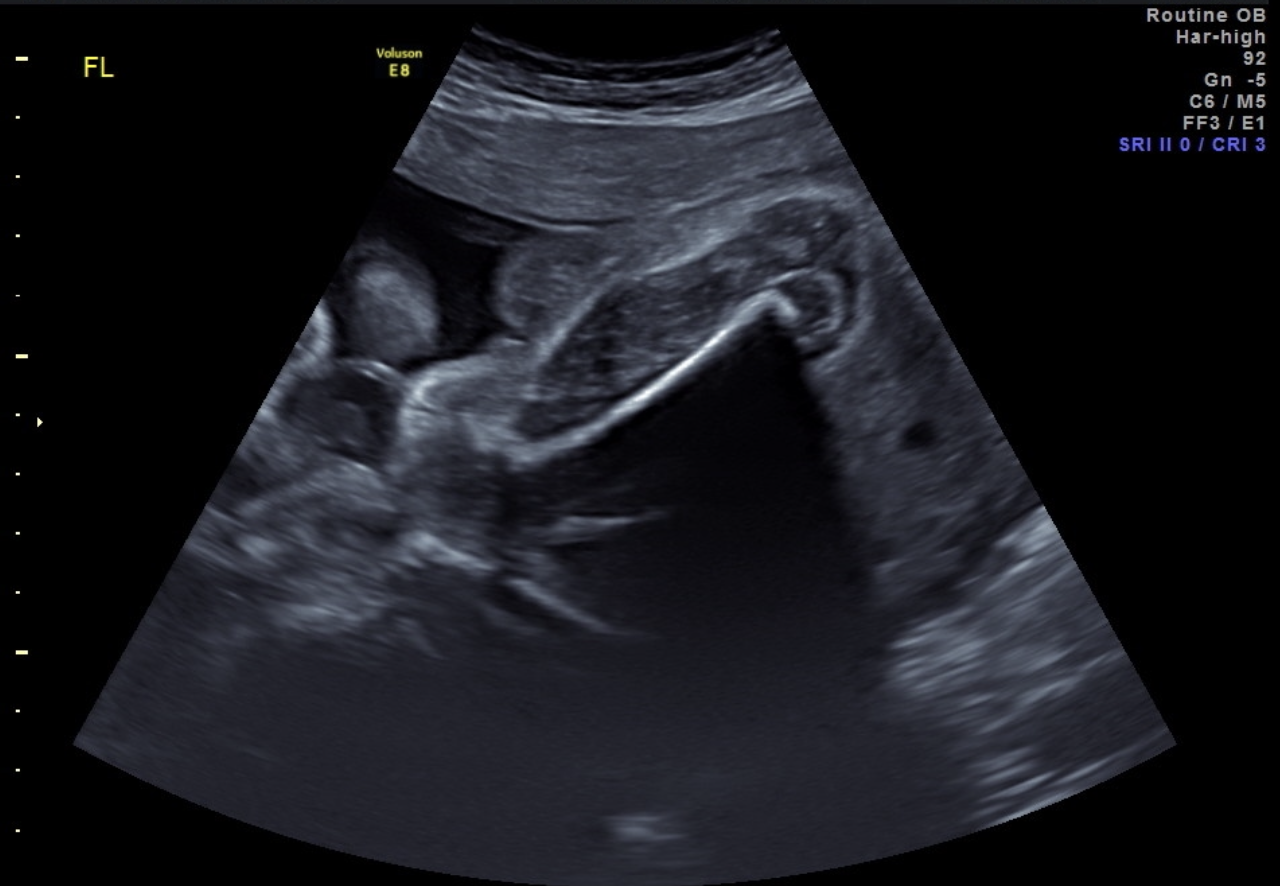

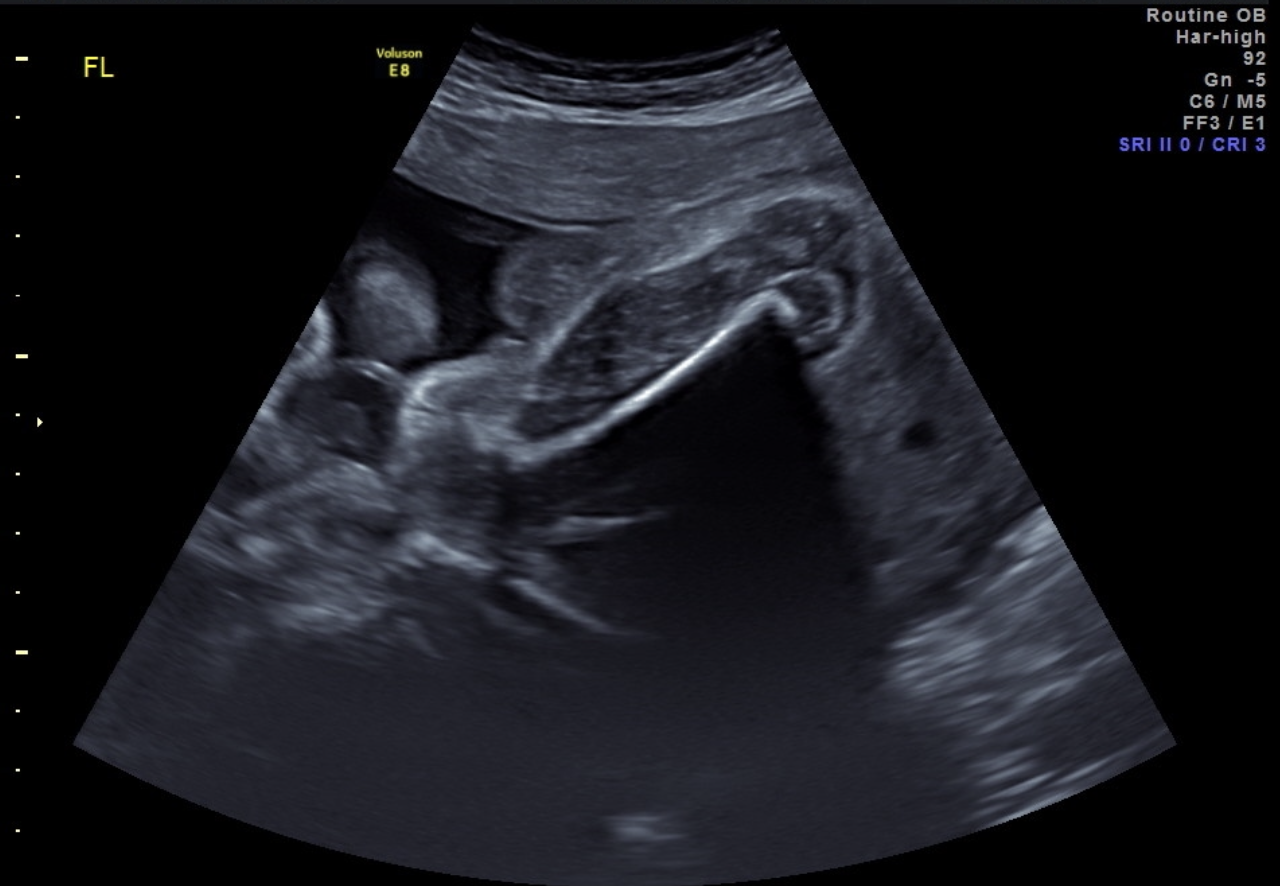

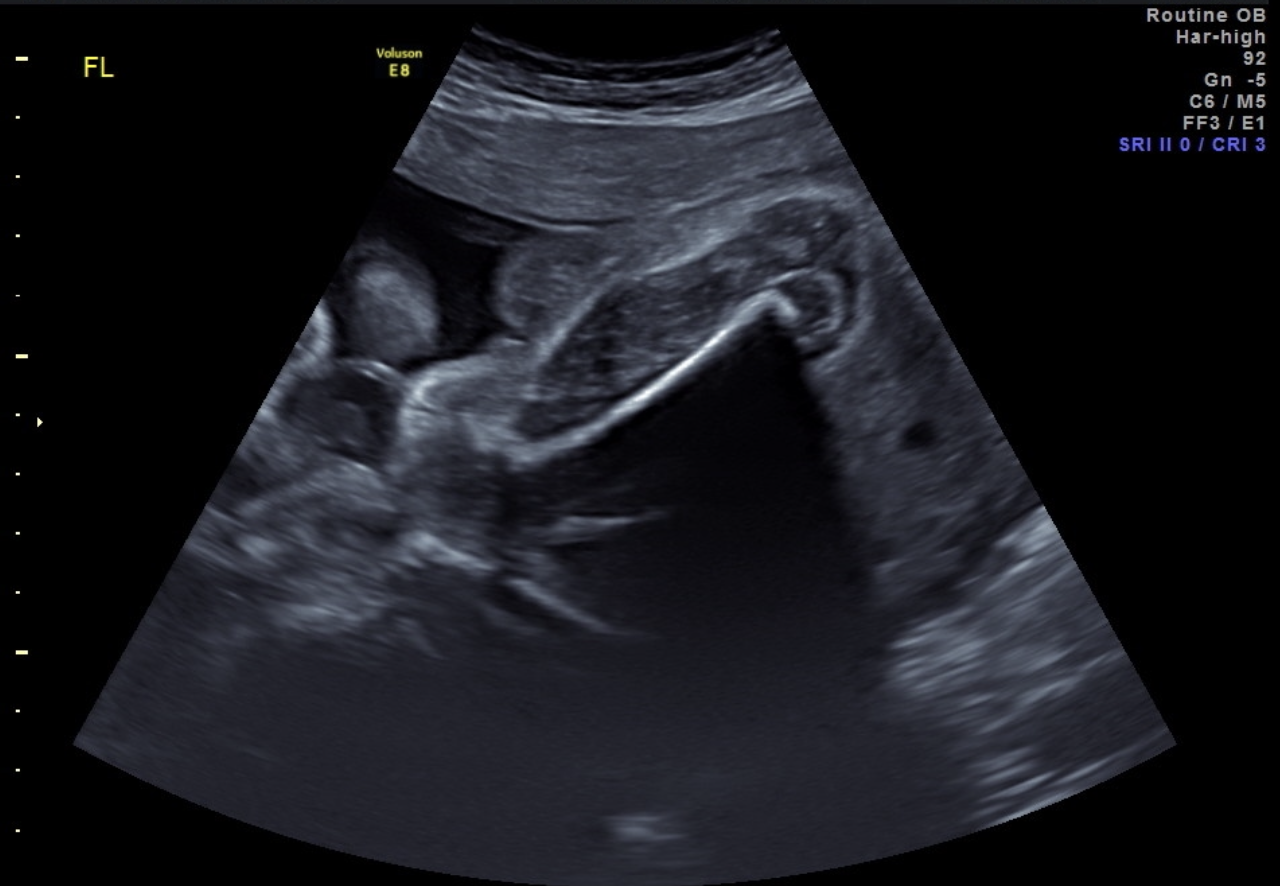

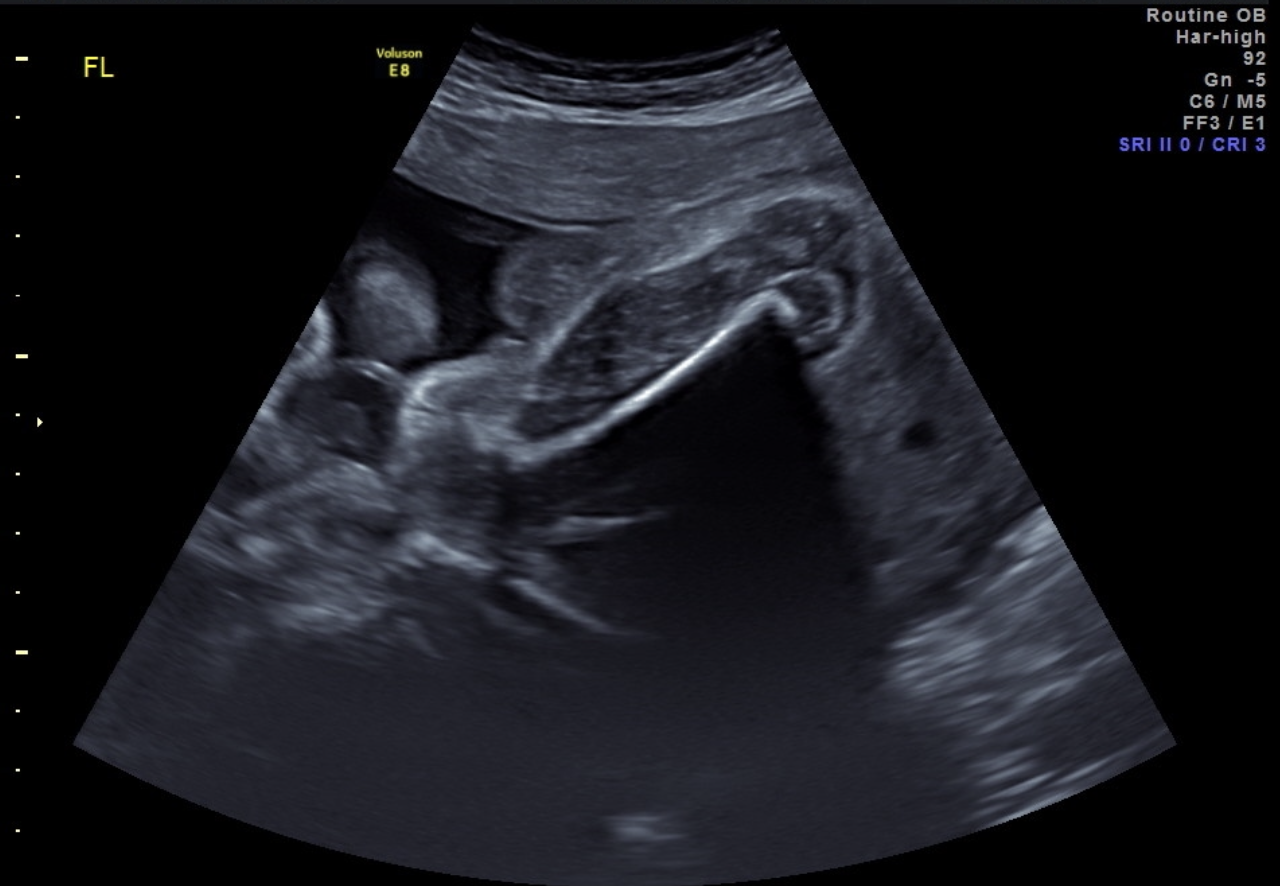

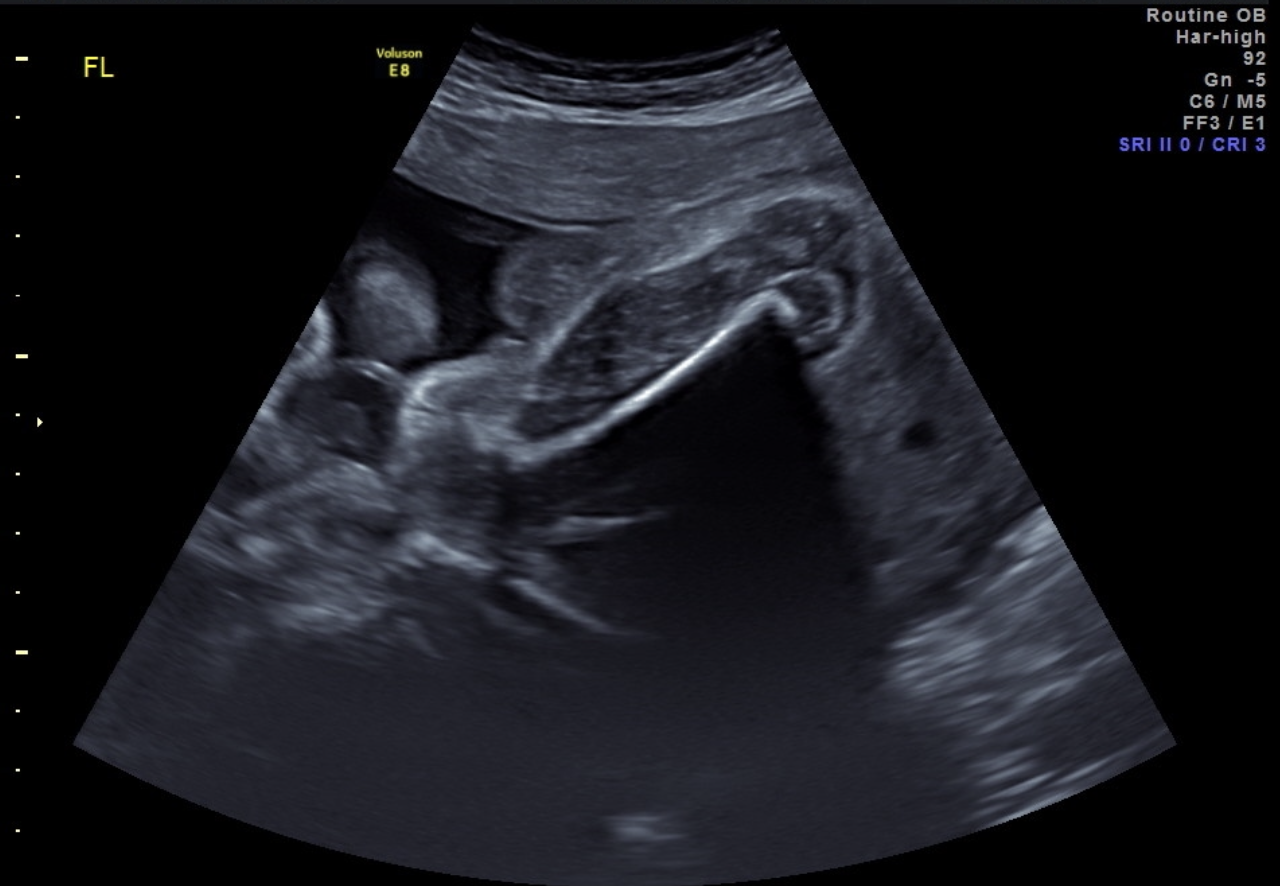

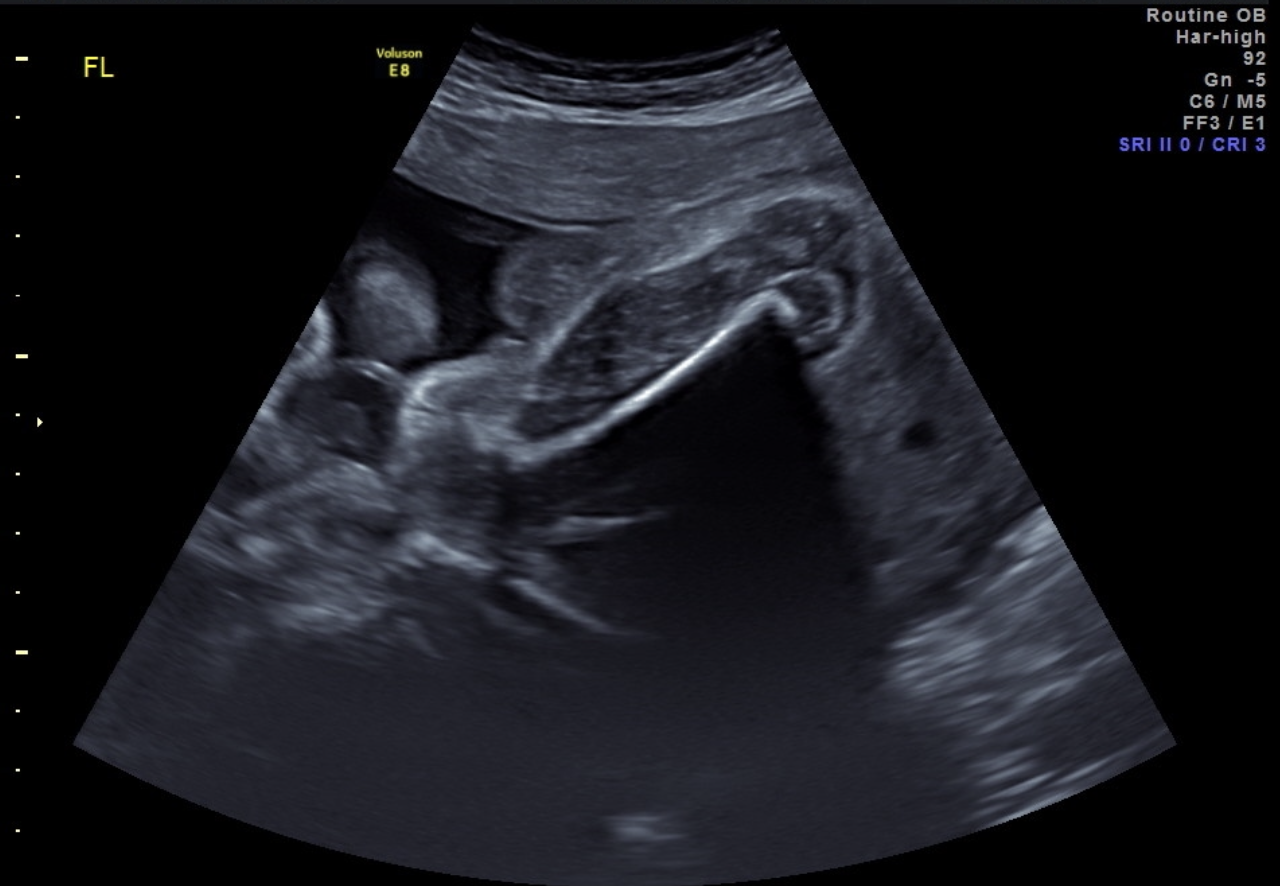

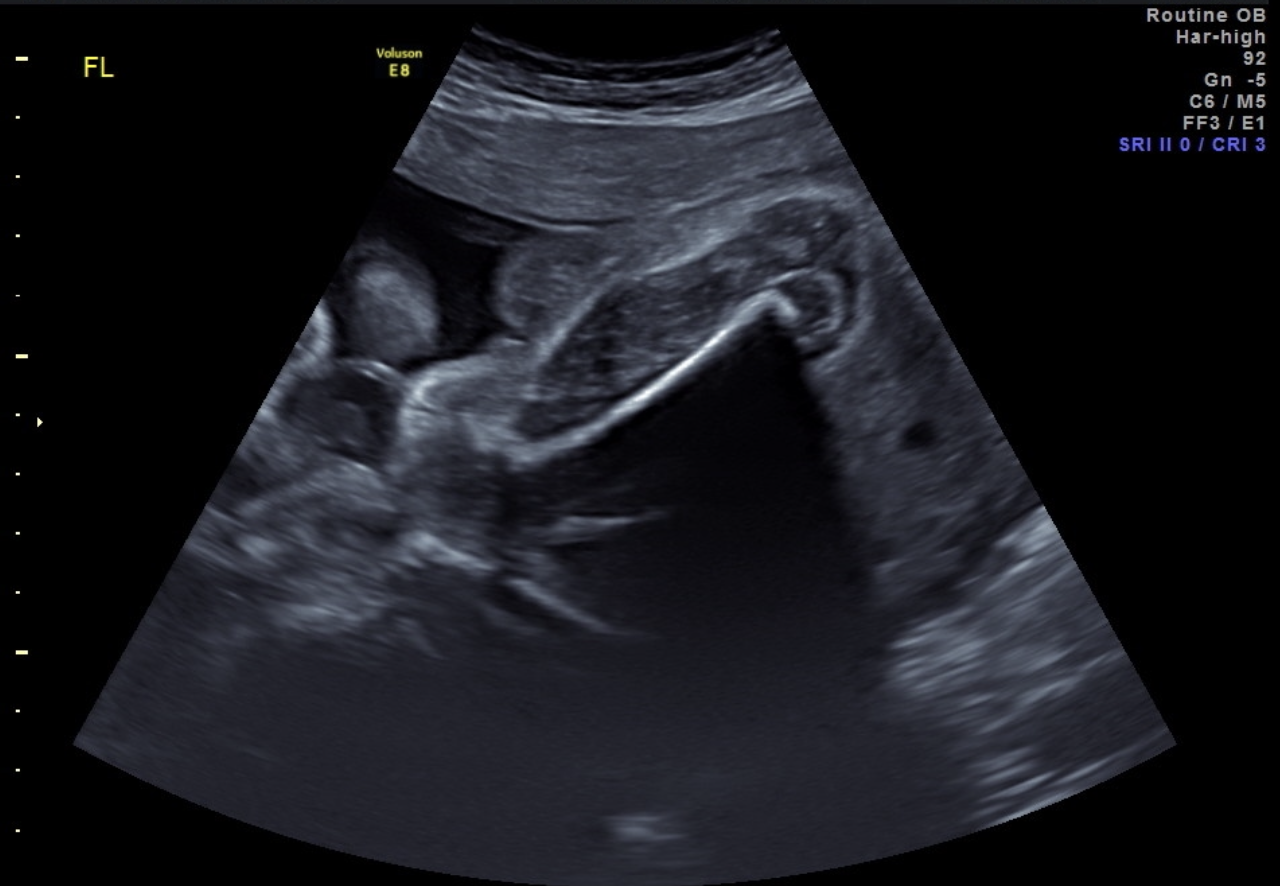

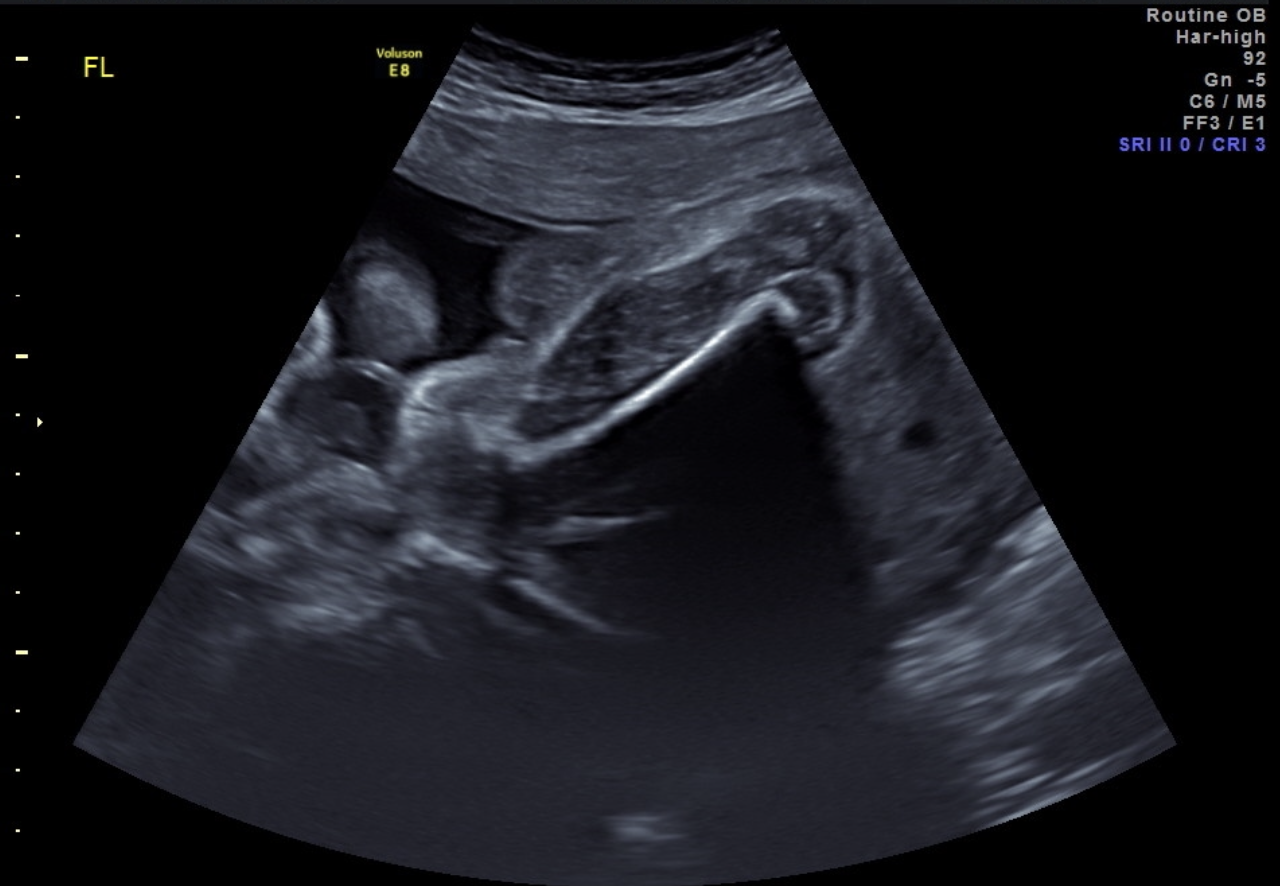

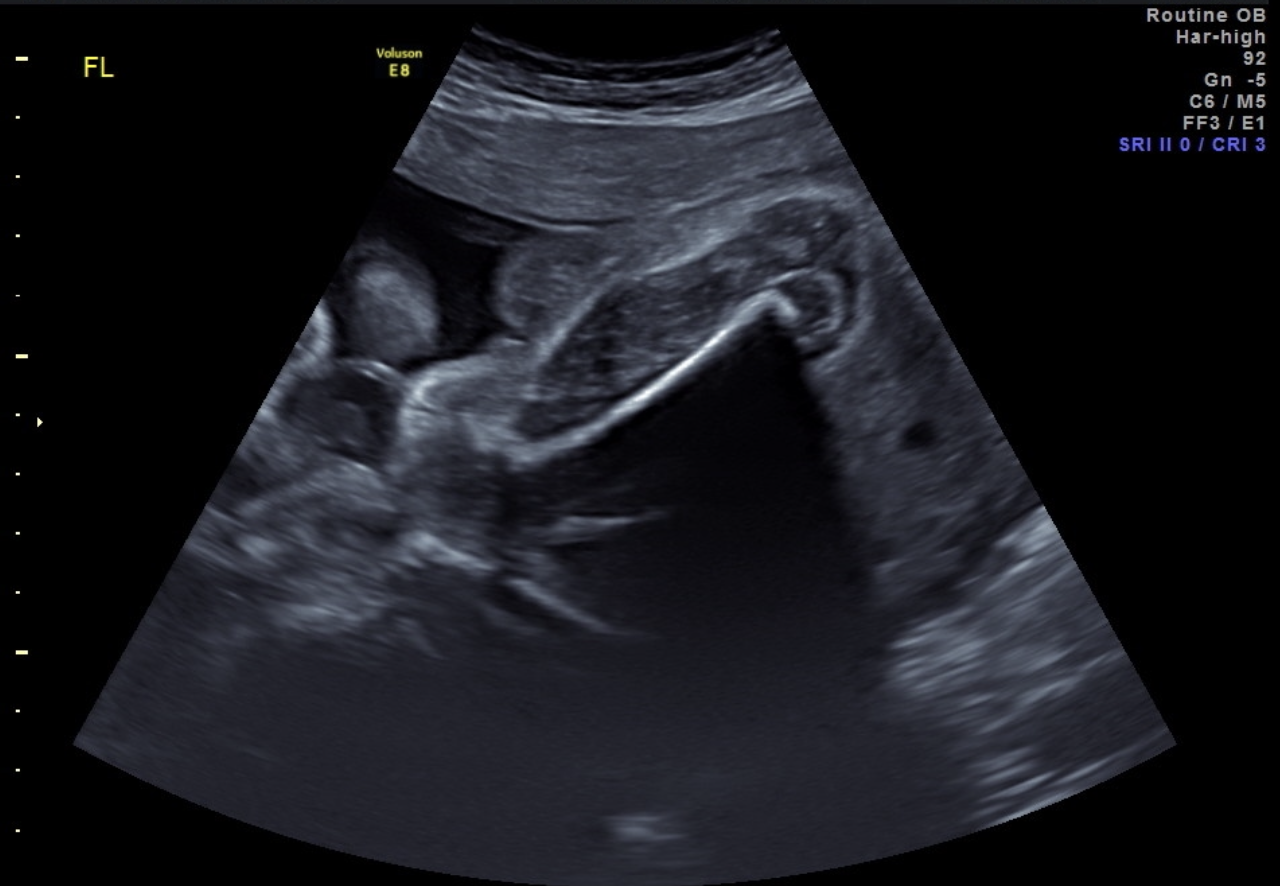

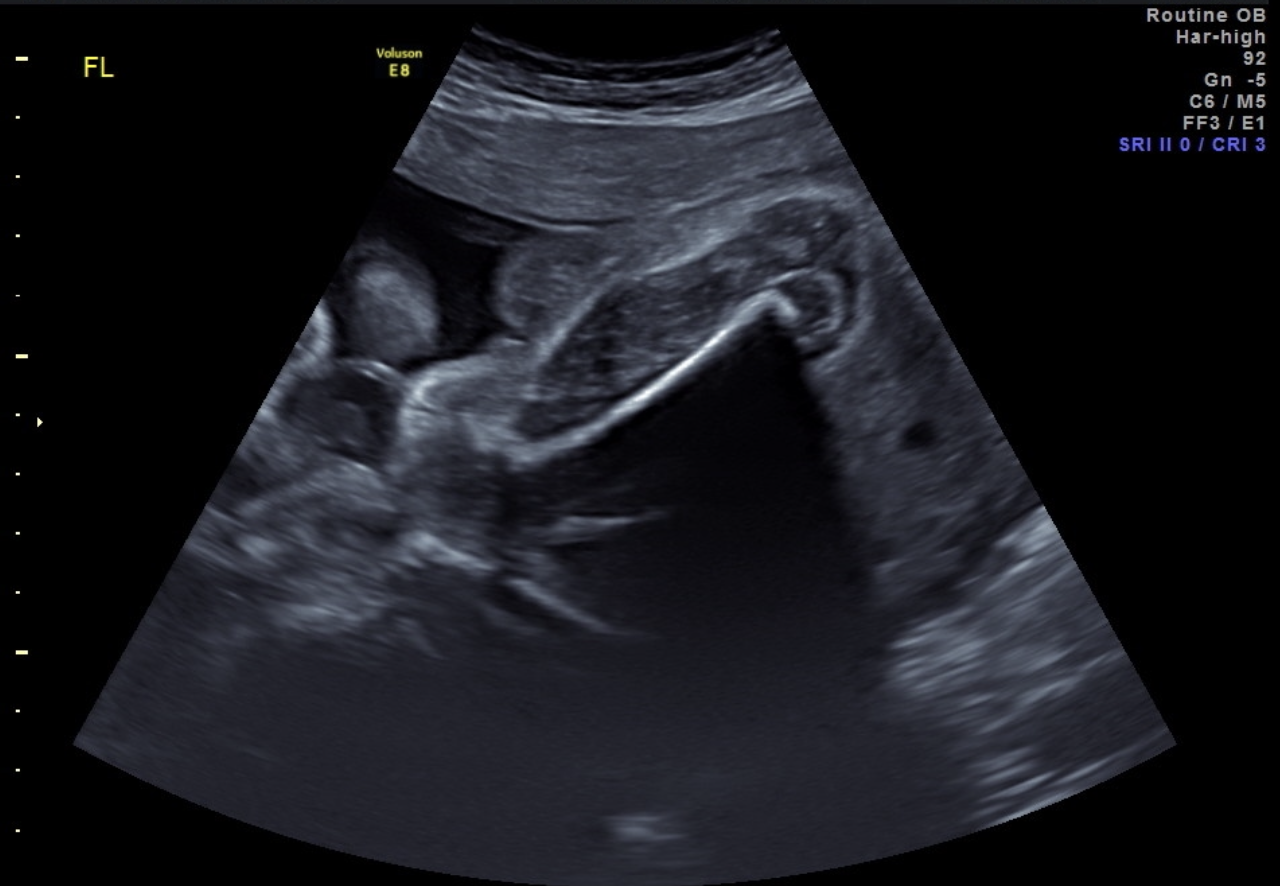

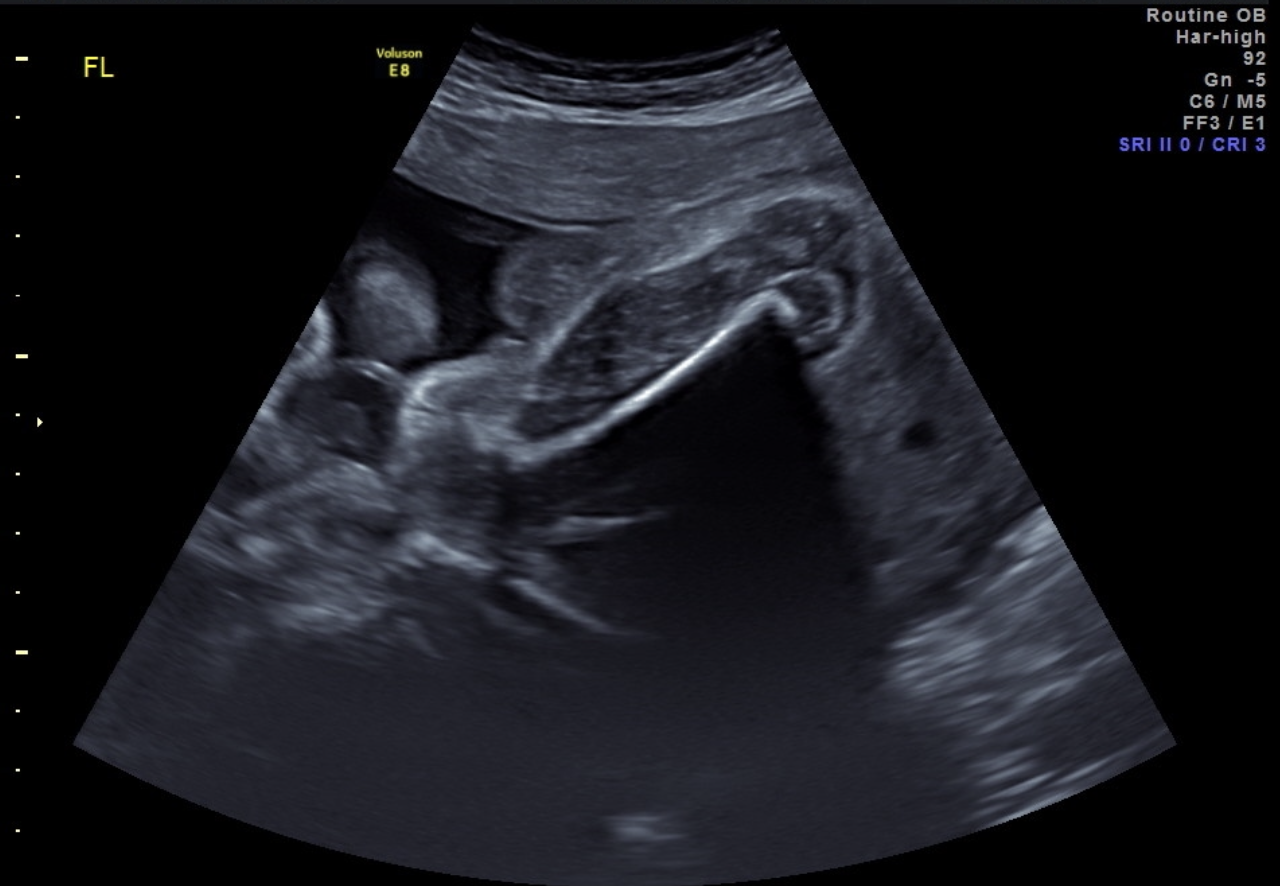

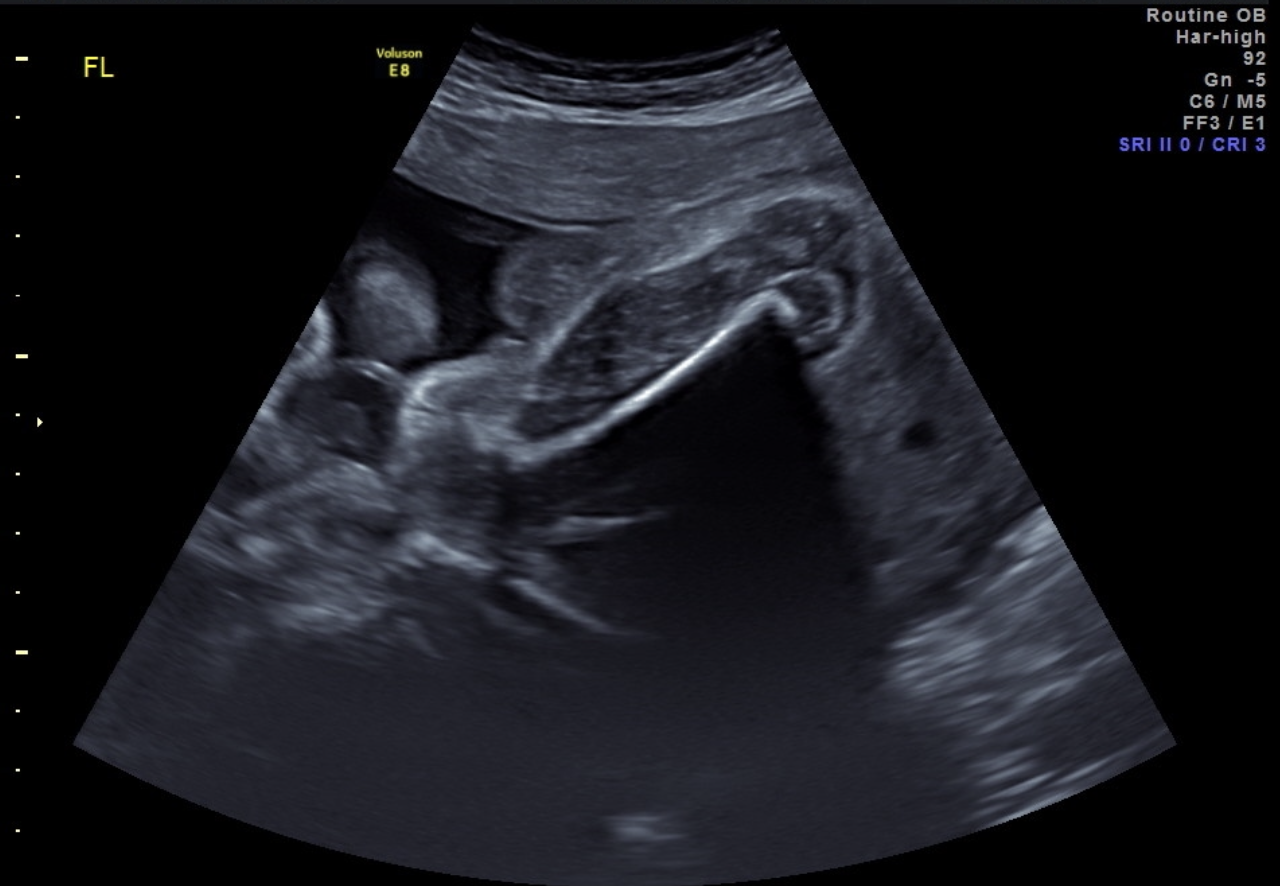

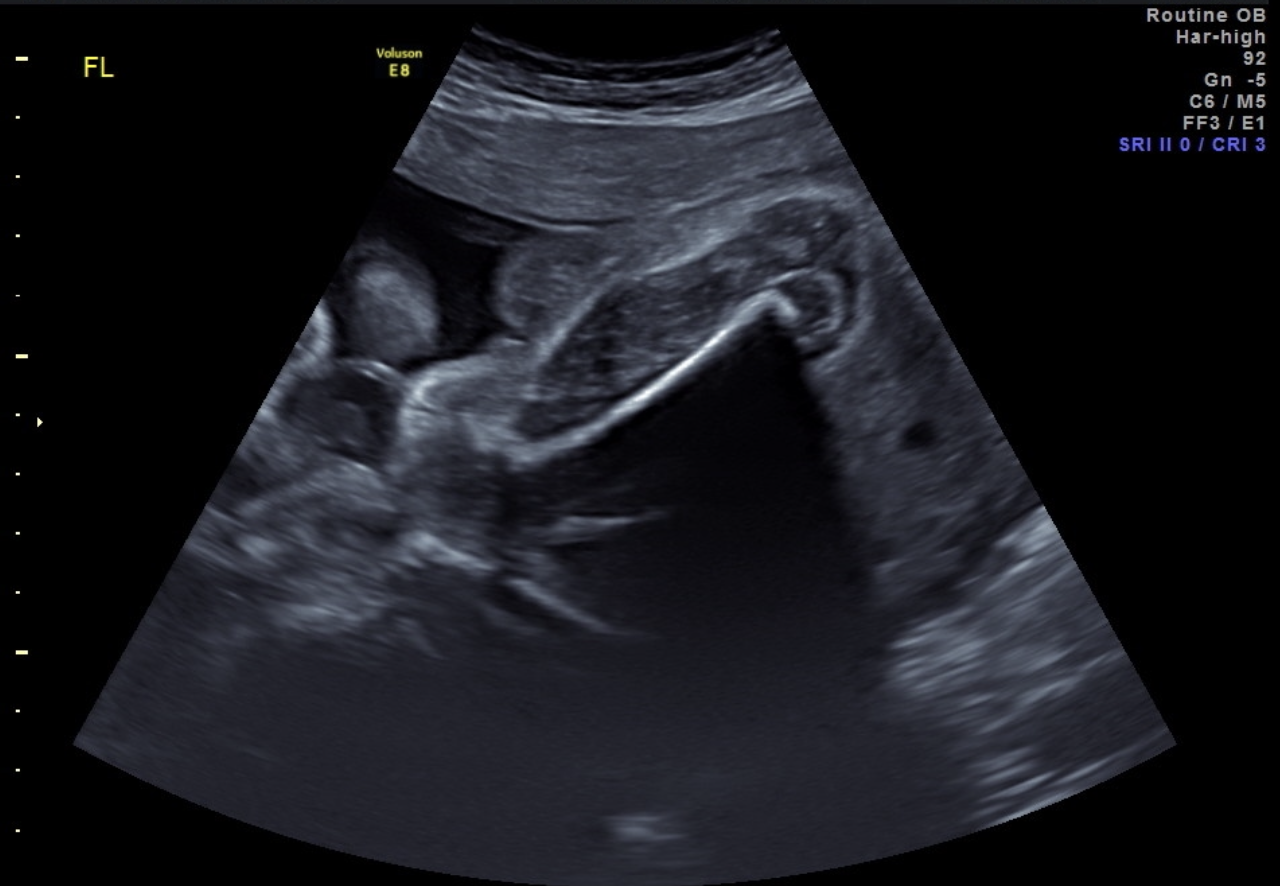

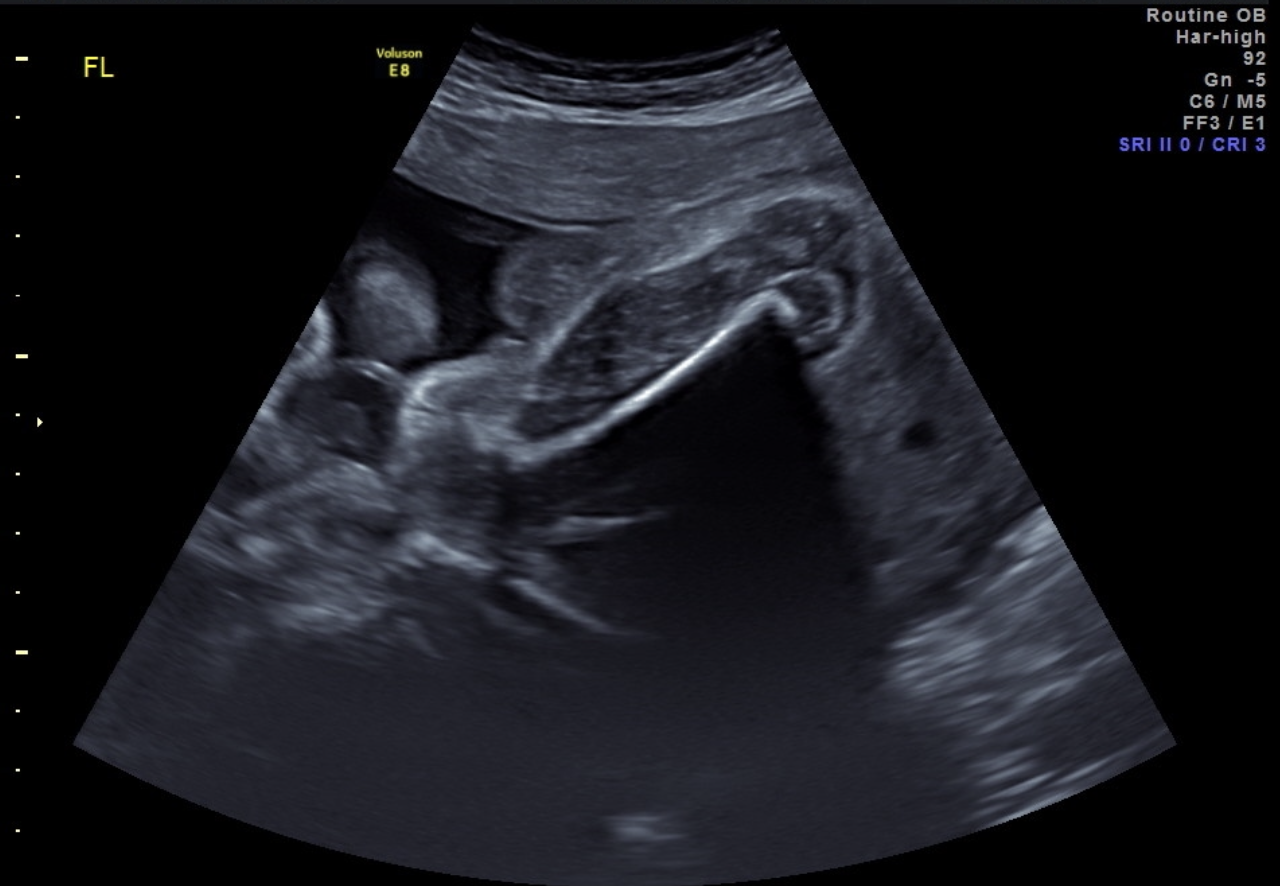

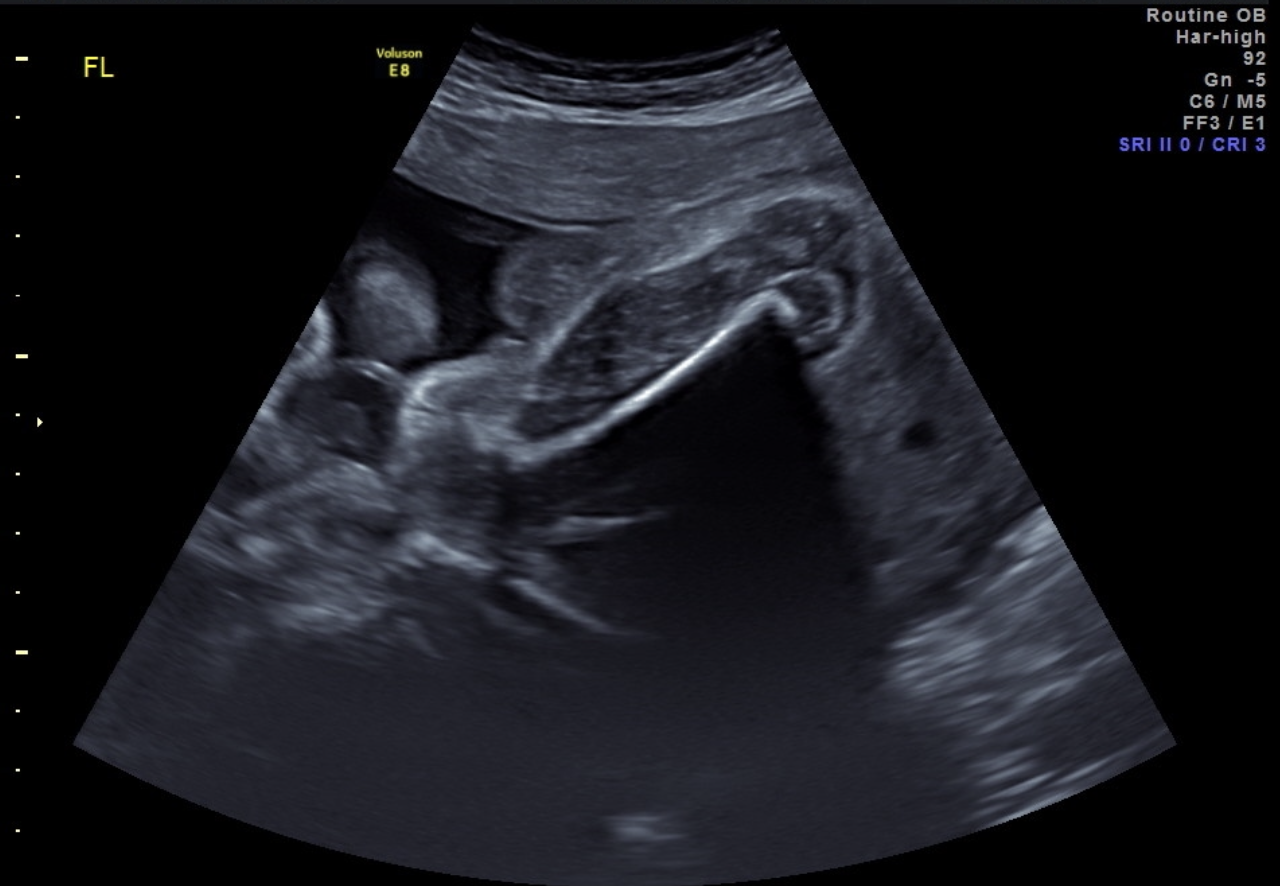

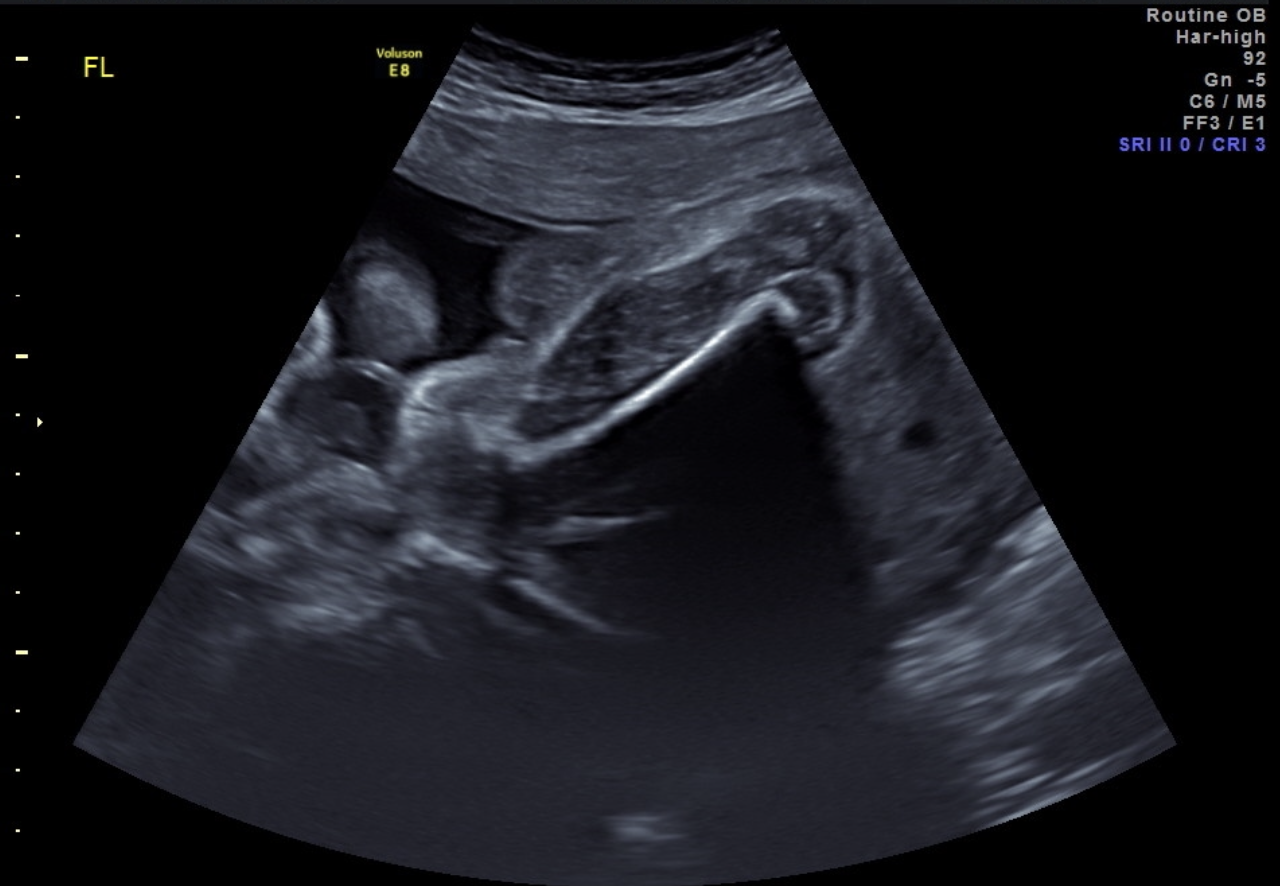

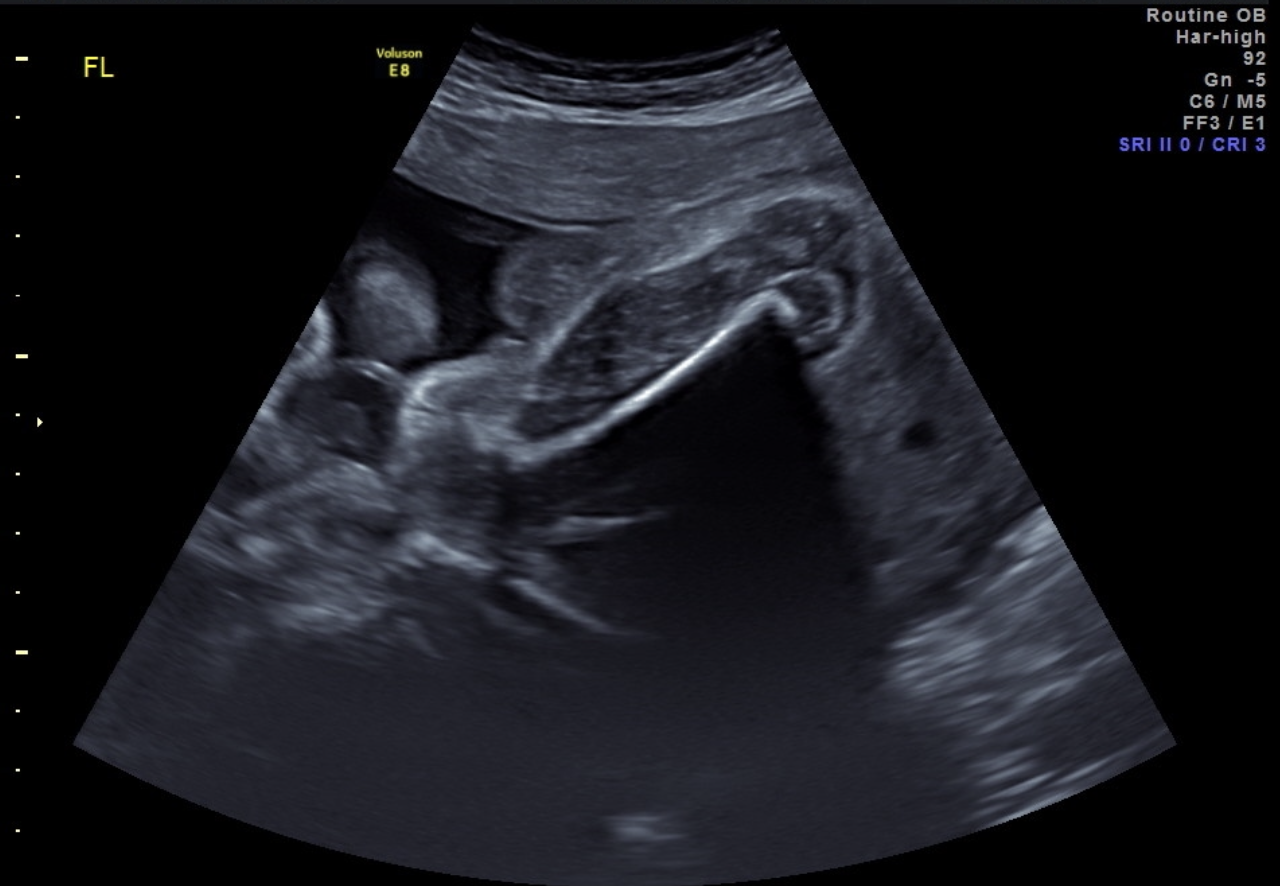

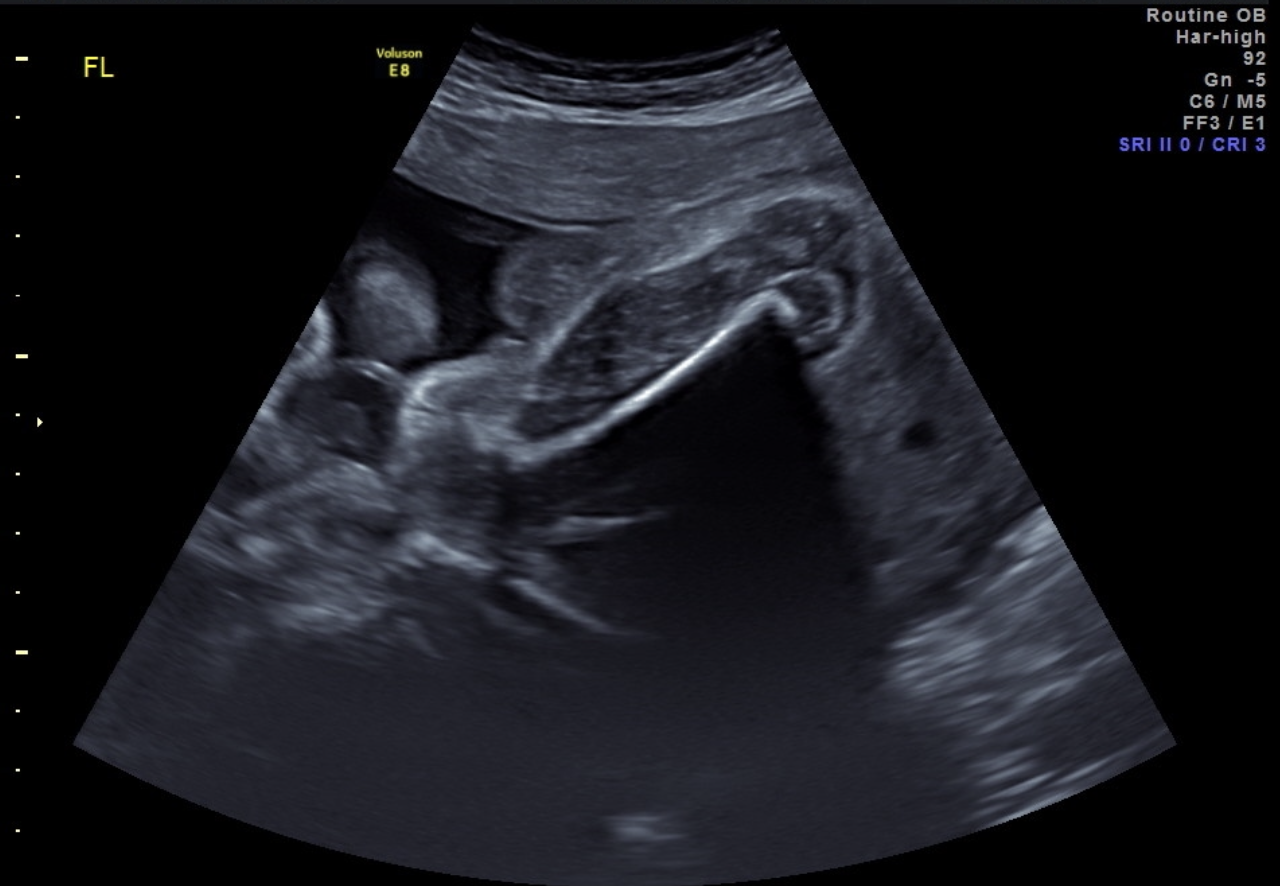

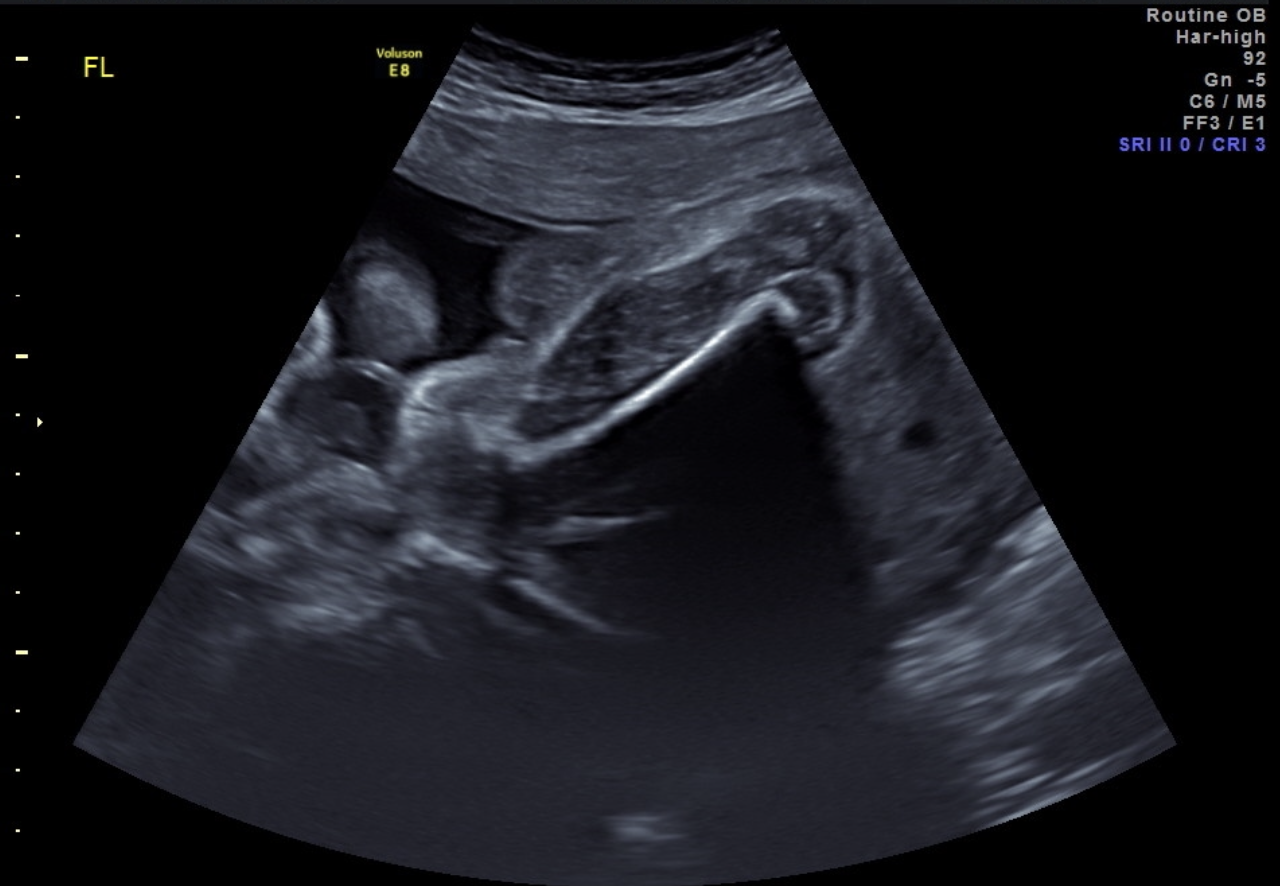

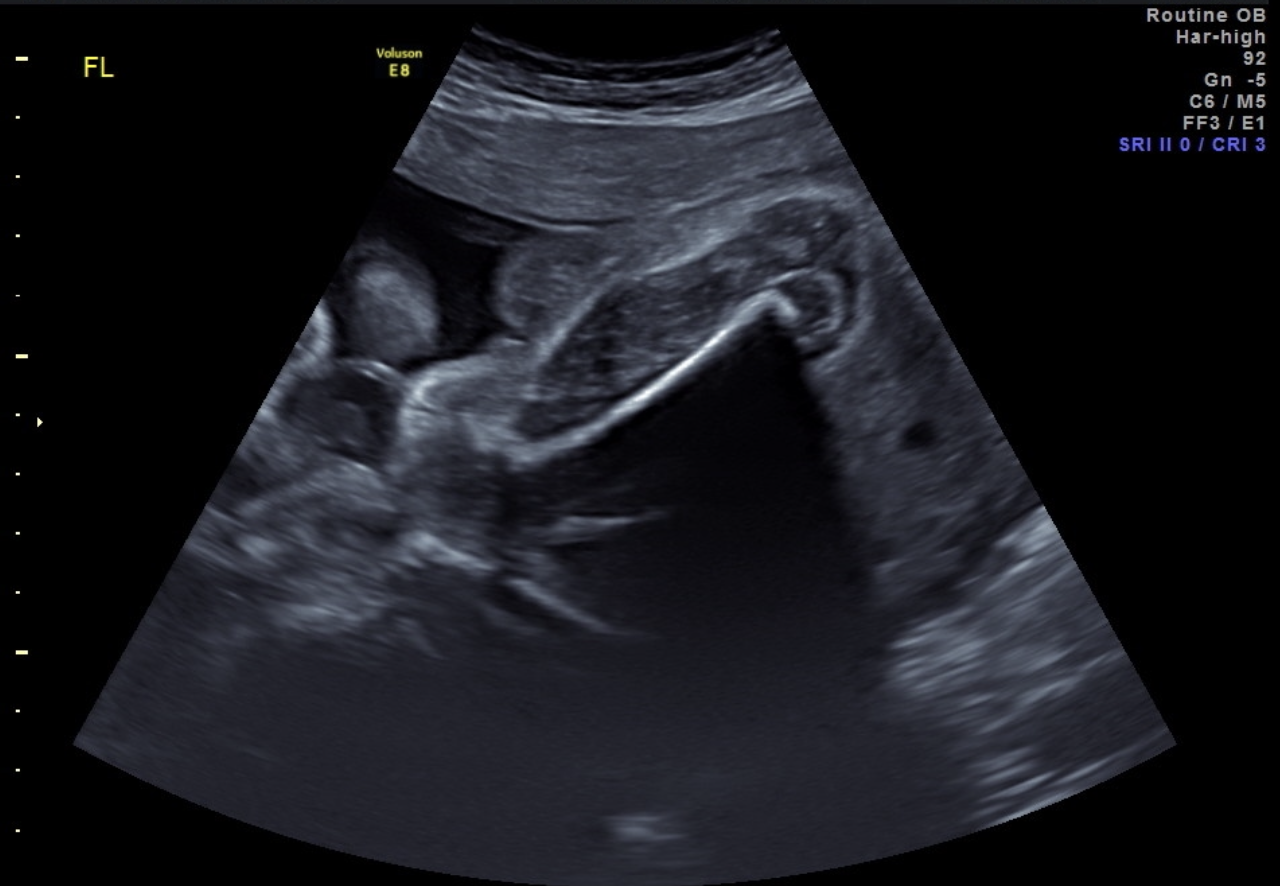

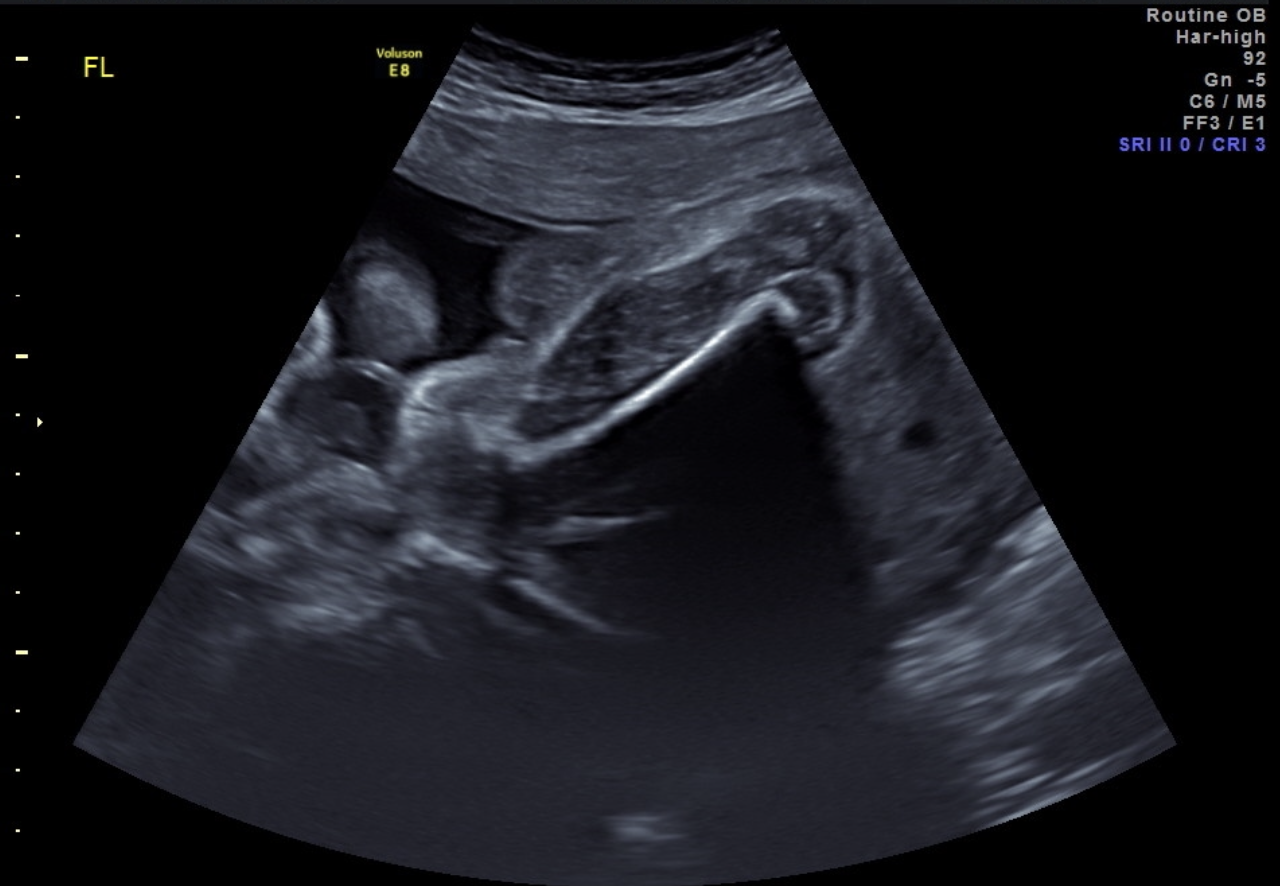

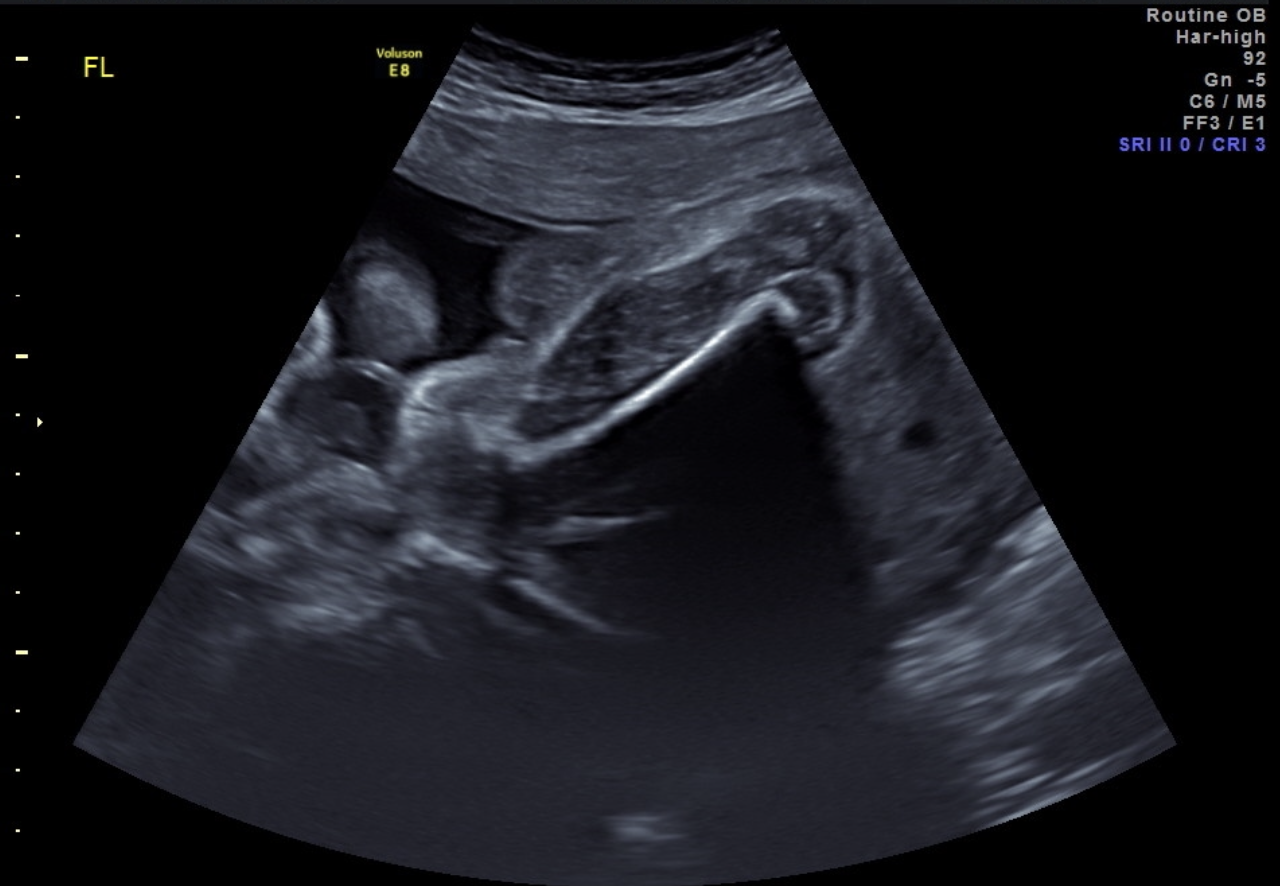

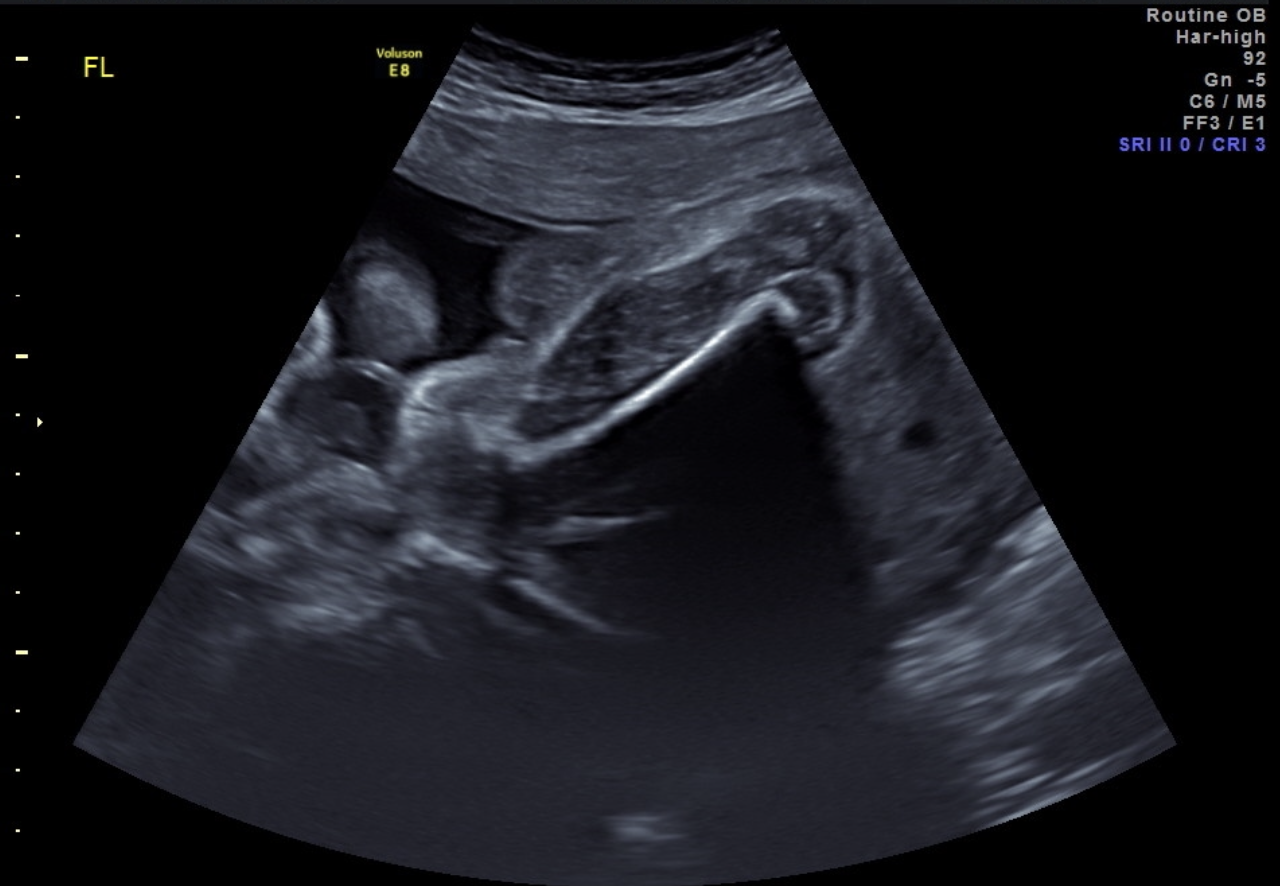

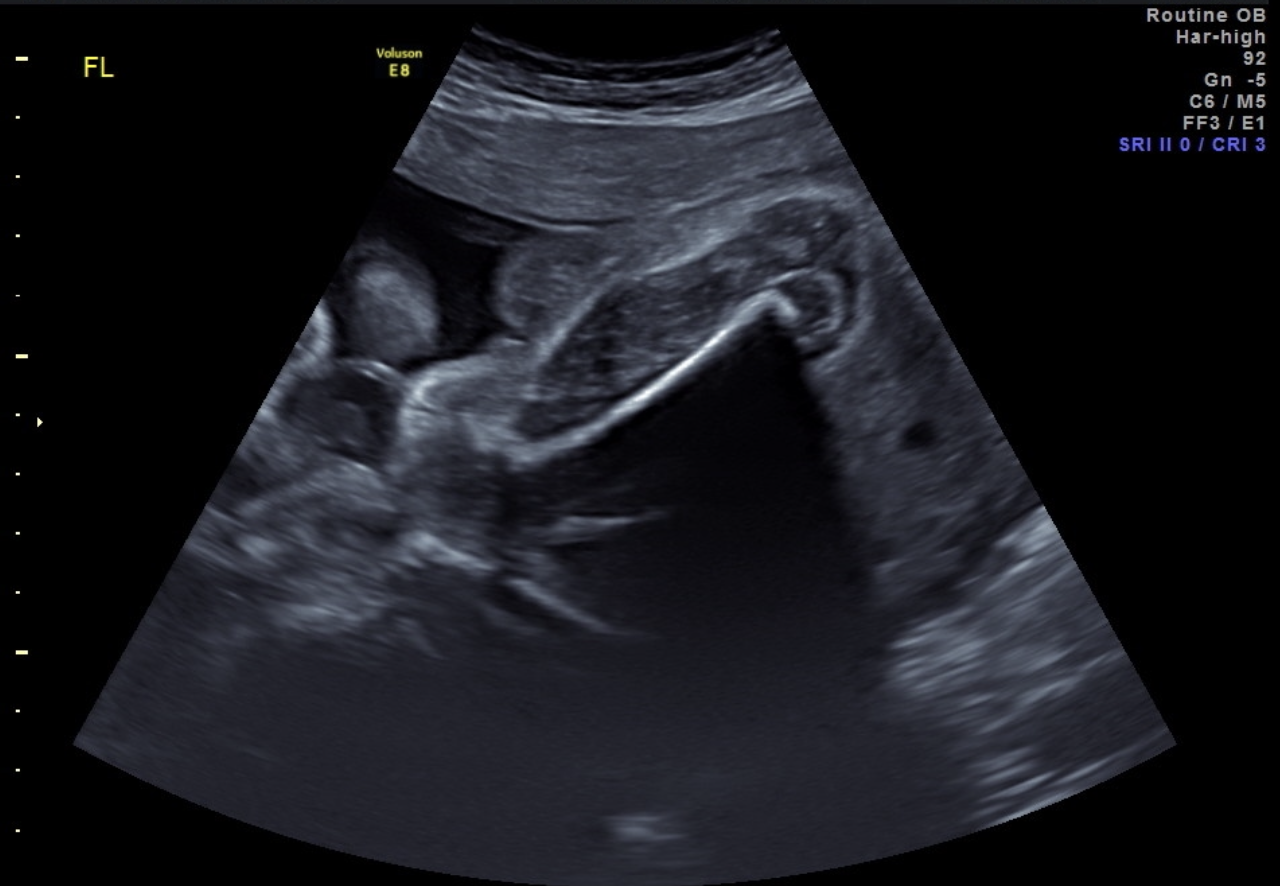

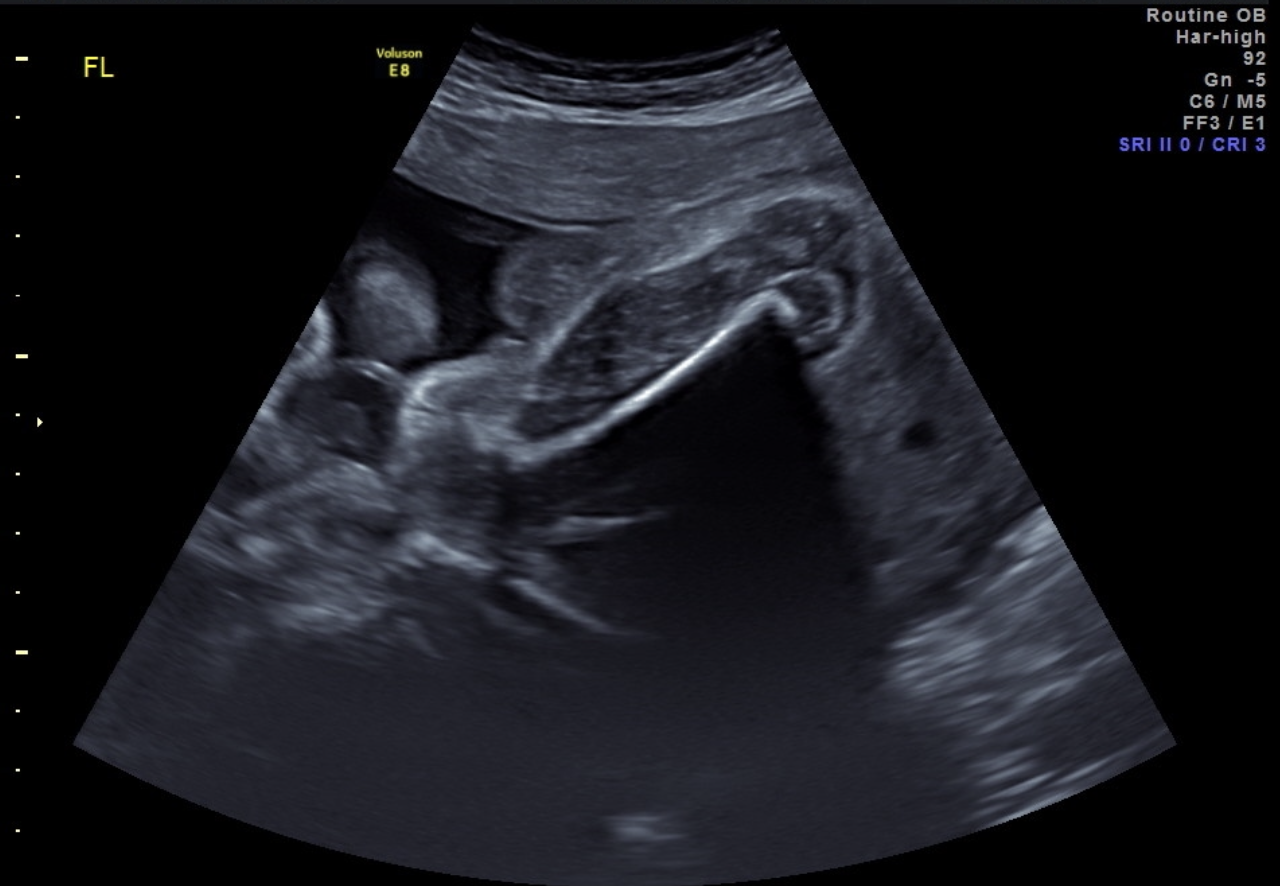

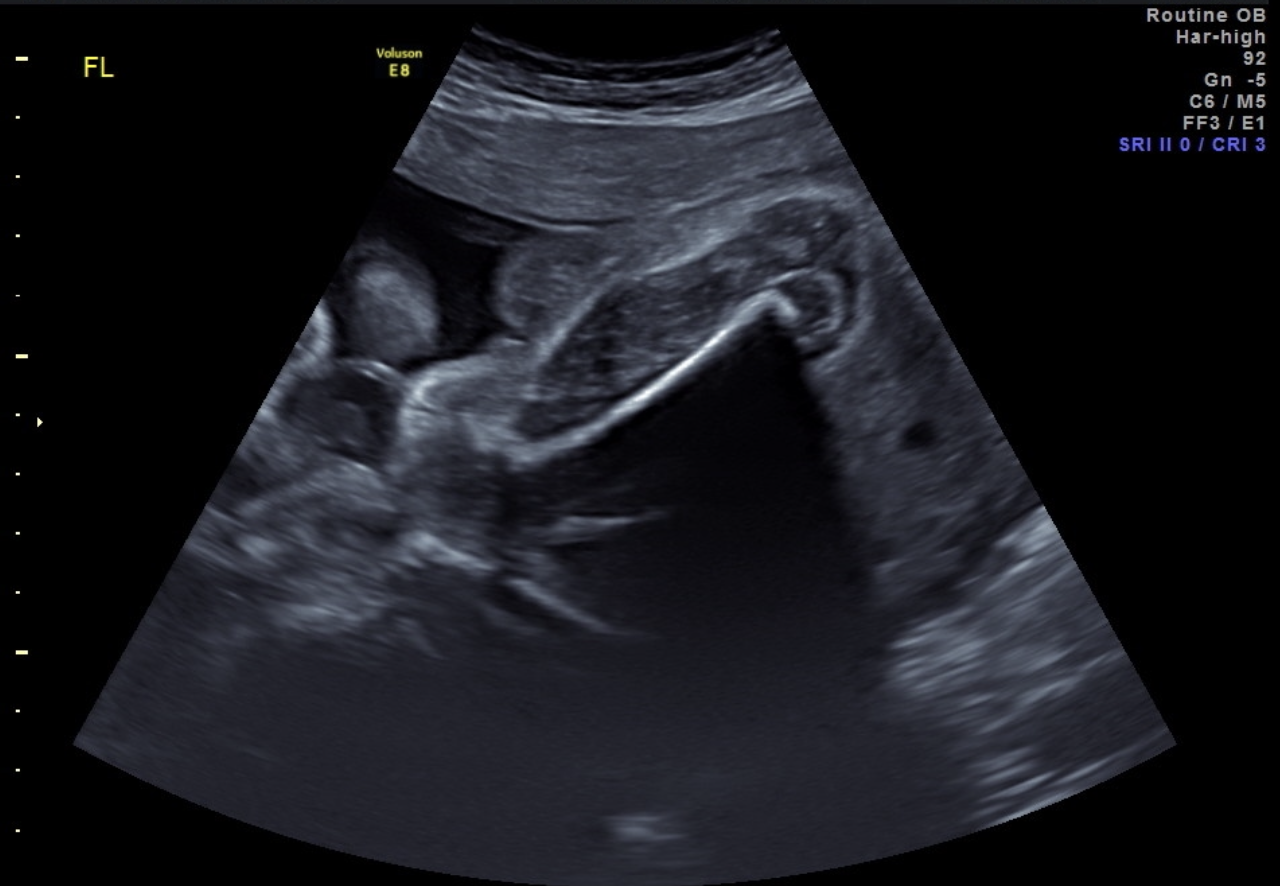

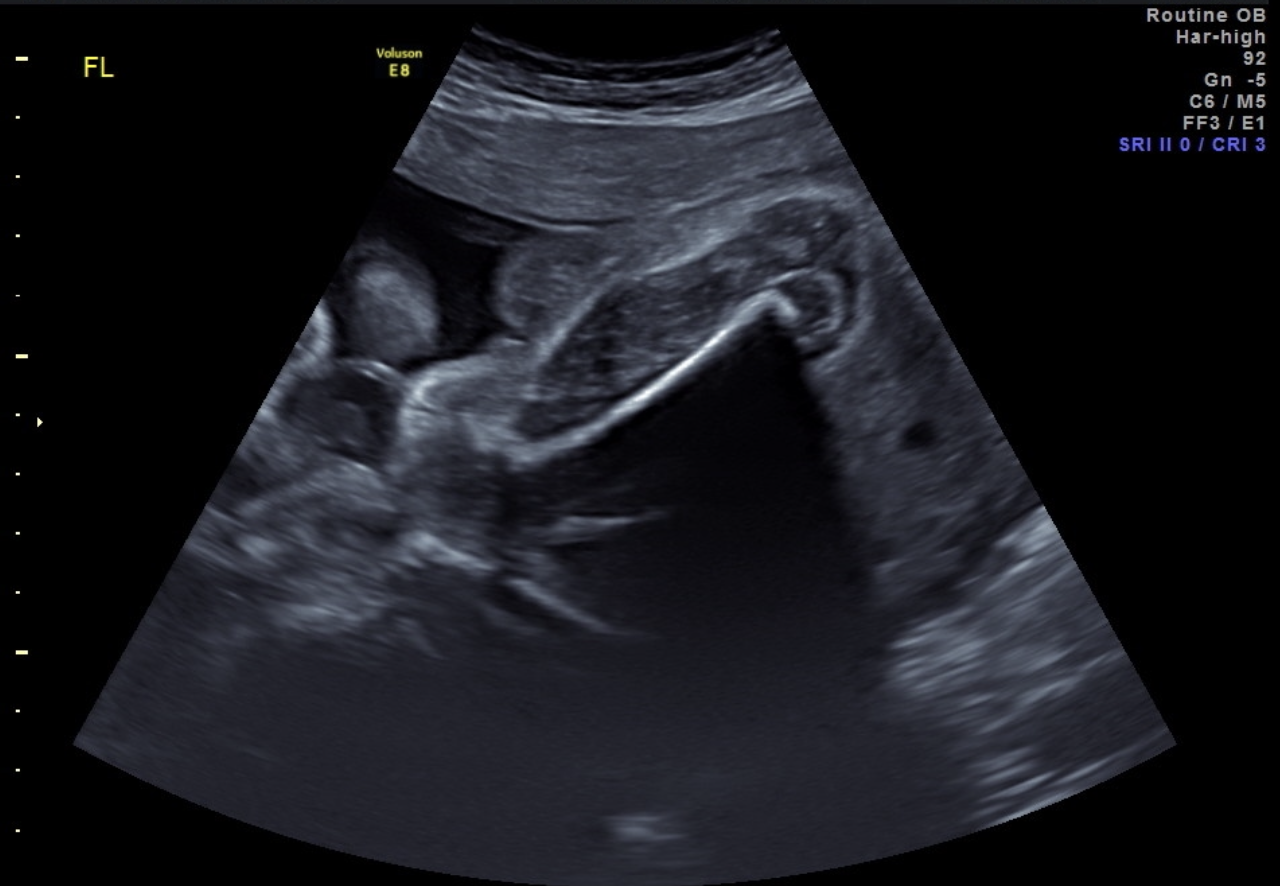

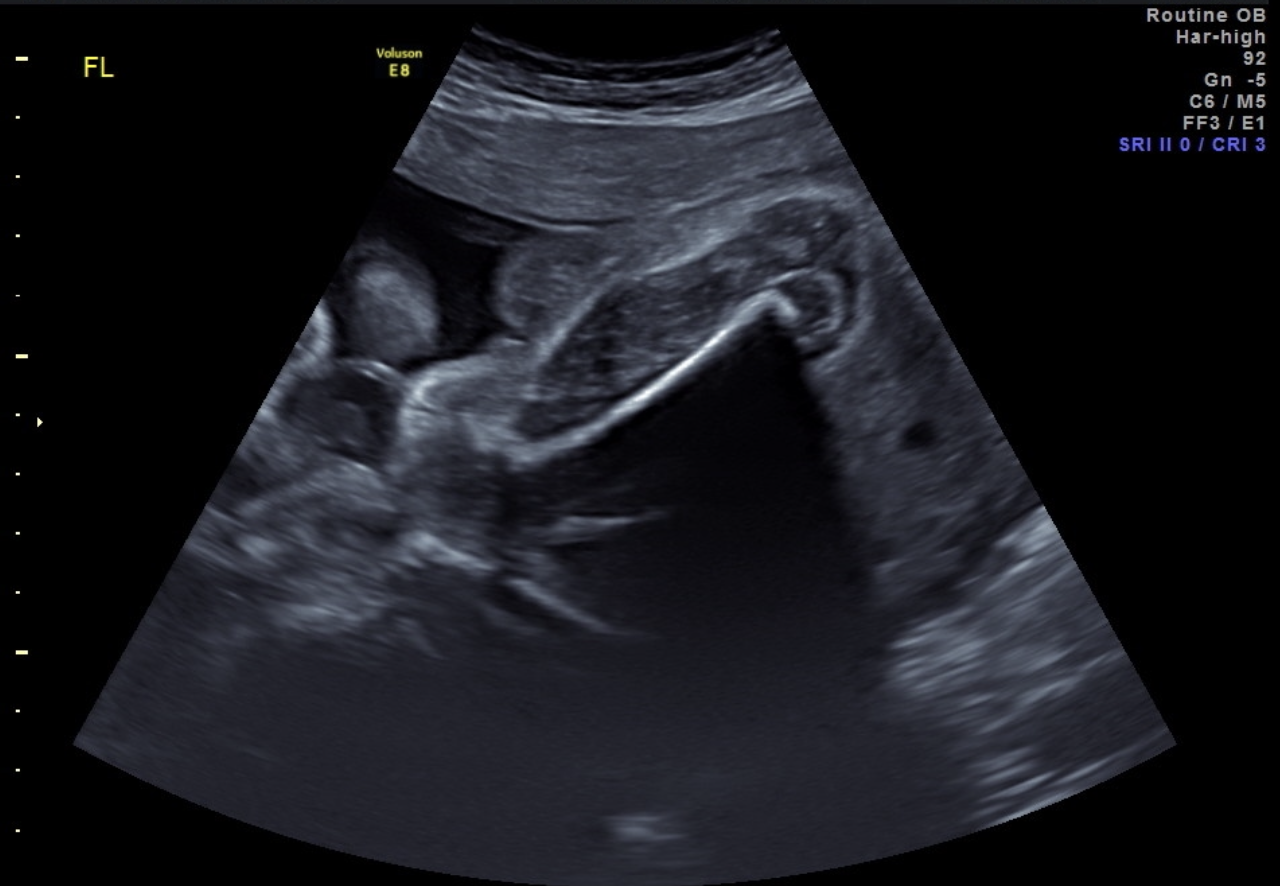

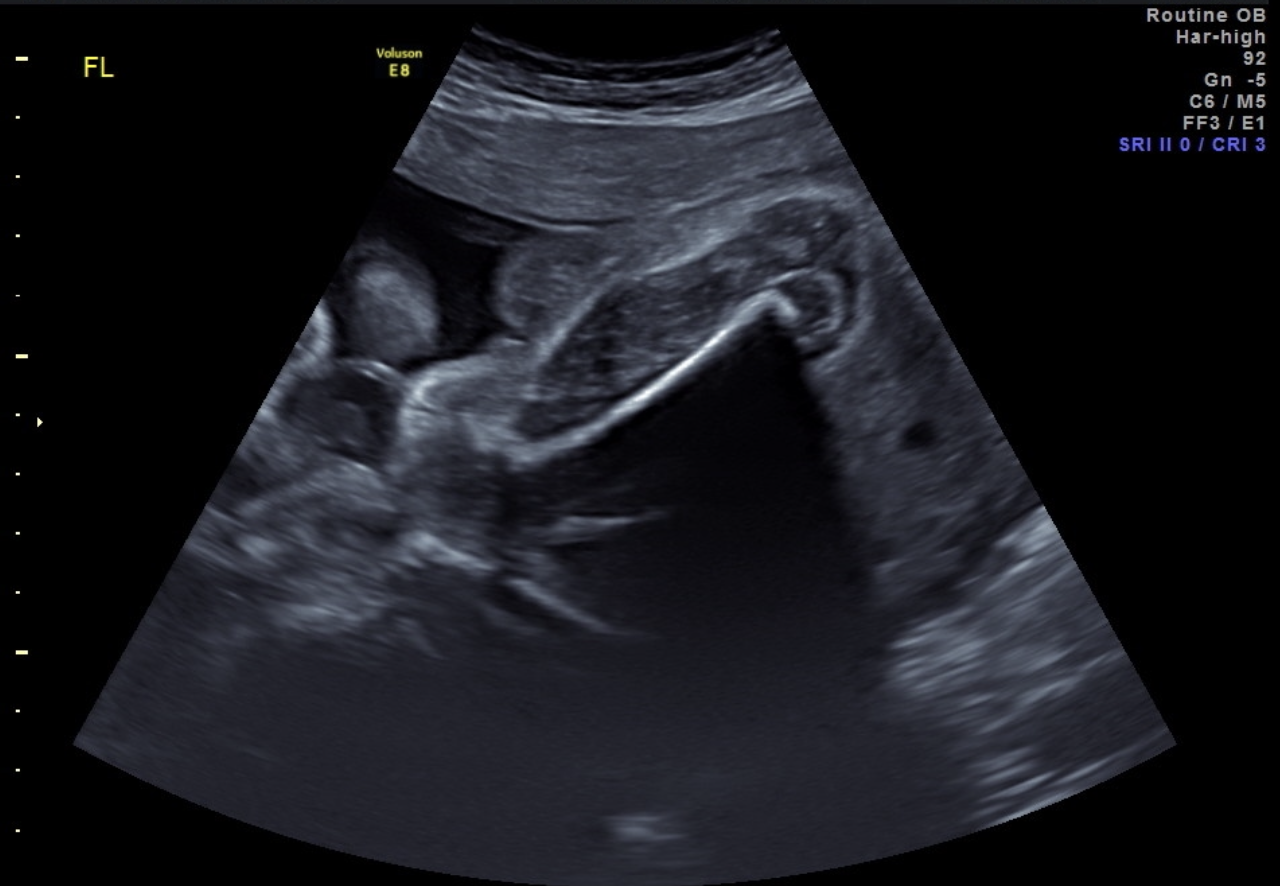

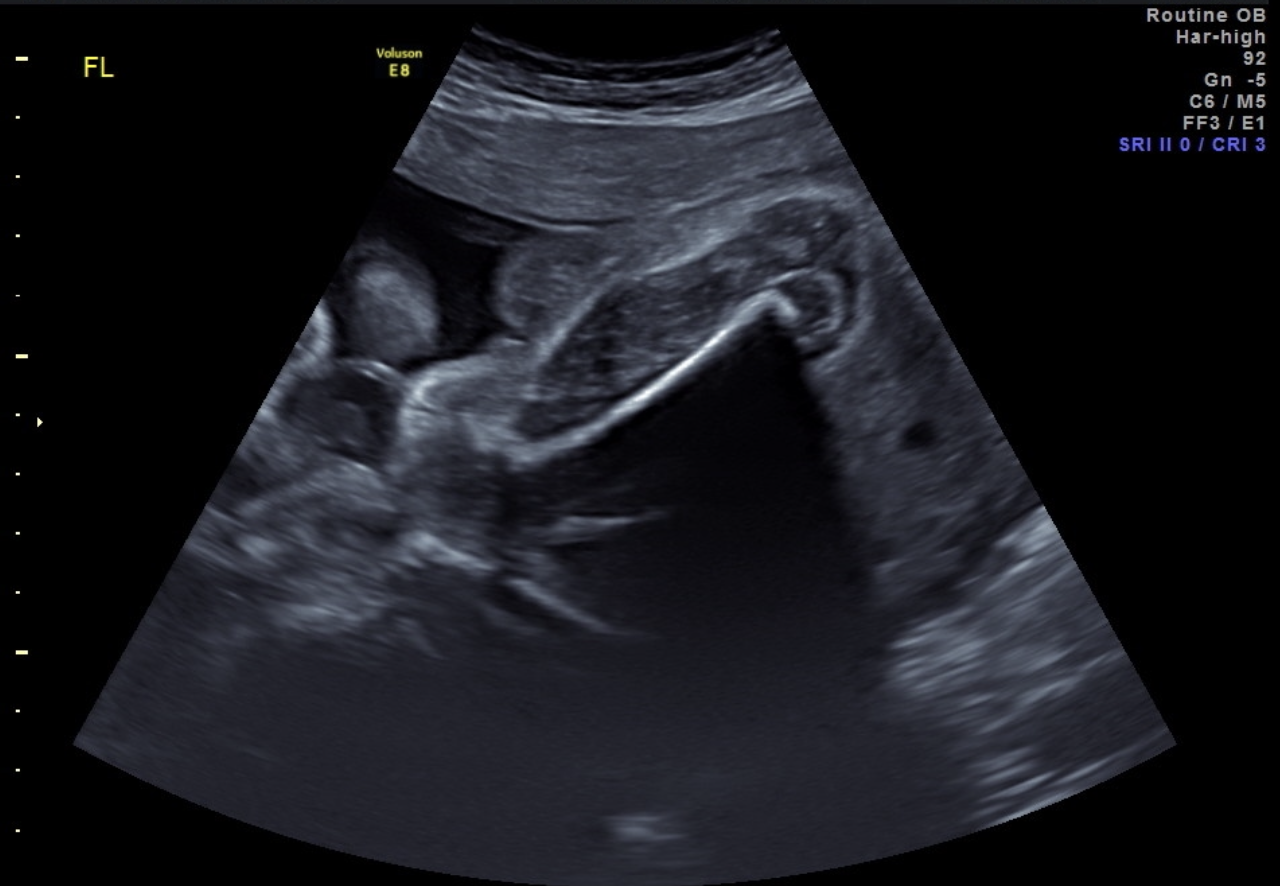

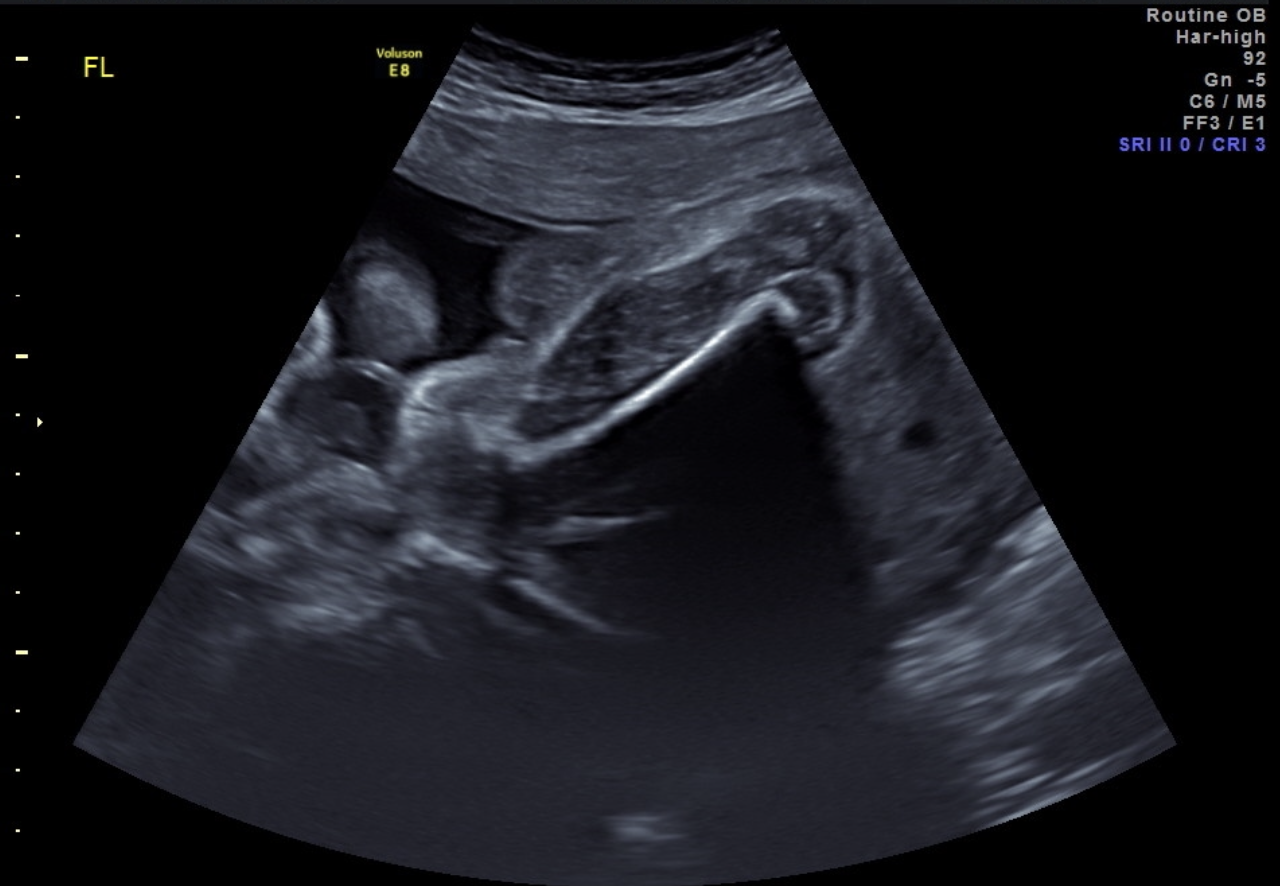

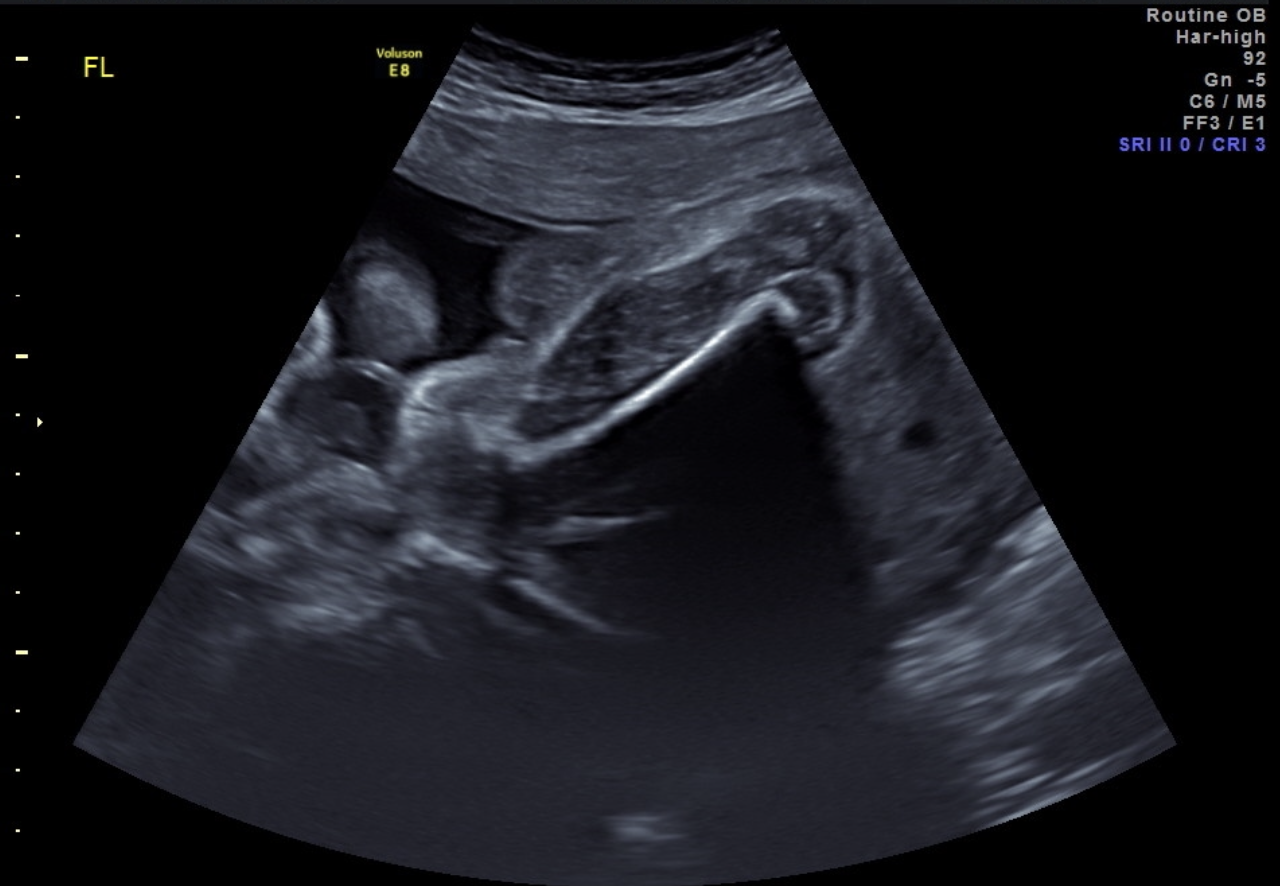

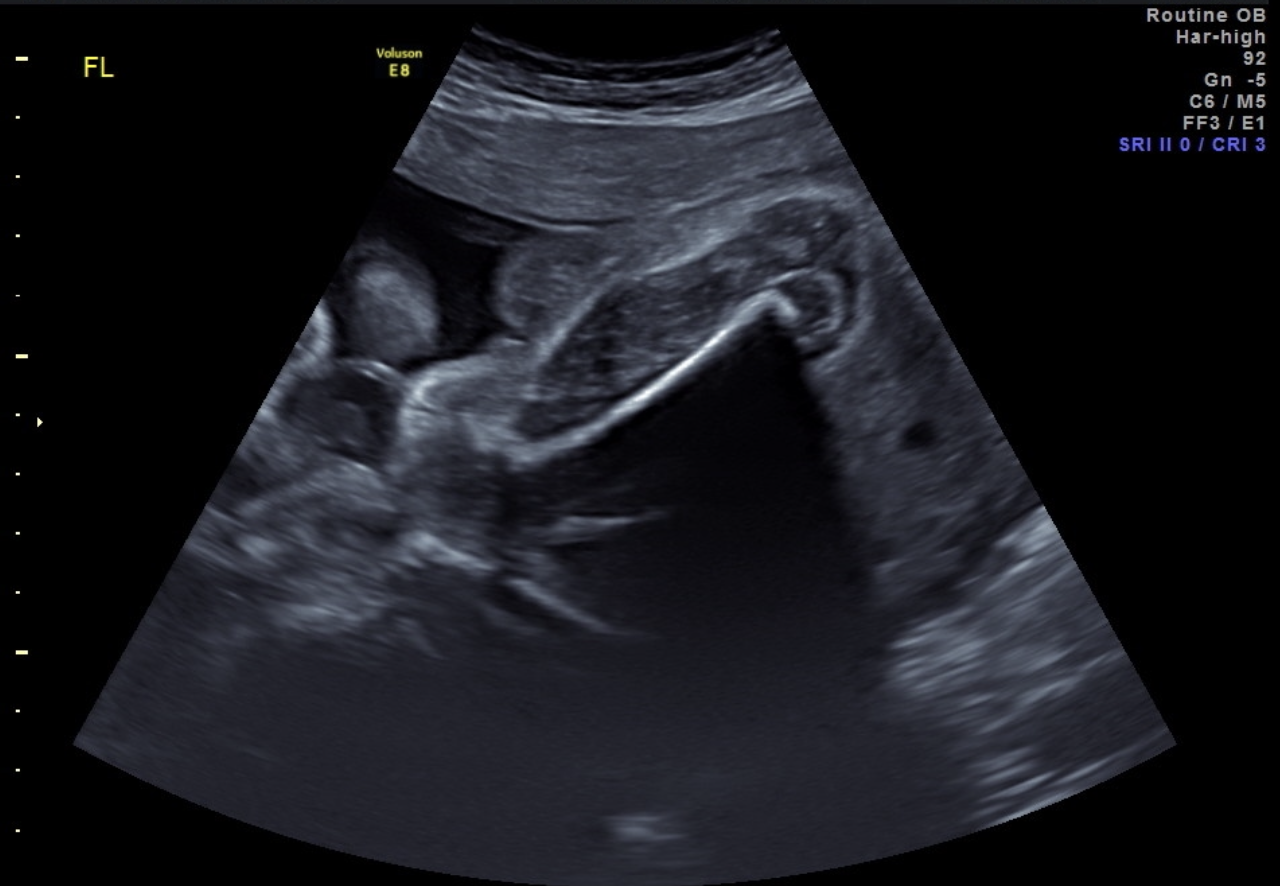

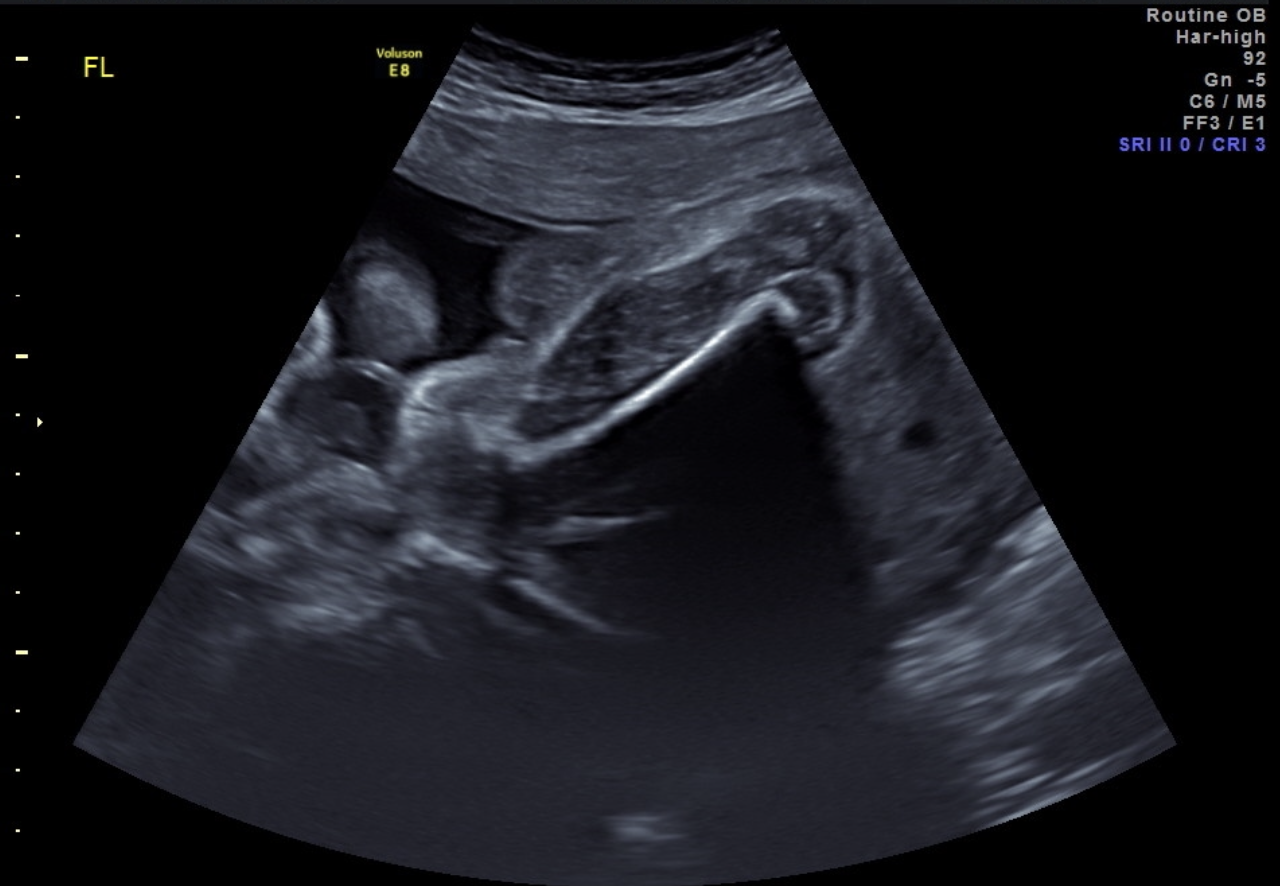

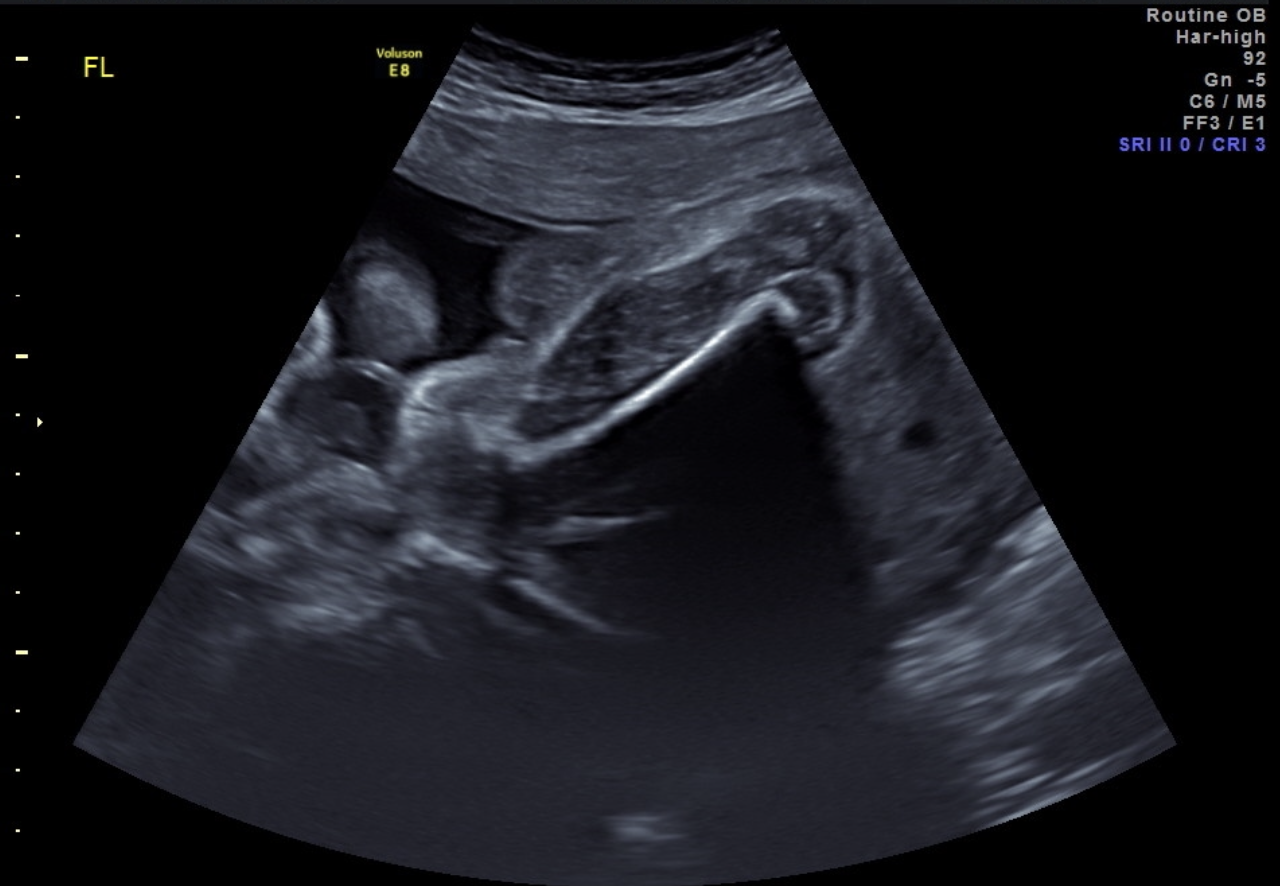

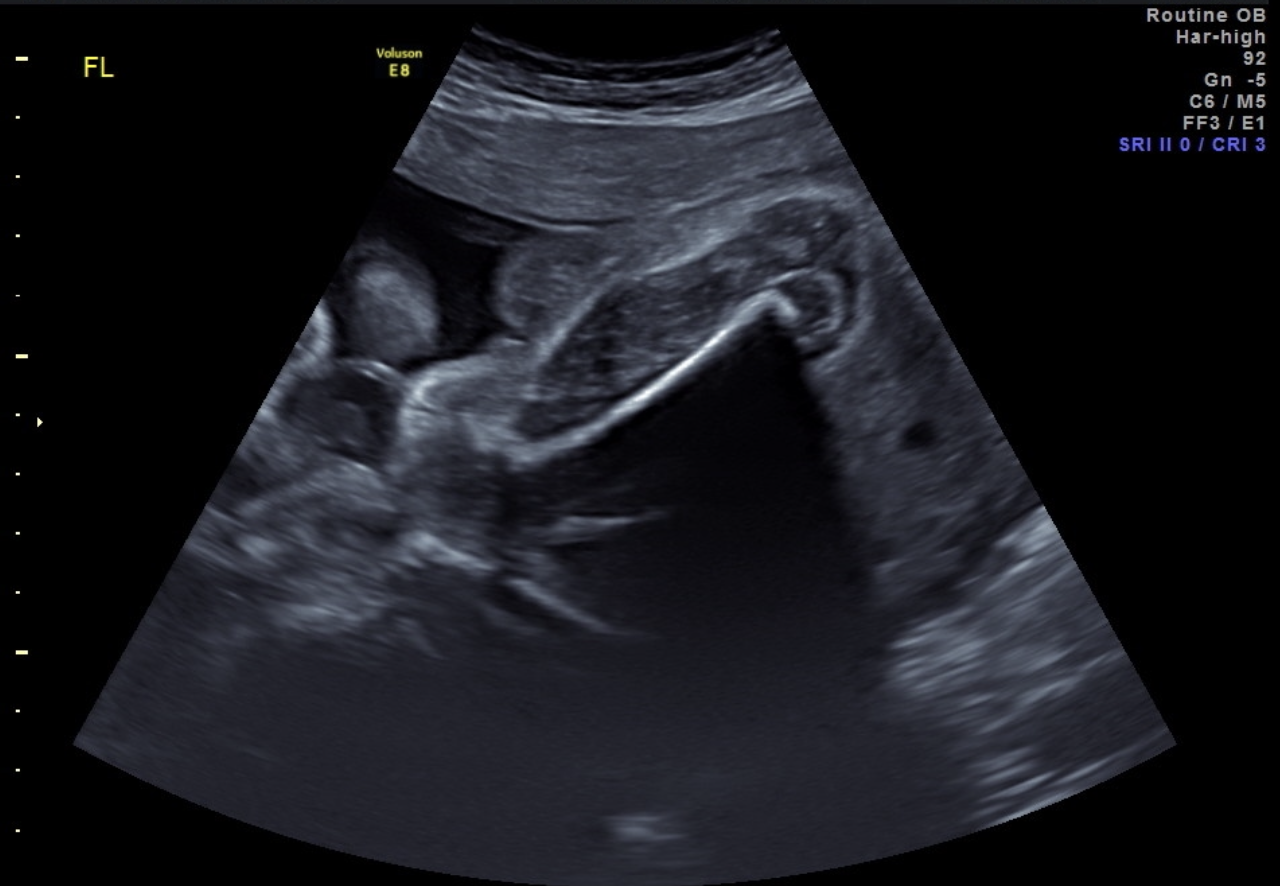

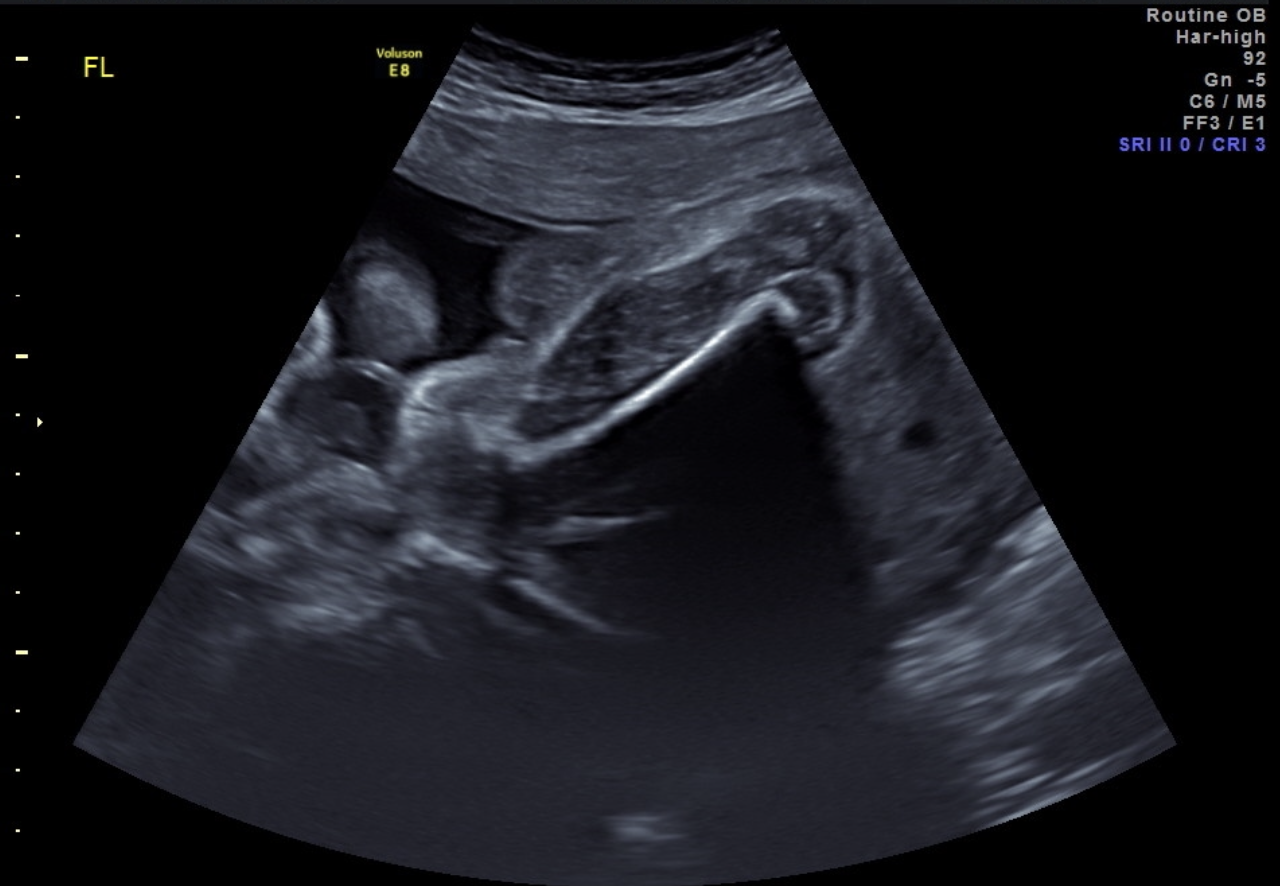

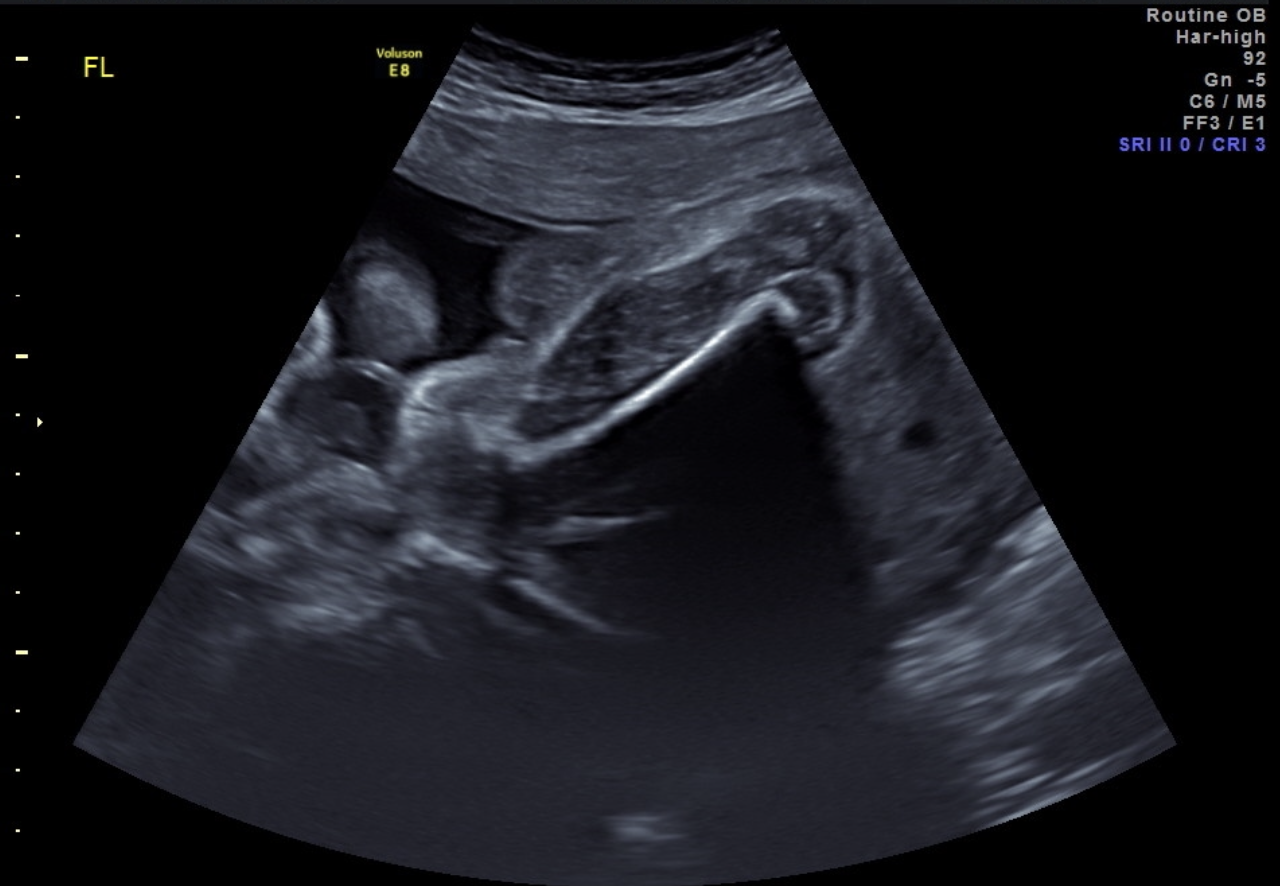

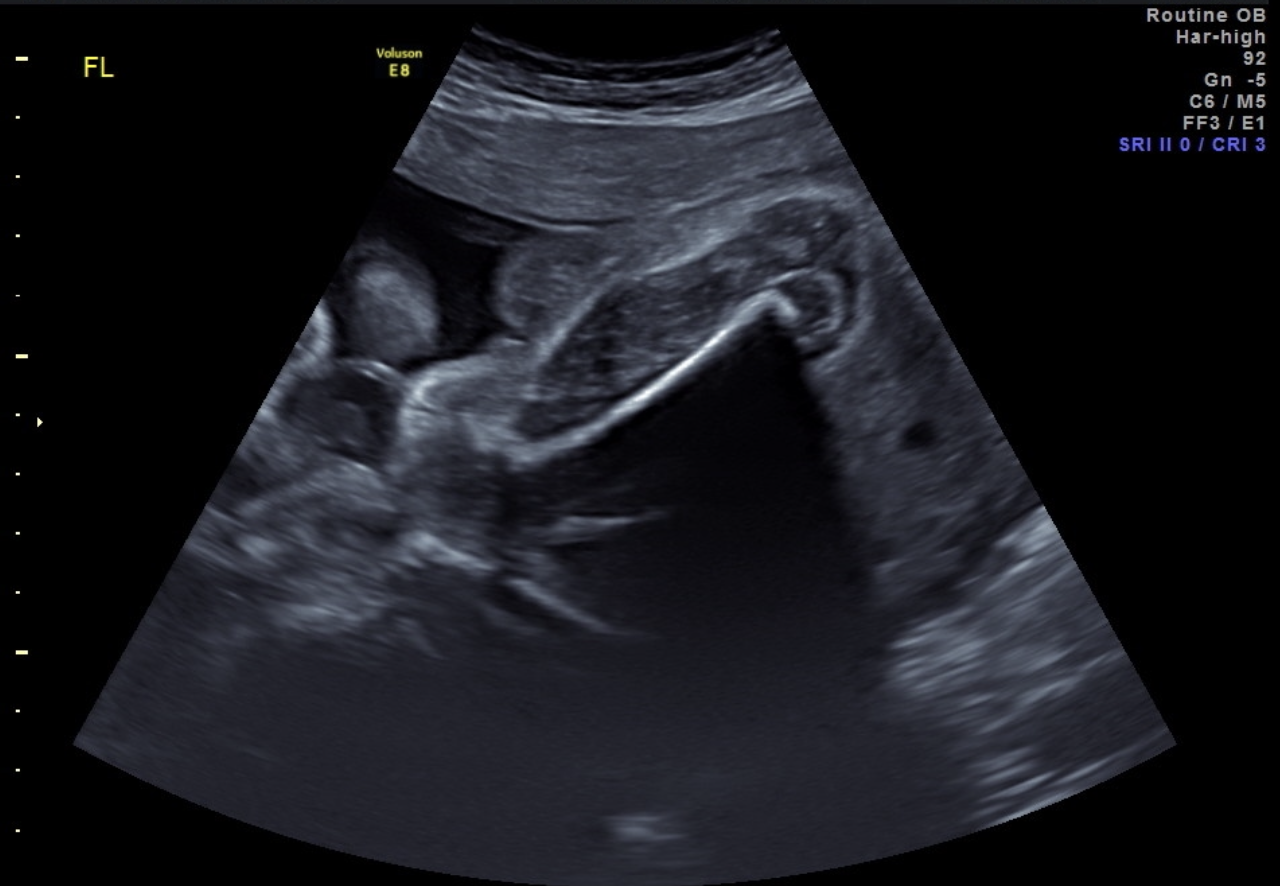

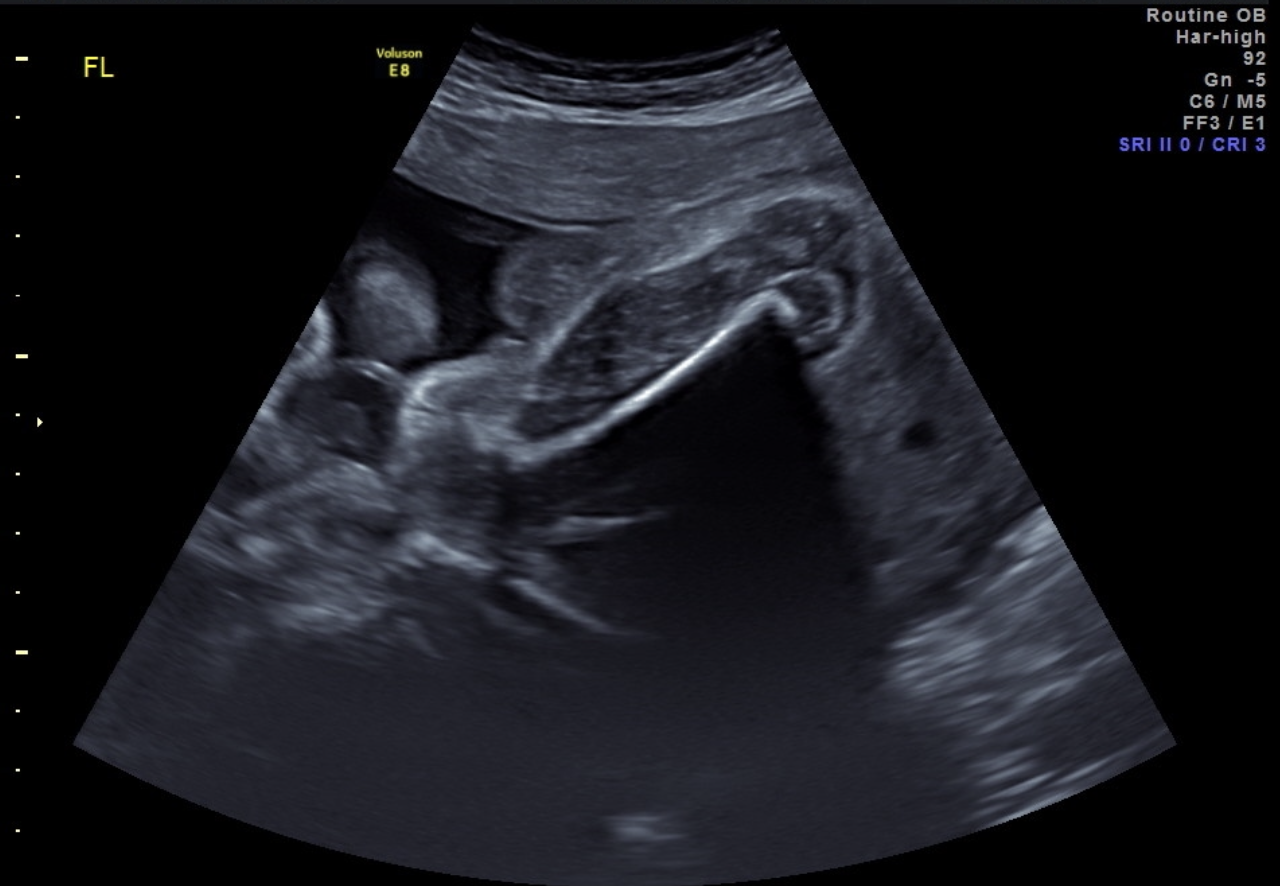

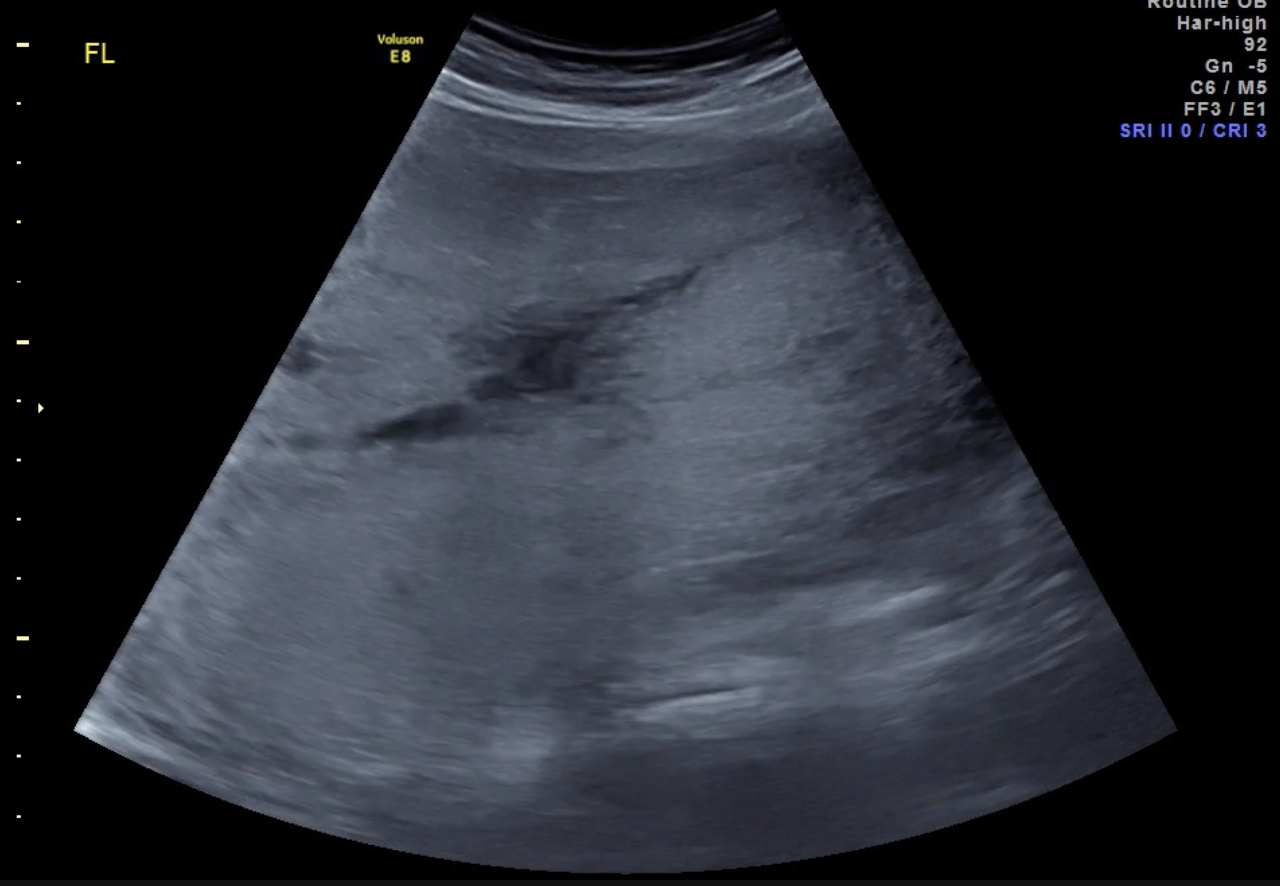


Normal fly-to

b = ~50 frames

c

a

## **Figure S2: Estimation error of the deep learning model vs. expert biometry expressed as a cumulative distribution function in the test set**


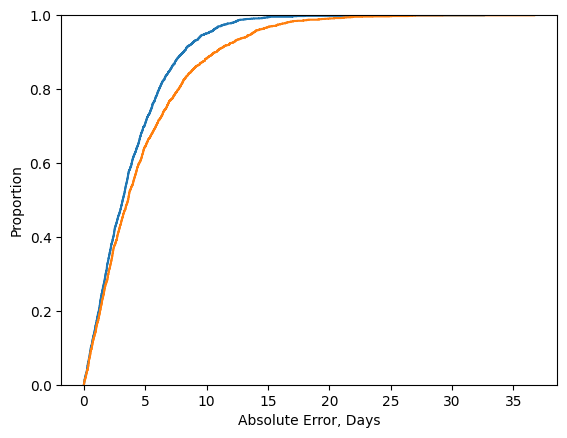


**Figure S3: Bland-Altman plot comparing deep learning model to expert biometry**


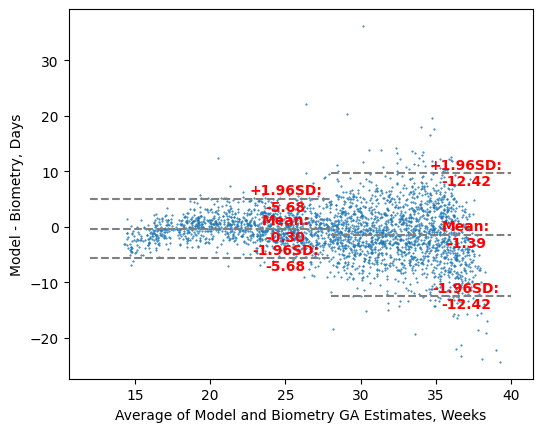


# **Supplemental Tables**

## **Table S1:** Ultrasound devices used

| **Ultrasound manufacturer and model** | **Training Set**  **(n = 5735)** | **Tuning Set**  **(n = 1448)** | **Test Set**  **(n = 2017)** |
| --- | --- | --- | --- |
| **Sonosite MTurbo** | 530 | 2005 | 512 |
| **GE Voluson S6** | 448 | 1841 | 582 |
| **GE Voluson E8** | 314 | 1237 | 796 |
| **GE LOGIQ e** | 129 | 590 | 118 |
| **GE LOGIQ C3 Premium** | 27 | 62 | 9 |

Each participant study visit involved data collection with commercial devices. N here represents number of studies.

GE = General Electric Healthcare, Zipf, Austria; Sonosite = SonoSite Inc, Bothell, WA, USA.

##

**Table S2:** Gestational age estimation of deep learning model compared to expert biometry in test set by biometry formulas and location

|  | | | **Test Set (n = 1,058)^a^** | | |
| --- | --- | --- | --- | --- | --- |
|  | | | Model | Hadlock IV | Intergrowth 21^st^ |
| **Overall** | MAE (SE), days | | 3.87 (0.07) | 5.22 (0.10) | 5.87 (0.12) |
|  | Diff (95% CI) | | Reference | -1.24 (-1.44, -1.04) | -2.00 (-2.21, -1.79) |
|  | RMSE (SE), days | | 5.01 (0.11) | 6.87 (0.15) | 7.83 (0.15) |
|  | 2^nd^ trimester^b^ | MAE (SE), days | 3.14 (0.09) | 3.28 (0.09) | 3.89 (0.12) |
|  |  | Diff (95% CI) | Reference | -0.14 (-0.33, 0.06) | -0.75 (-0.96, -0.55) |
|  | 3^rd^ trimester^c^ | MAE (SE), days | 4.44 (0.10) | 6.53 (0.16) | 7.41 (0.17) |
|  |  | Diff (95% CI) | Reference | -2.09 (-2.40, -1.78) | -2.97 (-3.30, -2.64) |
| **UNC** | MAE (SE), days | | 3.81 (0.10) | 4.05 (0.12) | 6.24 (0.18) |
|  | Diff (95% CI) | | Reference | -0.24 (-0.46, -0.03) | -2.44 (-2.78, -2.10) |
|  | RMSE (SE), days | | 4.90 (0.13) | 5.53 (0.18) | 8.38 (0.23) |
|  | 2^nd^ trimester^b^ | MAE (SE), days | 3.04 (0.10) | 2.89 (0.10) | 3.88 (0.15) |
|  |  | Diff (95% CI) | Reference | 0.15 (-0.05, 0.34) | -0.83 (-1.12, -0.55) |
|  | 3^rd^ trimester^c^ | MAE (SE), days | 4.76 (0.17) | 5.49 (0.22) | 9.19 (0.3) |
|  |  | Diff (95% CI) | Reference | -0.73 (-1.15, -0.31) | -4.42 (-5.05, -3.80) |
| **Zambia** | MAE (SE), days | | 3.93 (0.10) | 6.12 (0.16) | 5.51 (0.15) |
|  | Diff (95% CI) | | Reference | -2.19 (-2.51, -1.86) | -1.58 (-1.83, -1.32) |
|  | RMSE (SE), days | | 5.12 (0.16) | 7.94 (0.22) | 7.26 (0.20) |
|  | 2^nd^ trimester^b^ | MAE (SE), days | 3.3 (0.15) | 3.89 (0.15) | 3.91 (0.19) |
|  |  | Diff (95% CI) | Reference | -0.60 (-1.00, -0.20) | -0.62 (-0.89, -0.35) |
|  | 3^rd^ trimester^c^ | MAE (SE), days | 4.24 (0.13) | 7.20 (0.21) | 6.29 (0.19) |
|  |  | Diff (95% CI) | Reference | -2.98 (-3.38, -2.53) | -2.05 (-2.40, -1.70) |

^a^ Test set as described in Figure 2

^b^ 2^nd^ trimester is defined as 98 to 185 days

^c^ 3^rd^ trimester is defined as 186 days to 280 day

**Table S3:** Gestational age estimation of deep learning model on main test set with only one study included per participant

|  | **Test Set (n = 1,058)^a^** | | |
| --- | --- | --- | --- |
|  | Model | Biometry | Difference  (95% CI) |
| Mean Absolute Error (SE), days | 3.96 (0.10) | 4.93 (0.14) | -0.98 (-1.22, -0.73) |
| Root Mean Square Error (SE), days | 5.12 (0.16) | 6.61 (0.20) | -1.49 (-1.85, -1.14) |
| 2^nd^ trimester^b^  Mean Absolute Error (SE), days | 3.28 (0.13) | 3.43 (0.15) | -0.15 (-0.40, 0.09) |
| 3^rd^ trimester^c^  Mean Absolute Error (SE), days | 4.43 (0.14) | 5.99 (0.19) | -1.56 (-1.93, -1.19) |
| Absolute Error < 10 days (SE), % | 94.5 (0.7) | 88.0 (1.0) | 6.5 (4.5, 8.6) |
| UNC MAE, days | 3.87 (0.15) | 4.20 (0.19) | -0.33 (-0.68, 0.02) |
| Zambia MAE, days | 4.01 (0.13) | 5.40 (0.18) | -1.39 (-1.72, -1.06) |

^a^ Test set as described in Figure 2

^b^ 2^nd^ trimester is defined as 98 to 185 days

^c^ 3^rd^ trimester is defined as 186 days to 280 **days**

## **Table S4:** Gestational age estimation of deep learning model compared to expert sonographer – sensitivity analysis

|  | **‘Novice’ Sensitivity Test Set (n = 1,058)^a^** | | |
| --- | --- | --- | --- |
|  | Model^*^ | Biometry | Difference (95% CI) |
| **Mean Absolute Error (SE), days** | 4.07 (0.08) | 4.80 (0.10) | -0.73 (-0.91, -0.54) |
| **Root Mean Square Error (SE), days** | 5.30 (0.11) | 6.47 (0.14) | -1.17 (-1.44, -0.91) |
| **2^nd^ trimester^b^**  **Mean Absolute Error (SE), days** | 3.25 (0.09) | 3.28 (0.01) | -0.03 (-0.21, 0.14) |
| **3^rd^ trimester^c^**  **Mean Absolute Error (SE), days** | 4.71 (0.11) | 5.98 (0.14) | -1.27 (-1.56, -0.97) |
| **Absolute Error < 10 days (SE), %** | 93.8 (0.5) | 88.3 (0.7) | 5.5 (3.9, 7.0) |
| **UNC MAE, days** | 4.06 (0.11) | 4.05 (0.12) | 0.01 (-0.22, 0.24) |
| **Zambia MAE, days** | 4.08 (0.11) | 5.51 (0.15) | -1.43 (-1.71, -1.15) |

^a^ Truncated sensitivity test set as described in S1

^b^ 2^nd^ trimester is defined as 98 to 185 days

^c^ 3^rd^ trimester is defined as 186 days to 280 days
